# Multifactorial assessment of Parkinson’s disease course and outcomes using trajectory modeling in a multiethnic, multisite cohort – extension of the LONG-PD study

**DOI:** 10.3389/fnagi.2023.1240971

**Published:** 2023-09-26

**Authors:** Bruce A. Chase, Rejko Krueger, Lukas Pavelka, Sun Ju Chung, Jan Aasly, Efthimios Dardiotis, Ashvini P. Premkumar, Bernadette Schoneburg, Ninith Kartha, Navamon Aunaetitrakul, Roberta Frigerio, Demetrius Maraganore, Katerina Markopoulou

**Affiliations:** ^1^Health Information Technology, NorthShore University HealthSystem, Evanston, IL, United States; ^2^Translational Neuroscience, Luxembourg Centre for Systems Biomedicine (LCSB), University of Luxembourg, Belvaux, Luxembourg; ^3^Transversal Translational Medicine, Luxembourg Institute of Health (LIH), Strassen, Luxembourg; ^4^Centre Hospitalier de Luxembourg (CLG), Luxembourg, Luxembourg; ^5^Parkinson’s Research Clinic, Centre Hospitalier de Luxembourg (CHL), Luxembourg, Luxembourg; ^6^Department of Neurology, Asan Medical Center, University of Ulsan College of Medicine, Seoul, Republic of Korea; ^7^Department of Neurology, St. Olav’s Hospital, Trondheim, Norway; ^8^Department of Neuroscience, Norwegian University of Science and Technology, Trondheim, Norway; ^9^Department of Neurology, University of Thessaly, University Hospital of Larissa, Larissa, Greece; ^10^Department of Neurology, NorthShore University HealthSystem, Evanston, IL, United States; ^11^Department of Neurology, Tulane University, New Orleans, LA, United States; ^12^Department of Neurology, University of Chicago Pritzker School of Medicine, Chicago, IL, United States

**Keywords:** longitudinal monitoring, Parkinson’s disease, group-based-trajectory model, motor symptoms, non-motor symptoms, disease outcomes

## Abstract

**Background:**

The severity, progression, and outcomes of motor and non-motor symptoms in Parkinson’s disease (PD) are quite variable. Following PD cohorts holds promise for identifying predictors of disease severity and progression.

**Methods:**

PD patients (*N* = 871) were enrolled at five sites. Enrollment occurred within 5 years of initial motor symptom onset. Disease progression was assessed annually for 2-to-10 years after onset. Group-based trajectory modeling was used to identify groups differing in disease progression. Models were developed for UPDRS-III scores, UPDRS-III tremor and bradykinesia-rigidity subscores, Hoehn & Yahr (H&Y) stage, Mini-Mental Status Exam (MMSE) scores, and UPDRS-III, H&Y and MMSE scores considered together. Predictors of trajectory-group membership were modeled simultaneously with the trajectories. Kaplan–Meier survival analysis evaluated survival free of PD outcomes.

**Results:**

The best fitting models identified three groups. One showed a relatively benign, slowly progressing trajectory (Group 1), a second showed a moderate, intermediately progressing trajectory (Group 2), and a third showed a more severe, rapidly progressing trajectory (Group 3). Stable trajectory-group membership occurred relatively early in the disease course, 5 years after initial motor symptom. Predictors of intermediate and more severe trajectory-group membership varied across the single variable models and the multivariable model jointly considering UPDRS-III, H&Y and MMSE scores. In the multivariable model, membership in Group 2 (28.4% of patients), relative to Group 1 (50.5%), was associated with male sex, younger age-at-onset, fewer education-years, pesticide exposure, absence of reported head injury, and akinetic/rigid subtype at initial presentation. Membership in Group 3 (21.3%), relative to Group 1, was associated with older age-at-onset, fewer education-years, pesticide exposure, and the absence of a tremor-predominant subtype at initial presentation. Persistent freezing, persistent falls, and cognitive impairment occurred earliest and more frequently in Group 3, later and less frequently in Group 2, and latest and least frequently in Group 1. Furthermore, autonomic complications, dysphagia, and psychosis occurred more frequently in Groups 2 and 3 than in Group 1.

**Conclusion:**

Modeling disease course using multiple objective assessments over an extended follow-up duration identified groups that more accurately reflect differences in PD course, prognosis, and outcomes than assessing single parameters over shorter intervals.

## Introduction

Parkinson’s disease (PD) is a progressive neurodegenerative disease with an insidious onset and a long pre-symptomatic and symptomatic course. Disease onset is currently defined by the appearance of the cardinal motor symptoms, i.e., resting tremor, bradykinesia, rigidity, and postural instability. Manifestations of bradykinesia include hypokinetic dysarthria, oropharyngeal dysphagia, micrographia, hypomimia, reduced dexterity, stooped posture, difficulty arising from a chair/bed, shuffling gait, and general slowness of movement. Different motor disease subtypes have been described including tremor-predominant, akinetic/rigid-predominant and mixed subtypes (Schiess et al., 2000; Thenganatt and Jankovic, 2014). In addition to the motor symptoms, non-motor features, including cognitive dysfunction, anosmia, anxiety, depression, sleep disorders, and autonomic dysfunction may be present either alone or in varying combinations, and a non-motor dominant subtype of PD has been identified (Pavelka et al., 2022).

The symptomatic phase is preceded by a long prodromal phase (Mahlknecht et al., 2022; Borghammer, 2023; Elliott et al., 2023; Joza et al., 2023; Leite Silva et al., 2023). The combination and severity of prodromal symptoms can affect disease severity and progression after motor symptom onset.

The temporal profile of motor and non-motor symptom appearance and progression is rather variable. As discussed below, different cohorts have been followed longitudinally for varying lengths of time to identify predictors of disease progression. In the Primary Progression Markers Initiative (PPMI) cohort, higher baseline (Movement Disorders Society-Unified Parkinson’s Disease Rating Scale [MDS-UPDRS]) motor score, male sex, and increased age, as well as a novel PD-specific epistatic interaction, were indicative of faster motor progression (Latourelle et al., 2017). In the same cohort, higher baseline non-motor scores were associated with female sex and a more severe motor phenotype. Longitudinal increase in non-motor score severity was associated with older age and lower cerebrospinal fluid (CSF) levels of Aβ1–42 at baseline (Schrag et al., 2017; Simuni et al., 2018). In addition, the postural instability gait disorder (PIGD) subtype was characterized by more severe disease manifestations at diagnosis, greater cognitive progression, and more frequent psychosis than in tremor-predominant patients (Aleksovski et al., 2018).

Analysis of the Tracking Parkinson’s and Discovery cohorts reported four clusters: one with fast motor progression and symmetrical motor disease, poor olfaction, cognitive impairment and postural hypotension; a second with mild motor and non-motor disease and intermediate motor progression; a third with severe motor disease, poor psychological well-being and poor sleep with an intermediate motor progression; and a fourth with slow motor progression with tremor-dominant, unilateral disease (Lawton et al., 2018).

In the *De Novo* Parkinson cohort (DeNoPa; Mollenhauer et al., 2019), baseline predictors of more rapid progression of motor symptoms included male sex, orthostatic blood pressure drop, diagnosis of coronary artery disease, arterial hypertension, elevated serum uric acid, and elevated CSF neurofilament light chain. Predictors of cognitive decline included previous heavy alcohol use, diabetes mellitus, arterial hypertension, elevated periodic limb movement index during sleep, decreased hippocampal volume measured by magnetic resonance imaging (MRI), and higher serum uric acid, C-reactive protein, high-density-lipoprotein (HDL) cholesterol, and glucose levels at baseline. In this cohort, faster disease progression was associated with cardiovascular risk factors, poor diabetes control, higher serum uric acid levels, and inflammation.

A more recent comparison of the DeNoPa cohort and PPMI cohorts showed similar slopes of progression in both. Faster progression from baseline was associated with higher activities of daily living (ADL) scores and rigidity/bradykinesia subscores. In addition, freezing, and rigidity were predictors of faster deterioration in both cohorts (Bartl et al., 2022).

Comparing predictors of disease progression identified in the previously reported longitudinal cohorts reveals partial overlap but also differences. The lack of uniformity of predictors identified in the different cohorts may reflect differences in patient population characteristics, including genetic variation, contributing to the development and progression of PD, but also variability in how PD was assessed and different duration of follow-up. To improve our understanding of patterns of disease progression, identify predictors associated with these patterns, and determine how these patterns are related to clinically significant milestones of disease progression, we extended our previous longitudinal analysis of PD patients (Markopoulou et al., 2020). In this analysis, we included an additional cohort, the LuxPark cohort, which was assessed using the same protocol as in the original study, and analyzed annual follow-up data obtained over a period of 2-to-10 years from the initial motor symptom. Three groups showing different patterns of disease progression were identified in group-based trajectory models (GBTMs) using UPDRS-III total score, tremor- and bradykinesia/rigidity subscores, Hoehn & Yahr (H&Y) stage, Mini-Mental Status Exam (MMSE) score, and UPDRS-III, H&Y and MMSE scores considered jointly. Assignment to a trajectory group remained stable 5 years after the initial motor symptom, at which time misclassification was low. In addition, we performed survival analysis to determine how the appearance of debilitating symptoms including dyskinesias, motor fluctuations, persistent falls, persistent freezing, dysphagia, persistent orthostatism, persistent urinary incontinence, cognitive impairment, psychosis, REM sleep behavior disorder (RBD), and impulse control disorder (ICD) were associated with different group trajectories.

## Materials and methods

### Patients and clinical data collection

Five sites participating within the Genetic Epidemiology of Parkinson’s Disease (GEoPD) consortium (Farrer et al., 2021) contributed longitudinal data on patients with clinically probable or clinically definite PD (Bower criteria, Bower et al., 2000). These were: (1) Department of Neurology, St. Olav’s Hospital, The Norwegian University of Science and Technology, Trondheim, Norway (NUST, *N* = 77); (2) Asan Medical Center, University of Ulsan College of Medicine, Seoul, South Korea (ASAN, *N* = 270); (3) Department of Neurology, University Hospital of Larissa, University of Thessaly, Larissa, Greece (UT, *N* = 13); (4) *The Luxembourg Parkinson’s Study*, Luxembourg Institute of Health, Laboratoire National de Santé, Centre Hospitalier de Luxembourg, and Luxembourg Centre for Systems Biomedicine, University of Luxembourg, Belval, Luxembourg (LuxPark, *N* = 103); and (5) *The DodoNA Project: DNA Predictions to Improve Neurological Health*, Department of Neurology, NorthShore University HealthSystem, Evanston, IL USA (DodoNA, *N* = 408). Four sites (NUST, ASAN, UT, and DodoNA) had contributed data to previous analyses of LONG-PD data presented in Markopoulou et al., (2020). The current analyses included previously obtained data from the NUST site, new data from the LuxPark site and additional data collected from 2019 to 2022 by the ASAN, UT, and DodoNA sites.

For details of the study protocol, see Markopoulou et al. (2020). Salient features are summarized below. Investigators at each site entered initial- and annual-follow-up visit data into REDCap, a web-based database, or, due to data governance requirements, securely transmitted a spreadsheet containing their data. This cohort included both previously diagnosed and treatment naïve PD patients. Patients were enrolled at an initial clinical encounter if their first motor symptom occurred within 5 years of that encounter and the encounter resulted in a diagnosis of clinically probable or definite PD (Bower et al., 2000). In this study, we analyzed annual follow-up data through 10 years after initial motor symptom onset. Although patients were retained if their diagnosis at a subsequent annual visit changed to PD with dementia (PDD), they were excluded if their diagnosis changed to Lewy body dementia, drug-induced parkinsonism, multiple system atrophy, post-encephalitic parkinsonism, cerebrovascular disease with parkinsonism features, progressive supranuclear palsy, cortical basal syndrome, or parkinsonism-unspecified.

A family history of PD, dementia, or tremor was defined as having at least one first- or second-degree relative with the disease. Pesticide exposure, including any past or present, hobby, and/or occupational use, was self-reported. Head injury was self-reported and documented if the injury resulted in loss of consciousness or required medical attention. Cognitive status was assessed at the initial visit and annually thereafter. If a patient was cognitively impaired, a legally authorized representative provided relevant information. As previously described (Markopoulou et al., 2020), “MMSE score” reflects actual MMSE scores or Montreal Cognitive Assessment (MoCA) or Short Test of Mental Status (STMS) scores converted to MMSE scores using published normograms that allow interconversion (Roalf et al., 2013; van Steenoven et al., 2014; Townley et al., 2019), and UPDRS-III refers to MDS-UPDRS-III or UPDRS-III scores.

### Disease trajectories

We used group-based trajectory modeling, a semi-parametric, model-based clustering method, to identify latent groups with similar longitudinal progression of PD (Jones and Nagin, 2007; Nagin and Odgers, 2010). As done previously (Markopoulou et al., 2020), trajectories were calculated starting from time point zero, defined as year of initial motor symptom appearance, reported at the initial clinical encounter. Trajectories assessing progression of motor impairment were generated for total UPDRS-III (motor) scores (*N* = 871), Hoehn & Yahr (H&Y) stage (*N* = 871), and using individual-item scores on the UPDRS-III (available only at the ASAN and DodoNA sites) (*N* = 678): UPDRS-III tremor subscores (the sum of questions 3–9) and bradykinesia-rigidity subscores (the sum of questions 1–2, 10–25, 27). Trajectories assessing cognitive function impairment were generated using Mini-Mental Status Exam (MMSE, *N* = 870, data missing for one patient) scores. To determine whether multiple assessment measures considered jointly would provide a more accurate characterization of disease progression and reliable association with disease outcomes, latent groups of patients were also identified by simultaneously assessing the trajectories of three variables: UPDRS-III, H&Y, and MMSE (*N* = 870).

We report the trajectories obtained when the *traj* plug-in in Stata/BE 17.0 (Jones and Nagin, 2013) was used to fit longitudinal data from the above six assessment measures to finite-mixture models. Models fit longitudinal data from patients who had between two and nine annual visits (maximum *N* = 871). The link function between the time and the assessment variable was censored-normal (a tobit model). Dropout was modeled for single-measure outcomes, but could not be included in the three-variable model jointly assessing UPDRS-III, H&Y, and MMSE.

In a basic trajectory model, the probability of trajectory group membership follows the multinomial logistic function. To understand which baseline characteristics were associated with trajectory-group membership, we generated models with predictors of group-membership probability relative to a reference group. In all analyses, the reference group was defined as the group with the most benign disease course, so that the odds ratios (OR) and 95% confidence intervals (95% CIs) we report are relative to that group. In these models, the parameters measuring the association of the predictor variables with trajectory-group membership were estimated jointly with the parameters specifying the shapes of the trajectory. Continuous predictors were age at first motor symptom onset (AAO) and, in models utilizing MMSE score, years of education. The evaluated binomial predictors were restricted to variables present in at least 5% of patients. These included sex; history of pesticide exposure, head-injury, or diabetes; the presence of RBD (at the initial encounter); family history of PD, tremor, dementia; tremor-predominant subtype at initial presentation; and akinetic/rigid subtype at initial presentation. Since cohort sizes, demographic and clinical characteristics (Table 1), and outcomes (Supplementary Table S12) varied by study site, and genetic background may differ by study site, we also report results for trajectory models where study site, weighted by cohort size, was used as an additional predictor (covariate). Compared to models where study site was weighted by cohort size, models “dummy coding” each study site and evaluating it relative to the largest cohort, DodoNA gave identical trajectories, trajectory groups, and for predictors other than individual study sites, effect sizes and *p* values. In the latter models, some study sites were significant covariates due to the differences in cohort size. Years of education was always used as a predictor in these models.

**Table 1:** Demographic and clinical characteristics of the LONG−PD cohort.

Characteristic^a^	Study site					All
	Norwegian University of Science and Technology	ASAN LONG−PD	University of Thessaly	LuxPark	DodoNA	
*N* (% of all)	77 (8.8)	270 (31.0)	13 (1.5)	103 (11.8)	408 (46.8)	871 (100)
Female, *N* (%)	28 (36.4)	138 (51.1)	6 (46.2)	37 (35.9)	131 (32.1)	340 (39.0)
Education−years, median (range)	12 (7–20)	12 (2–22)	6 (4–14)	13 (5–25)	16 (4–24)	14 (2–25)
Age at initial motor symptom onset, median (range)	66 (27–82)	62 (29–103)	67 (38–80)	63 (27–88)	70 (38–94)	66 (27–103)
Years from initial motor symptom onset at initial visit, median (range)	2 (0–5)	1 (0–4)	2 (0–5)	2 (0–5)	2 (0–5)	2 (0–5)
Follow-up interval in years, median (range)	6 (2–10)	5 (1–9)	4 (2–8)	7 (5–10)	6 (1–10)	6 (1–10)
Self-reported pesticide exposure, *N* (%)	3 (3.9)	64 (23.7)	4 (30.8)	69 (67.0)	76 (18.6)	216 (24.8)
Self-reported head injury, *N* (%)	7 (9.1)	22 (8.2)	0 (0)	22 (21.4)	154 (37.8)	205 (23.5)
Diabetes, *N* (%)	1 (1.3)	35 (13.0)	1 (7.7)	11 (10.7)	71 (17.4)	119 (13.7)
REM sleep behavior disorder at initial visit, *N* (%)	2 (2.6)	20 (7.4)	0 (0)	16 (15.5)	14 (3.4)	52 (6.0)
Family history of PD, *N* (%)	19 (24.7)	23 (8.3)	2 (15.4)	33 (32.0)	100 (24.5)	177 (20.3)
Family history of dementia, *N* (%)	4 (5.2)	29 (10.7)	2 (15.4)	29 (28.2)	114 (27.9)	178 (20.4)
Family history of tremor, *N* (%)	9 (11.7)	23 (8.5)	0 (0)	28 (27.2)	58 (14.2)	118 (13.6)
Tremor-predominant subtype (initial visit), *N* (%)	43 (55.8)	48 (17.8)	3 (23.1)	32 (31.1)	108 (26.5)	234 (26.9)
Akinetic/rigid subtype (initial visit), *N* (%)	21 (27.3)	205 (75.9)	7 (53.8)	45 (43.7)	117 (28.7)	395 (45.4)
Mixed subtype (initial visit), *N* (%)	13 (16.9)	17 (6.3)	3 (23.1)	26 (25.2)	183 (44.8)	242 (27.8)

a Except where noted, percent refers to the percent of patients at each site or the percent of all patients.

Eight patients had undergone functional surgery for PD, specifically subthalamic nucleus deep-brain stimulation (DBS), at [median (MD)(range(R)): 7(5–10)] years after motor symptom onset. Since the number of patients with DBS was <1% of the total, and the inclusion of DBS as a time-varying covariate in all group-based trajectory models (GBTMs) was not significant, DBS therapy is not reported in this analysis.

Best-fitting models were identified following an iterative process utilizing a fit-criteria assessment plot (Klijn et al., 2017). Initially, the optimal number of latent trajectories, *k*, was identified based on the fit indices Bayesian Information Criterion (BIC), Aikake Information Criterion (AIC) and model-maximized likelihood. Here, the trajectory variable was fit to the same-order polynomial, typically cubic, with dropout modeled based on the prior two data entries, in models with *k* = 2-to-5. We required the smallest group to have >5% of patients to retain clinical relevance. With this restriction, the optimal *k* was chosen by identifying the model with fit indices, particularly BIC, nearest zero, and for each group, average posterior probability of group assignment ≥0.85 and odds of correct classification ≥5.0. Then, optimal polynomial terms were determined by evaluating the significance of zero-, first-, second- or third-order polynomials in modeling each trajectory. Finally, the significance and effect on model-fit of covariates were evaluated, and polynomial-fit was re-optimized. For each set of trajectories, we report each trajectory’s convergence (number of patients assigned to their final trajectory placement) and misclassification (percent of patients not assigned to their final trajectory placement) over the follow-up period.

### Associations between trajectories and PD outcomes

Kaplan–Meier survival analysis was used to evaluate whether the time from initial motor symptom onset to the first occurrence of one of 12 clinical outcomes differed across each set of trajectory-groups. With the exception of ICD, we restricted these evaluations to clinical outcomes that occurred in at least 5% of all patients: motor fluctuations, dyskinesia, dysphagia, cognitive impairment, psychosis (hallucinations, paranoid ideations, and delusions), RBD (excluding patients with RBD at their initial encounter), persistent freezing, persistent falls, persistent orthostatism, and persistent urinary incontinence. In the LuxPark cohort, ICD was documented in 26% of participants (3.1% of all patients) during the 10-year follow-up reported here, but was documented later at other sites. We chose to analyze persistent freezing, falls, orthostatism, and urinary incontinence, with persistence defined as being present in ≥2 annual follow-ups, as persistent occurrence is more likely to reflect disease progression rather than a treatment effect. For each outcome, Kaplan–Meier survival curves are reported for each set of trajectory groups as well as the χ^2^ and *p* values for a log-rank test evaluating differences across that set of trajectory groups. For log-rank tests significant at *p* < 0.05, we also report pairwise differences between trajectories that remained significant following a Bonferroni correction for multiple tests.

## Results

### Demographic and clinical characteristics

**Figure 1:**
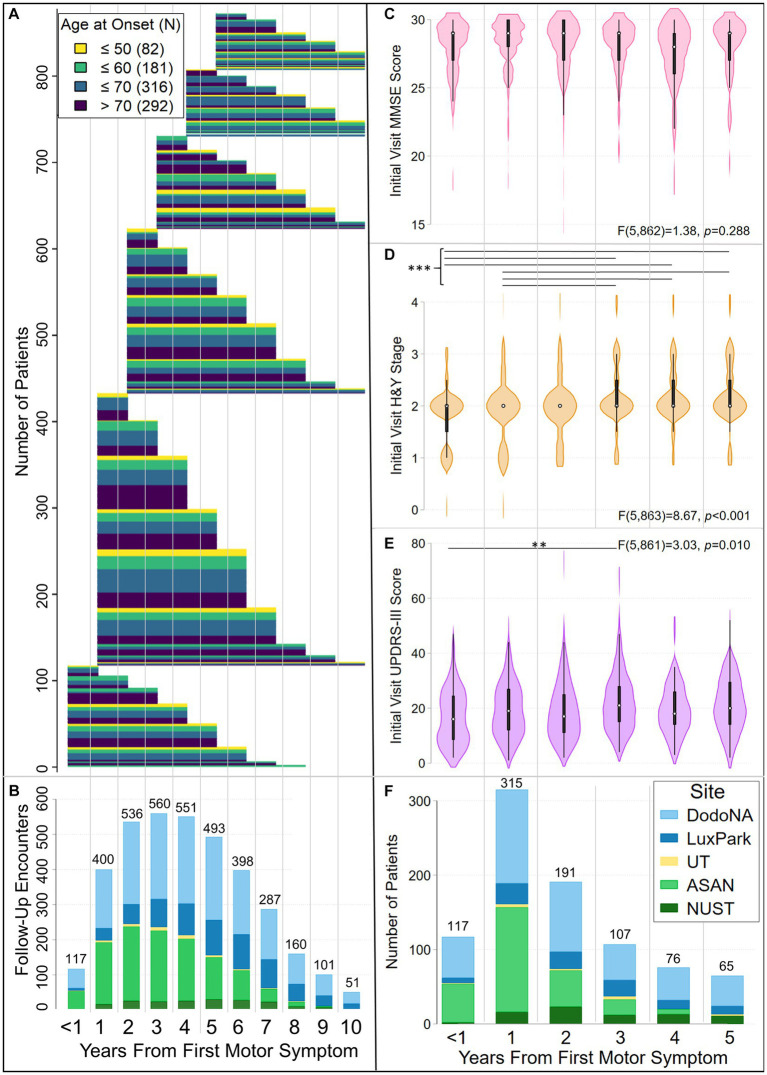
Characteristics of the LONG-PD cohort. Panels **(A)** and **(B)** show follow-up of LONG-PD participants by disease duration (years from initial motor symptom) and age at first motor symptom. Patients assessed in this analysis had motor symptom onset within 5 years of the initial clinical encounter and were followed for up to 10 years after the onset of the initial motor symptom. Panel **(A)** shows the duration of follow-up, color coded by age at onset. Panel **(B)** shows the total number of encounters per year, color coded by site. The <1 coordinate on the *x*-axis, which is jointly used by panels **(A)** and **(B)**, corresponds to initial clinical encounters that occurred within a year of the appearance of the initial motor symptom. To facilitate comparison of data in panels **(A)** and **(B)**, thin gray vertical lines demarcate clinical encounters that occurred in subsequent years. Each patient’s follow-up is depicted by a thin, horizontal line extending from when the initial encounter occurred relative to the initial motor symptom (left end) to the last year of follow-up (right end). Lines are colored by age group at onset (legend top left). Thick bars result from the merged lines of patients in the same age groups who were followed for the same length of time. **(B)** Bar chart illustrating the total number of patient encounters at each year of disease duration, color-coded by site [legend in panel **(F)**]. Violin plots (box plots modified with overlaid plots of the estimated kernel density) show the distribution of **(C)** MMSE scores, **(D)** Hoehn and Yahr (H&Y) stages and **(E)** UPDRS-III scores at the initial clinical encounter, by disease duration. In each violin plot, the white dot identifies the median, the black rectangle the interquartile range, and the spikes extend to the upper- and lower-adjacent values. The results of one-way ANOVAs for MMSE scores, H&Y stages, and UPDRS-III scores by disease duration are noted. Horizontal lines identify differences between disease-duration groups that were significant after Bonferroni correction for multiple testing. Hoehn & Yahr stage differed at the initial clinical encounter between patients with durations of either <1 yr. or 1 yr. and 3-, 4-, or 5- yrs (***p ≤ 0.002). UPDRS-III scores differed at the initial clinical encounter between patients with <1-yr and 3-yr duration (**p = 0.005). Panel **(F)** shows the total number of initial encounters by year from the initial motor symptom, color-coded by site. Panels **(C)**–**(F)** use the same X-axis. Thin gray vertical lines demarcate clinical encounters for each year.

Figure 1 and Table 1 show demographic and clinical characteristics of our cohort. Table 1 also summarizes data on follow-up and variables used as predictors of group membership in trajectory models. Trajectories modeled in this analysis consider data from patients whose initial clinical encounter was within 5 years of their initial motor symptom [MD(R): 2(0–5)] and who were followed for up to 10 years [MD(R): 6(1–10) (Figures 1A,B, Table 1)]. The cohort displayed a wide range of AAO [MD(R): 66(27–103)]. About 10% had an AAO ≤ 50 yr., while nearly 70% had an AAO > 60 yr (Figure 1A). The distribution of patients at study enrollment by year from the initial motor symptom (duration), and their initial-visit UPDRS-III scores, H&Y stage and MMSE scores are shown in Figures 1C–F. As documented there, UPDRS-III score and H&Y stage differed by disease duration at the initial visit. More specifically, UPDRS-III score differed between patients with <1-yr and 3-yr duration (Figure 1E), while H&Y stage differed between patients having either <1 or 1-yr and 3-, 4-, or 5-yr duration (Figure 1D). At the initial visit, 26.9% of the cohort displayed a tremor-predominant subtype, 45.4% an akinetic/rigid subtype, and 27.8% a mixed subtype (Table 1).

### Disease trajectories

With the goal of developing an accurate measure of PD progression that is related to PD outcomes, we first modeled the trajectories of individual assessment measures, and then compared these results to models where multiple assessment measures (UPDRS-III, H&Y, and MMSE) were considered jointly. Model-fit statistics supported the assignment of patients to three groups for both single and multiple assessment measures (Table 2, Supplementary Tables S1–S5). While model-fit criteria for some assessment measures identified a better fit to a greater number of groups, model fits with more than three groups violated the specification that, to retain clinical relevance, all groups must have membership >5%. Similar trajectory patterns and trajectory-group assignments were obtained when longitudinal data from 3-to-9 annual visits (maximum *N* = 675) were used, and when longitudinal data collected through disease-duration year eight, instead of year 10, were used. Compared to models using data through disease-duration year 10, trajectory-group assignments in models using data through disease-duration year eight were different for 4.3% (mean (M) of six models, R: 2.5%–6.2%) of patients.

**Table 2:** Groups identified in trajectory models jointly considering UPDRS-III, Hoehn & Yahr, and MMSE scores.

Group	1 (Reference)	2		3	
*N* (% of 870)	440 (50.5)	247 (28.4)		183 (21.0)	
Fit^a^					
UPDRS-III Hoehn & Yahr MMSE	Linear Linear Linear	Quadratic Linear Quadratic		Linear Linear Linear	
Average posterior probability	0.969	0.935		0.950	
Odds of correct classification^b^	31.20	37.08		69.90	
Observed probability^c^	0.505	0.281		0.213	
**Baseline characteristics associated with trajectory membership** ^d^		**OR [95% CI]** ^d^	***p*** ^d^	**OR [95% CI]** ^d^	***p*** ^d^
Female	−	**0.63 [0.41–0.98]**	**0.042**	1.18 [0.74–1.87]	0.483
Age at motor-symptom onset	−	**0.97 [0.95–0.99]**	**0.004**	**1.10 [1.07–1.13]**	**<0.001**
Years of education	−	**0.83 [0.78–0.88]**	**<0.001**	**0.88 [0.83–0.93]**	**<0.001**
Medical history					
Pesticide exposure	−	**2.52 [1.60–3.96]**	**<0.001**	**1.80 [1.08–3.00]**	**0.022**
Head injury	−	**0.49 [0.28–0.85]**	**0.012**	1.20 [0.73–1.95]	0.456
Diabetes	−	1.12 [0.61–2.06]	0.707	1.75 [0.97–3.18]	0.062
REM sleep behavior disorder	−	1.93 [0.90–4.13]	0.091	0.96 [0.33–2.76]	0.947
Family history					
Parkinson’s disease	−	1.03 [0.62–1.72]	0.882	1.21 [0.71–2.07]	0.467
Dementia	−	0.79 [0.47–1.30]	0.361	0.63 [0.37–1.09]	0.105
Tremor	−	1.33 [0.75–2.35]	0.326	0.91 [0.46–1.82]	0.807
Initial presentation					
Tremor-predominant	−	0.95 [0.52–1.73]	0.871	**0.29 [0.16–0.54]**	**<0.001**
Akinetic/rigid predominant	−	**3.21 [1.86–5.51]**	**<0.001**	1.05 [0.63–1.75]	0.835

OR, odds ratio; 95% CI, 95% confidence interval. OR, 95% CI, and *p* values are in bold if *p* < 0.05.

a Modeled using a censored normal probability distribution for the dependent variable.

b Based on the weighted posterior probability.

c Group probability based on the posterior probabilities.

d Compared to membership in reference trajectory, all models are censored normal.

### Single-variable trajectory groups

In each motor (UPDRS-III total score, UPDRS-III tremor subscore, UPDRS-III bradykinesia-rigidity subscore, H&Y stage) and cognitive (MMSE score) GBTM, three trajectory-groups differed in disease severity and rate of progression (Supplementary Figures S1, S2). The trajectory of Group 1 was more benign, that of Group 2 was intermediate, and that of Group 3 was both more severe and more rapidly progressing. All but one of the trajectories converged (i.e., patients were assigned to their final trajectories) with <5% misclassification (i.e., <5% of patients reassigned to a different final trajectory) by 5 years after the initial motor symptom; the intermediate UPDRS-III score trajectory reached this point at 6 years. Very similar trajectories were identified when *study site* and *years of education* (for motor scores) were added as predictors (*cf.* Supplementary Figures S1–S17 and S2–S18). The trajectory-group assignments in the models with these additional predictors were different for 4.6% (M of five models, R: 2.1%–8.7%) of patients.

For each objective assessment, predictors of Group 2 or Group 3 membership relative to Group 1 (= reference group), which followed the more benign trajectory, were modeled jointly with the trajectories (Supplementary Tables S1–S5). Individuals with the tremor-predominant subtype at initial presentation were more likely to be in Group 1 for UPDRS-III total score, UPDRS-III bradykinesia-rigidity subscore, and H&Y stage, but not more likely to be in any Group for UPDRS-III tremor-predominant subscore. In contrast, individuals with the akinetic/rigid subtype at initial presentation were more likely to be in Group 2 or 3 for UPDRS-III total score and Group 3 for UPDRS-III bradykinesia-rigidity subscore, but Group 1 for UPDRS-III tremor subscore, as tremor is not a manifestation of the akinetic/rigid subtype.

Sex was predictive only for MMSE-score trajectory membership, with females more likely to be in Group 1. Older AAO was associated with membership in Group 2 or 3 for H&Y stage and for MMSE-score, and with membership in Group 3 for UPDRS-III bradykinesia-rigidity subscore. Individuals with fewer years of education were more likely to be in Group 2, and even more likely to be in Group 3, for MMSE score.

Individuals with self-reported pesticide exposure were more likely to be in Group 2, and even more likely to be in Group 3, for UPDRS-III total score. Individuals with self-reported prior head injury were more likely to be in Group 1 for UPDRS-III-total score, but more likely to be in Group 2 or 3 for MMSE score. Individuals with diabetes were more likely to be in Group 3 for UPDRS-III-total score, UPDRS-III-tremor subscore, and H&Y stage, and in Group 2 or 3 for MMSE score. Individuals with RBD at their initial encounter were more likely to be in Group 3 for UPDRS-III-total score. Interestingly, individuals with a family history of dementia were more likely to be in Group 1 for UPDRS-III-total score.

When *study site* and *years of education* (for motor-score models) were added as covariates in these trajectory models, several important predictors of group membership in models without these covariates were retained (*cf.* Supplementary Tables S1–S5 to S6–S10, respectively). In both types of models, older age at motor-symptom onset was associated with membership in Group 3 for UPDRS-III bradykinesia-rigidity subscore, and in Groups 2 or 3 for H&Y and MMSE scores. A tremor-predominant initial presentation was less likely in Group 2 for UPDRS-III score and in Groups 2 or 3 for UPDRS-III bradykinesia-rigidity subscore and H&Y stage. Patients living with diabetes were more likely to be in Group 3 for UPDRS-III, UPDRS-III tremor subscore, and H&Y stage and in Group 2 for MMSE scores. Females were less likely to be in Group 3 for MMSE score.

Some associations gained significance when *study site* and *years of education* were included. Older age at motor-symptom onset was associated with membership in Group 2 or 3 for UPDRS-III score and in Group 2 for UPDRS-III bradykinesia-rigidity subscore. A tremor-predominant subtype at initial presentation was less likely to be in Group 3 for UPDRS-III score. Patients living with diabetes were more likely to be in Group 2 for H&Y stage. Females were less likely to be in Group 3 for UPDRS-III and UPDRS-III bradykinesia-rigidity subscores. Fewer years of education were associated with membership in Group 3 for UPDRS-III score. Head injury was associated with Group 2 and 3 UPDRS-III tremor subscores.

Some associations lost significance when *study site* and *years of education* were included. Age at motor-symptom onset was not associated with membership in Group 2 for UPDRS-III tremor-subscore. Patients living with diabetes were not more likely to be in Group 3 for MMSE score. Head injury was not associated with Group 2 or 3 MMSE or UPDRS-III scores. Pesticide exposure was not associated with UPDRS-III Group 2 or 3. Family history of PD, dementia, or tremor were not associated with any trajectory group. An akinetic/rigid predominant subtype at initial presentation was not associated with Group 2 or 3 for UPDRS-III score or Group 3 for UPDRS-III bradykinesia-rigidity subscore.

### Multivariable (UPDRS-III score, Hoehn & Yahr Stage, MMSE score) trajectories

**Figure 2:**
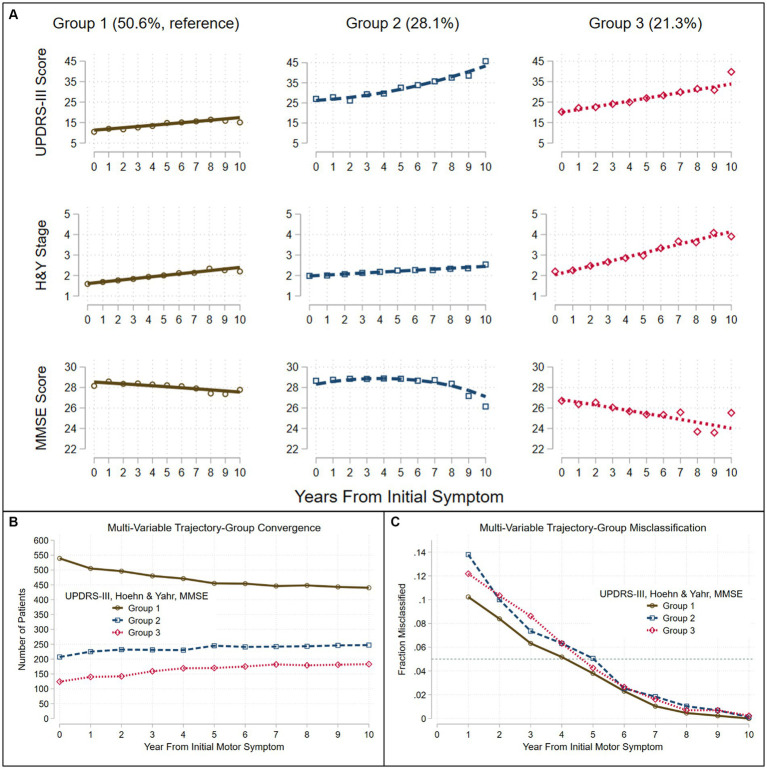
Trajectories seen in the LONG-PD cohort when three assessments are modeled jointly. Group-based trajectory modeling using UPDRS-III score, Hoehn and Yahr (H&Y) Stage and Mini-Mental Status Exam (MMSE) score identified three groups **(A)**. Assignment to group-membership trajectories converge **(B)** with <5% misclassification **(C)** (dashed teal line) by about 5 years after the onset of the initial motor symptom. Trajectories were modeled jointly with the predictors: sex, age at motor-symptom onset, education-years, pesticide exposure, head injury, diabetes, REM-behavior sleep disorder, family history (Parkinson’s disease, dementia, or tremor), and initial presentation (tremor-predominant, akinetic/rigid predominant).

The trajectories observed in the three groups identified when three variables (UPDRS-III total score, H&Y stage, MMSE score) were simultaneously modeled, generally followed the patterns identified in single-variable trajectories (Figure 2A). The trajectories modeled for Group 1 (50.6%), showed relatively benign progression in all three measures. Compared to Group 1, Group 3 (21.3%) showed a much faster rate of progression of motor dysfunction, disease stage, and cognitive decline. In contrast to Groups 1 and 3, Group 2 (28.1%) showed a faster rate of progression of both UPDRS-III and MMSE scores after year five. Group membership appeared stable with <5% likelihood of misclassification 5 years from motor symptom onset (Figures 2B,C).

Predictors of Group 2 or 3 membership relative to the more benign Group 1 (=reference group) are shown in Table 2. Individuals with the tremor-predominant subtype at initial presentation were less likely to be in Group 3, whereas individuals with the akinetic/rigid subtype at initial presentation were more likely to be in Group 2.

Females were less likely to be in Group 2 than in Group 1. Older AAO increased the likelihood for membership in Group 3, but decreased the likelihood for membership in Group 2. Individuals with fewer years of education were more likely to be in Group 2 or 3.

Individuals with self-reported pesticide exposure were more likely to be in Group 2 or 3; however, individuals with self-reported prior head injury were less likely to be in Group 2.

When *study site* was added as a covariate in this trajectory model, the trajectories remained very similar, and each trajectory group included a similar percentage of patients (*cf.* Table 2 to Supplementary Table S11; Figure 2 to Supplementary Figure S14). Three important characteristics – age at motor-symptom onset, years of education, and tremor-predominant disease subtype—remained significantly associated with group membership. Older age at motor-symptom onset was associated with both Groups 2 and 3, fewer years of education was associated with Group 3, and a tremor-predominant subtype at initial presentation was less likely in Group 3. However, sex, pesticide exposure, head injury, and akinetic/rigid predominant subtype were not associated with group membership.

### Association of disease outcomes with single and multivariable trajectories

**Figure 3:**
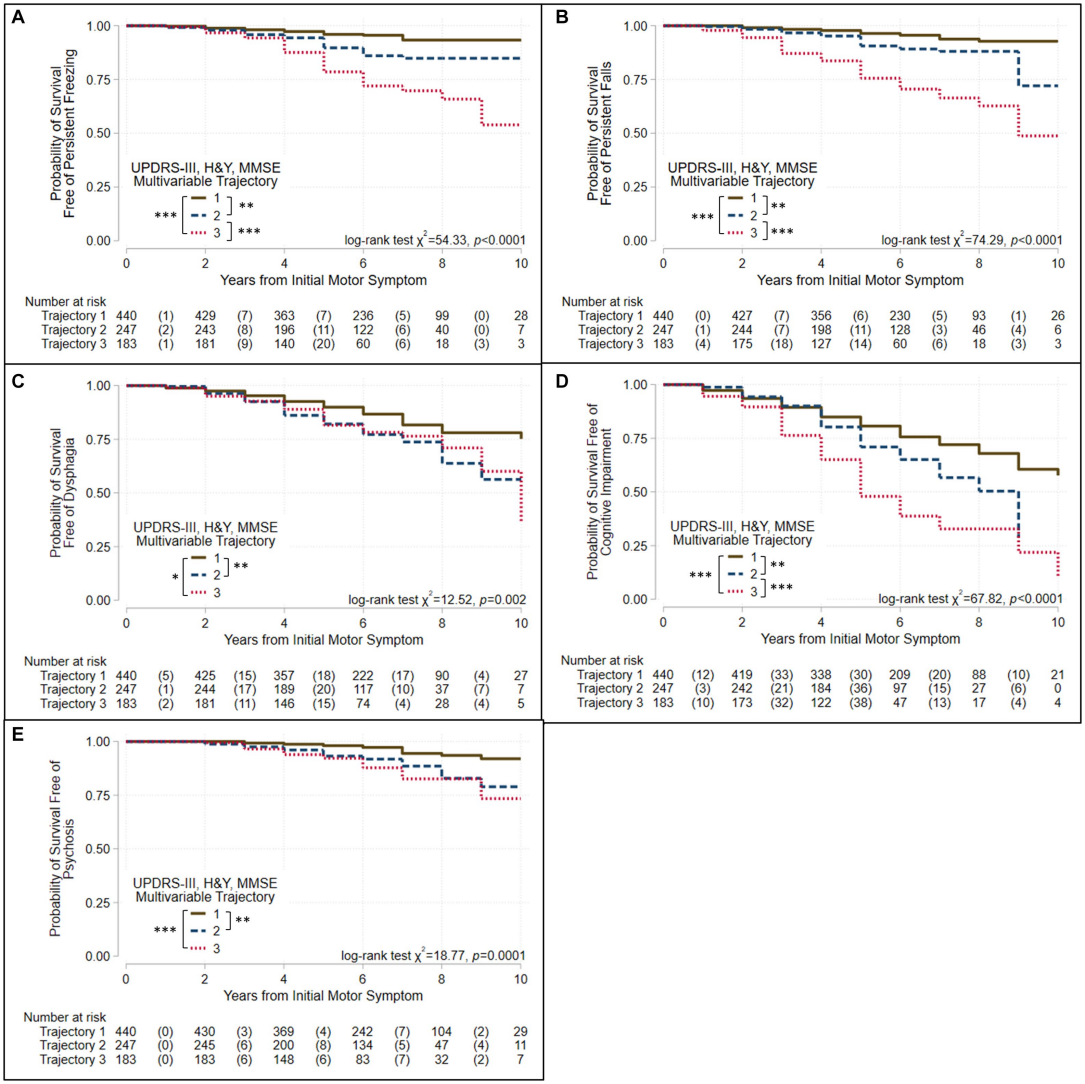
Survival free of clinically significant milestones in the groups identified when three assessments are modeled jointly. Kaplan–Meier analyses for survival free of **(A)** persistent freezing, **(B)** persistent falls, **(C)** dysphagia, **(D)** cognitive impairment, and **(E)** psychosis in trajectory-groups identified using group-based trajectory models simultaneously considering UPDRS-III total score, Hoehn & Yahr (H&Y) stage, and Mini-Mental Status Exam (MMSE) score. Trajectories were modeled jointly with the predictors: sex, age at motor-symptom onset, education-years, pesticide exposure, head injury, diabetes, REM-behavior sleep disorder, family history (Parkinson’s disease, dementia, or tremor), and initial presentation (tremor-predominant, akinetic/rigid predominant). The at-risk table beneath each plot shows the number at-risk at each time point, with the number of failed (outcome reached) events listed in parentheses. Log-rank test results are shown. Asterisks identify pairs of trajectory-groups where outcomes differ in pairwise log-rank tests with a Bonferroni-corrected *p* < 0.05 (*), *p* < 0.01 (**), or *p* < 0.001 (***). The outcomes of persistent freezing, persistent falls, and cognitive impairment are poorest in Group 3, which show the most severe trajectories, and less poor in Group 2, which show intermediate trajectories, compared to Group 1, which shows trajectories that are more benign. The outcomes of dysphagia and psychosis are similar in Groups 2 and 3, but poorer than in Group 1.

**Figure 4:**
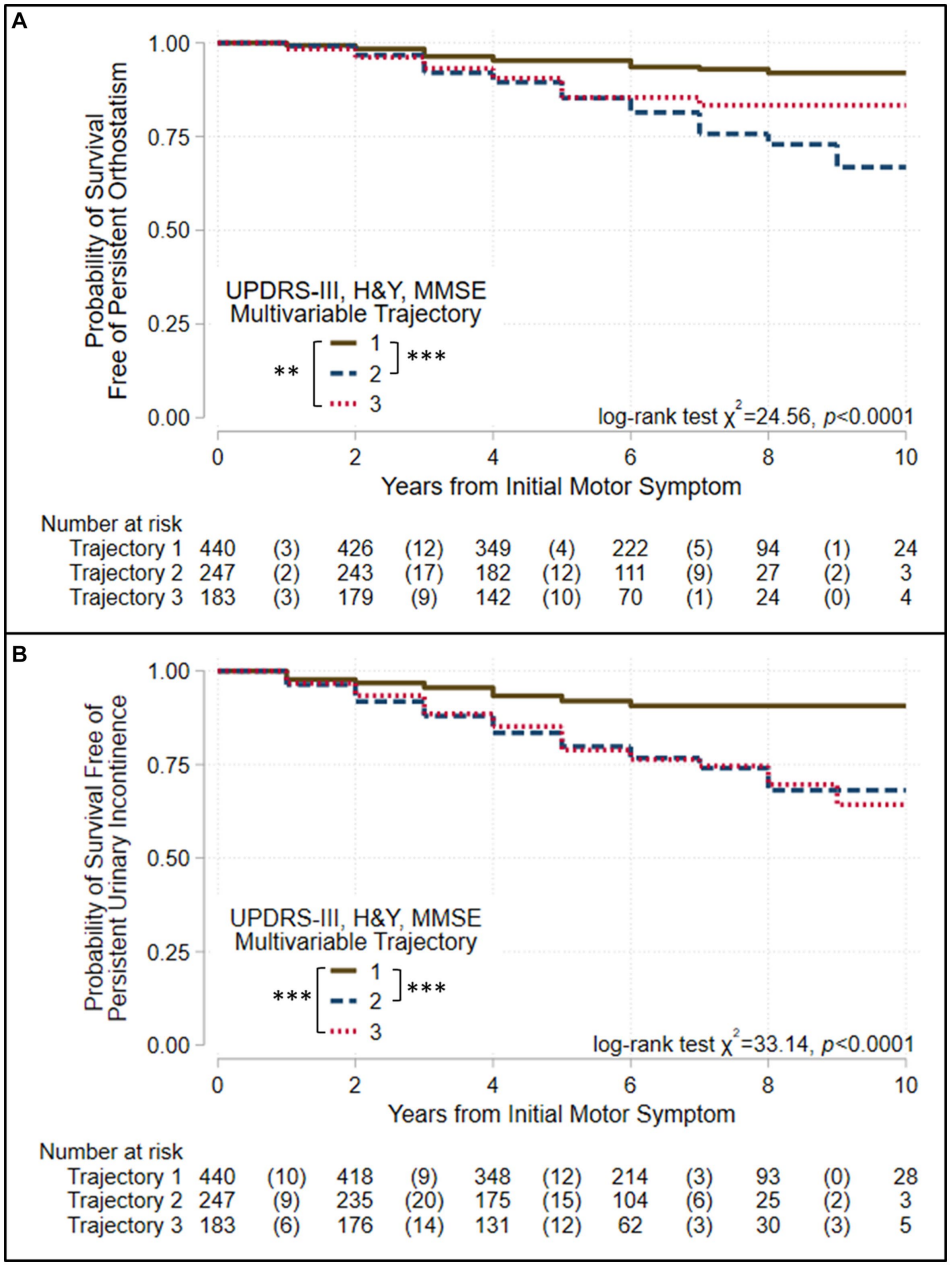
Survival free of autonomic symptoms in the groups identified when three assessments are modeled jointly. Kaplan–Meier analyses for survival free of **(A)** persistent orthostatism and **(B)** persistent urinary incontinence in trajectory-groups identified using group-based trajectory models simultaneously considering UPDRS-III total score, Hoehn & Yahr (H&Y) stage, and Mini-Mental Status Exam (MMSE) score. Trajectories were modeled jointly with the predictors: sex, age at motor-symptom onset, education-years, pesticide exposure, head injury, diabetes, REM-behavior sleep disorder, family history (Parkinson’s disease, dementia, or tremor), and initial presentation (tremor-predominant, akinetic/rigid predominant). The at-risk table beneath each plot shows the number at-risk at each time point, with the number of failed (outcome reached) events listed in parentheses. Log-rank test results are shown. Asterisks identify pairs of trajectory-groups where outcomes differ in pairwise log-rank tests with a Bonferroni-corrected *p* < 0.05 (*), *p* < 0.01 (**), or *p* < 0.001 (***). All outcomes are similar in Groups 2 and 3, which show intermediate and severe trajectories, respectively, but poorer than Group 1, which shows trajectories that are more benign.

We used Kaplan–Meier survival analyses to evaluate whether survival free of each of 11 PD outcomes differed significantly across the three groups defined by each trajectory analysis. If a log-rank test identified differences in an outcome across the three groups, pairwise group differences were evaluated using Bonferroni correction for multiple tests. Figures 3, 4 and Supplementary Figures S3–S13 show the results of these analyses. Three patterns of outcome-free survival were common in both single and multivariable trajectory groups. In the first, the outcome was poorest in Group 3 (severe disease trajectory), less poor in Group 2 (intermediate trajectory) and least poor in Group 1 (most benign trajectory). In the second, the outcome was similar in Groups 2 and 3 but poorer than in Group 1. In the third, the outcome was poorest in Group 3, and similar but less poor in Groups 1 and 2. Table 3 summarizes the patterns observed for each outcome across the groups defined by the trajectory analyses.

**Table 3:** Patterns of significant differences in the survival free of an outcome across trajectory groups.^a^

Outcome	Assessment used in group-based-trajectory model						Figures
	UPDRS-III score	UPDRS-III tremor subscore	UPDRS-III Bradykinesia-rigidity subscore	Hoehn & Yahr stage	MMSE score	UPDRS-III, Hoehn & Yahr, MMSE multivariable	
Motor fluctuations	ns	ns	ns	[3] < [1]	ns	ns	S3
Dyskinesia	ns	[1] < [3]	ns	ns	ns	ns	S4
Persistent freezing	[3] < [2] < [1]	ns	[2, 3] < [1]	[3] < [1,2]	[3] < [1]	[3] < [2] < [1]	3, S5
Persistent falls	[3] < [1,2]	ns	[2,3] < [1]	[3] < [2] < [1]	[2,3] < [1]	[3] < [2] < [1]	3, S6
Persistent orthostatism	[3] < [2] < [1]	ns	ns	ns	ns	[2,3] < [1]	4, S7
Persistent urinary incontinence	[3] < [1,2]	ns	ns	ns	[3] < [1]	[2,3] < [1]	4, S8
Dysphagia	[3] < [1,2]	ns	ns	[3] < [1,2]	ns	[2,3] < [1]	3, S9
REM sleep behavior disorder ^b^	[2,3] < [1]	ns	[3] < [1]	ns	ns	[2] < [1,3]	S10
Cognitive impairment	[3] < [2] < [1]	ns	[3] < [1,2]	[3] < [2] < [1]	[3] < [2] < [1]	[3] < [2] < [1]	3, S11
Psychosis	[3] < [1,2]	ns	ns	[3] < [1]	[3] < [1]	[2,3] < [1]	3, S12
Impulse control disorder	[3] < [1,2]	nd	nd	[1] < [2]	ns	[2] < [1,3]	S13

UPDRS-III, Unified Parkinson’s Disease Rating Scale part III (motor); MMSE, Mini-Mental Status Exam; ns, not significant, either a log-rank test failed to identify significant differences in survival free of the outcome across the three trajectories or pairwise differences were not significant following Bonferroni correction for multiple tests; nd, not done, none of the patients with available UPDRS-III tremor subscore and bradykinesia-rigidity subscore data developed impulse control disorder.

a If a log-rank test revealed a significant difference between an outcome across a set of three trajectory groups, pairwise log-rank tests were used to assess differences between pairs of trajectory groups. The table reports trajectory groups that show significant differences for an outcome after Bonferroni correction for multiple tests. A bracketed number refers to a trajectory group for an assessment. A trajectory group to the left of the “<” sign showed poorer survival free of the outcome than a trajectory group to the right. In some cases, no pairs or only one pair of trajectory groups showed differences that remained significant following Bonferroni correction. Compare the Kaplan–Meier survival probability plotted in the indicated figure.

b Patients with REM sleep behavior disorder at the initial visit were excluded from these analyses.

While motor fluctuations and dyskinesias were complications of therapy that occurred in more than 12% of our patients (Supplementary Table S6), survival free of these outcomes varied relatively little across the groups identified by either single or multivariable trajectory analyses. Outcomes for motor fluctuations were poorer in Group 3 than Group 1 for H&Y stage (Supplementary Figure S3). In contrast, outcomes for dyskinesias were poorer for UPDRS-III-tremor-subscore in Group 1 than in Group 3 (Supplementary Figure S4).

All outcomes other than complications of levodopa therapy varied across the groups defined by the multivariable and the UPDRS-III-total-score trajectory models (Table 3, Figures 3, 4, Supplementary Figures S5–S13). However, they did not always vary across the groups defined by other single-variable trajectory models (e.g., H&Y-stage or MMSE-score GBTMs). Across the three groups defined in the multivariable trajectory model, the outcomes of persistent freezing (9.9% of patients, Supplementary Table S6), persistent falls (10.3%), and cognitive impairment (32.8%) was poorest in Group 3, less poor in Group 2, and least poor in Group 1 (Figure 3). That is, the severity of these outcomes paralleled the severity of disease course in the groups defined by the multivariable trajectory model. In contrast, the outcomes of dysphagia (17.6%) and psychosis (6.9%) in Groups 2 and 3 were similar but poorer than in Group 1 (Figure 3). This pattern was also seen for autonomic dysfunction outcomes: persistent orthostatism (10.3%), and persistent urinary incontinence (14.2%) also had similar outcomes in Groups 2 and 3 that were poorer than in Group 1 (Figure 4). The difference in outcomes for axial symptoms such as persistent freezing of gait and falls, and outcomes such as orthostatism and urinary incontinence may reflect response to treatment. While axial symptoms are treatment-resistant, other features are treatment-responsive at least for part of the disease course. A different pattern was seen for the outcomes of RBD (33.9%, analysis restricted to patients without RBD at the initial encounter) and ICD (3.1%). These outcomes were poorer in Group 2 than in Groups 1 and 3 (Supplementary Figures S10, S13).

Very similar patterns of disease outcomes across a set of trajectory groups were seen when trajectory groups were modeled together with the additional predictors *study site* and *years-of-education* (*cf.* Table 3 to Supplementary Table S13, Figures 3, 4, and Supplementary Figures S3–S13 to Supplementary Figures S15, S16, and S19–S29, respectively). When differences in these patterns were seen, they usually reflected whether a single intergroup difference gained or lost significance following a Bonferroni correction for multiple tests. It is notable that Group 3 remains distinct in that it is associated with the poorest outcomes regardless of whether *study site* and *years of education* were included as covariates.

The groups defined by the multivariable trajectory model better delineated disease outcomes than did the groups defined by the UPDRS-III-total-score trajectory model, even though outcomes in the groups defined by both models share similarities (Table 3, Figures 3, 4, Supplementary Figures S5–S13). More specifically, the benign disease group defined by the multivariable trajectory model showed better outcomes than the intermediate/severe disease groups for persistent falls, persistent urinary incontinence, dysphagia, and psychosis. These outcomes are similar in the benign and intermediate disease groups defined by the UPDRS-III-total-score trajectory model. Hence, outcomes that capture milestones in disease progression and have a significant effect on disease prognosis and quality of life, such as freezing, falls, autonomic dysfunction, cognitive impairment, and psychosis, were more consistently and accurately reflected in the multivariable trajectory analysis.

## Discussion

We present an analysis of disease trajectories and clinical outcomes in a longitudinal study of a large multiethnic, multisite PD cohort. We used GBTM with UPDRS part III, H&Y stage, and MMSE assessments, individually and in combination, to identify groups that follow one of three trajectories with differential severity and rates of progression. In general, patients show either a benign, slowly progressive disease course, an intermediate, intermediately progressive course, or a severe, more rapidly progressive course. Assignment to a group following a specific trajectory remained stable and misclassification was low 4 to 5 years after the initial motor symptom. This indicates that long-term follow-up of at least this duration is required to accurately determine disease course and prognosis. The groups defined by the multivariable trajectory model better delineate disease course and outcomes. Thus, this modeling approach provides a clinically useful framework to consistently identify rates of disease progression and severity using a combination of objective measures rather than subjective assessment measures. These findings support the hypothesis that disease classification should not depend only on the presence of a single or a combination of symptoms, but rather include longitudinal trajectories that capture the rate of disease progression by taking advantage of standardized objective assessments that capture the full phenotypic spectrum of PD (Puschmann et al., 2015).

In the multivariable trajectory model, relative to Group 1 (more benign trajectories) membership, predictors of intermediate-group membership were akinetic/rigid subtype at initial presentation, fewer years of education, younger AAO, and pesticide exposure. Individuals were less likely to be in the intermediate group if they had reported a head injury or were female. Predictors of severe group membership were older AAO, fewer years of education, and pesticide exposure. Individuals were less likely to be in the severe group if they initially presented with a tremor-predominant subtype. Interestingly, a tremor-predominant initial presentation is not a good predictor of any UPDRS-III-tremor-subscore trajectory-group, but is a useful predictor of an absence of membership in the intermediate and/or severe trajectory-groups for UPDRS-III, UPDRS-III bradykinesia-rigidity subscore, H&Y stage, and the multivariable trajectories. This suggests that the initial presence or absence of tremor is a less reliable indicator of later disease severity or progression.

In addition, specific outcomes that reflect clinically significant milestones of disease progression, such as persistent freezing of gait, persistent falls, orthostatism and development of complications of levodopa therapy, were differentially associated with trajectory groups. As a rule, these outcomes tended to occur earliest and most frequently in the group following the most severe trajectory. The development of some important clinical outcomes, such as freezing, falls, autonomic dysfunction, dysphagia, psychosis, and cognitive impairment, showed patterns that were most consistently found in the groups identified when GBTM jointly assessed the trajectories of UPDRS-III, H&Y, and MMSE scores. In one pattern, an outcome was poorest in Group 3 (severe trajectory), less poor in Group 2 (intermediate trajectory) and least poor in Group 1 (most benign trajectory). In a second, an outcome was poorest in Group 3, with Groups 2 and 1 showing less poor but similar outcomes. In a third, the outcome was poorest but similar in Groups 2 and 3, and less poor in Group 1. In general, treatment-resistant axial symptoms, such as persistent freezing and falls, occurred earlier and more frequently in the group following the severe trajectory, whereas features that may be treatment-responsive, at least for part of the disease course, occurred later and less frequently in the groups having more intermediate or benign trajectories.

Dysphagia is a significant late manifestation of bradykinesia and often co-occurs with hypokinetic dysarthria as the disease progresses. During the follow-up period, dysphagia was noted in 17.6% of patients, while dysarthria was noted in 3.3% of patients. Since we presented analyses of outcomes only if they appeared in >5% of patients, we did not present analyses of dysarthria in the results. The fact that dysarthria was recorded in <5% of patients may reflect lack of data entry for this symptom at some sites, or that was a later manifestation, not appearing in many patients during the assessment period at some sites. In addition, the severity of hypophonia was assessed by the UPDRS-III total and UPDRS-III bradykinesia-rigidity subscores, whereas the presence, but not the severity of dysphagia was assessed at successive annual evaluations. Indeed, in a Kaplan–Meier analysis to evaluate how dysarthria varied across trajectory groups (that were modeled with *study site*), dysarthria, like dysphagia, had a poorer outcome in UPDRS-III score Group 3 than either Groups 1 or 2, where it was similar (overall *p* = 0.011, Bonferroni corrected *p* < 0.05 for Group 3 vs. 1 and 3 vs. 2). While dysarthria did not vary across other sets of trajectory groups, only 1.2% (8/678) of the patients available for modeling UPDRS-III bradykinesia-rigidity subscore trajectories developed dysarthria during the follow-up period. A possible source of variability in the development of dysphagia and dysarthria are behavioral treatments. These include speech therapy or evaluation by video swallowing, which have been obtained in some patients, but were not included in the analysis as predictors of trajectory-group assignment.

Interestingly, the most benign tremor-subscore trajectory was associated with poorer outcomes for dyskinesias. This difference could reflect differences in the underlying neurodegenerative process and/or treatment response.

In agreement with our previous study, the tremor-predominant subtype as ascertained at the initial visit was associated with the benign UPDRS-III, H&Y stage, and multivariable trajectories, whether or not study site was included in modeling the trajectory groups. The tremor-dominant subtype has been previously described as being distinct from the akinetic/rigid form of the disease (Lawton et al., 2018). Taken together, these findings suggest that the tremor-predominant disease subtype reflects a different underlying neurodegenerative process than the akinetic/rigid subtype.

Genetic factors clearly contribute to disease progression and the severity of specific disease characteristics when these are assessed relative to age and disease duration. For example, common genetic variation at *GBA1* and *APOE* affects the rate of cognitive decline (Szwedo et al., 2022). *GBA1* mutations are associated with earlier age of onset, greater disease severity and motor subtype (Malek et al., 2018), a disease subtype with weaker levodopa response and poorer prognosis (Zhou et al., 2023), and some *GBA1* mutations are associated with reduced survival and more rapid progression (Brockmann et al., 2014; Celia et al., 2016). Large genome-wide association studies (GWAS) have also provided insight into the genetic landscape influencing disease progression and severity. Liu et al. (2021), in a study of 3,821 patients, identified three novel loci associated with cognitive progression in PD, in addition to confirming associations with *GBA1* and *APOE.* Tan et al. (2022), in an analysis of 11 cohorts from Europe and the Americas, including the PPMI, Tracking Parkinson’s, and Oxford Discovery cohorts (Lawton et al., 2022), identified six novel loci associated with PD motor progression or mortality, and found that the E326K *GBA1* variant was associated with increased mortality. Martínez Carrasco et al. (2023), in a GWAS meta-analysis, identified an association between axial motor progression and expression of *ACP6* that suggests mitochondrial lipid homeostasis plays a role in motor progression. Hence, the genetic architecture underlying motor or cognitive progression in PD appears to be somewhat separate from that for disease susceptibility. For these reasons, it will be important to elucidate the relationship between different disease subtypes and the underlying differential spatial dissemination of key pathological proteins (e.g., α-synuclein), genetic factors that influence disease course and outcomes, and environmental exposures. Longitudinal analyses of multiple cohorts are essential to address the role of genetic architecture in disease progression and outcomes. Phenotypic and genetic variability raises the significance of more accurate disease characterization and biological staging (Chahine et al., 2023; Höglinger et al., 2023).

Fewer years of education was associated with membership in the groups following the intermediate and more severe trajectories in the multivariable and H&Y-stage GBTM models. This supports prior findings suggesting a role for cognitive reserve in the development of motor and cognitive symptoms (Valenzuela et al., 2007; Hindle et al., 2016; Lee et al., 2019).

Impulse control disorders were associated with the most severe UPDRS-III trajectory, but also with the most benign H&Y stage and the intermediate multivariable trajectories. Within the follow-up period analyzed here, ICD was noted only in the LuxPark cohort, possibly reflecting different treatment practices.

Our results are in agreement with those of Bartl et al. (2022), who compared progression indicators in the DeNoPa and PPMI cohorts and found similar slopes of progression in both cohorts and that higher scores at baseline for ADLs, freezing, and rigidity were predictors of faster progression.

While individual cohorts, such as those in the DeNoPa, PPMI, and the TrackingPD studies, have identified individual characteristics that contribute to disease progression, it is important to incorporate these characteristics in a multifactorial mode of phenotypic assessment that may more accurately reflect determinants of disease progression.

Our results may be influenced by differences in sample size, treatment practices at the participating sites, the number of patients seen at each annual interval, the duration of follow-up, and/or minor variations in the instruments used for objective assessment of patients, e.g., the use of UPDRS-III vs. MDS-UPDRS-III and MMSE vs. MoCA vs. STMS, which were allowed in the study protocol.

When *study site* was included in the GTBMs, the trajectories shown by groups of patients, the numbers of patients assigned to each trajectory group, and the outcomes associated with different trajectory groups are very similar to those in GBTMs when *study site* was not included. This supports the important conclusion that the trajectory groups and the outcomes associated with them are relatively robust to site-specific effects. Also robust to site-specific effects are some predictors of trajectory-group membership, such as age at motor-symptom onset, years of education, and tremor-predominant subtype at initial presentation for the groups identified by GTBMs jointly considering UPDRS-III, H&Y and MMSE scores. Interestingly, the partial effects of other predictors, such as akinetic/rigid initial presentation, pesticide exposure, head injury in the aforementioned GBTM, are better captured by site. This would be expected for baseline attributes at sites that vary widely from DodoNA, which was used as the reference. For example, in the aforementioned GBTMs, *pesticide exposure* was a strong predictor of membership in Groups 2 and 3 in the model without *study site* (OR[CI]: 1.6[1.0–2.5], *p* = 0.032 and 4.2[2.5–7.2], *p* < 0.001, respectively), but failed to reach significance predicting membership in Group 3 in the model with *study site* (1.6[0.9–2.7], *p* = 0.099). Correspondingly, the incidence of pesticide exposure was considerably higher at the LuxPark site than the DodoNA site (67.0% vs. 18.6%, respectively). Therefore, it will be important to evaluate additional multiethnic cohorts at different sites to clarify how such predictors contribute to trajectory-group assignment.

While treatment effects were not directly assessed in our study, the development of complications of therapy was mostly similar across groups following different disease trajectories. This supports the hypothesis that treatment effects do not alter the underlying disease process. In addition, since two distinct objective measures that are more (UPDRS-III) or less (H&Y) sensitive to treatment effects have similar directions and rate of disease progression across trajectories, a strong treatment effect on group assignment seems unlikely.

Our study results are in overall agreement with analyses of two Canadian cohorts having a mean follow-up of 4.5 years (Fereshtehnejad et al., 2015) and the PPMI cohort having a mean follow-up of 2.7 years (Fereshtehnejad et al., 2017). These studies identified three disease subtypes using clustering on composite indicators: *mainly motor/slow progression*, *diffuse/malignant*, and *intermediate*. In the Canadian cohorts, patients with the diffuse/malignant phenotype were more likely to have mild cognitive impairment, orthostatic hypotension, and RBD at baseline, and at prospective follow-up showed a more rapid progression in cognition motor signs, motor symptoms and a global composite outcome. In the PPMI cohort, key classifiers were motor summary score, cognitive impairment, RBD and dysautonomia.

Interestingly, in the PPMI cohort, MRI based morphometry of a PD-specific brain network showed more atrophy in the diffuse malignant subtype, compared to the mild motor-predominant subtype and patients with the diffuse malignant subtype progressed with greater decline in cognition and in dopamine functional neuroimaging after an average of 2.7 years. These differences between the subtypes argue in favor of different underlying pathophysiology between the subtypes.

It is also interesting to note that in cohorts with autopsy-confirmed PD (De Pablo-Fernández et al., 2019), age at diagnosis was the only significant variable and that, staging of Lewy pathology and Alzheimer disease–related pathology did not differ between subtypes. This again suggests a contribution of different underlying pathophysiology between disease subtypes.

A review of subtyping studies by Mestre et al. (2021) reported significant methodologic shortcomings, questionable clinical applicability, and unknown biological relevance, and suggested that the clinical and biological signature of PD may be unique to the individual patient. The subtyping studies reported to date differ in their methods of analysis, duration of follow-up, and cohort composition. Despite these differences, similar classification patterns have begun to emerge based on clinical characteristics. Therefore, this review underscores the importance of using a common type of longitudinal analyses in evaluating disease progression. In agreement with this conclusion, based on our study’s results, we argue that it is important to use multifactorial annual objective assessments in large multiethnic cohorts, apply a standard methodology in analyzing the cohorts, such as trajectory analysis, and clinical outcomes (De Roos et al., 2017), and perhaps most importantly, use a long duration of longitudinal follow-up. It is the long duration of follow-up that will allow stable patterns of clinically significant disease progression and severity to emerge.

In that vein, as reviewed by Berg et al. (2021), incorporating prodromal symptomatology and subtypes can inform the symptomatic phase of the disease. Since combinations of prodromal symptoms are also present in the symptomatic phase of the disease, it will be important to assess their effect on disease progression and severity in longitudinal cohorts, as we have begun to do. A combined analysis of the DeNoPa, PPMI and FOUND cohorts led to the development of the PREDIGT score, which was able to identify newly diagnosed PD patients before a motor examination (Li et al., 2022). Variables included in the model were hyposmia, constipation, caffeine intake, metal exposure, head injury, smoking, family history, depression, anxiety and RBD.

In summary, these analyses of a large multiethnic, multisite PD cohort identified three groups of patients that show different trajectories of disease progression based on objective longitudinal assessment, predictors of trajectory-group membership, and different patterns of outcome onset. This work demonstrates the importance of long-term annual follow-up (>5 years) with standardized clinical phenotypic assessment for accurately determining disease course and prognosis. It also supports the hypothesis that disease classification and prognosis are more reliable if longitudinal trajectories that capture the rate of disease progression in multiple phenotypic manifestations are considered. It is important to validate the findings of our study in other longitudinal cohorts using similar analytical methods and thereby determine the robustness of our findings. If indeed this type of analysis for predicting disease progression and outcomes is validated, it can inform clinical practice and the development of therapies that are disease-stage appropriate. In addition, accurate longitudinal phenotypic characterization is essential to inform genomic analyses that can elucidate the underlying neurodegenerative process, leading to targeted therapies that can improve disease outcomes.

## Data availability statement

The raw data supporting the conclusions of this article will be made available by the authors, without undue reservation.

## Ethics statement

The studies involving humans were approved by the Ethical Committee for Central Norway (NUST), the Ethics Committee at the University Hospital of Larissa (UT), the Institutional Review Board of Asan Medical Center (ASAN), the National Research Ethics Committee of the Luxembourg government (LuxPark), and the Institutional Review Board of NorthShore University HealthSystem (DodoNA). The studies were conducted in accordance with the local legislation and institutional requirements. The participants provided their written informed consent.

## Author contributions

KM, JA, RK, SC, RF, DM, and BC contributed to study design and the analysis plan. BC wrote the first draft of the manuscript and performed the statistical analyses. KM, RK, LP, SC, JA, ED, AP, BS, DM, and NK collected clinical data. NA contributed to database management. BC, KM, RK, LP, SC, DM, and RF contributed to manuscript revision. All authors except JA (deceased) approved the manuscript.

## Funding

The Agency for Healthcare Research and Quality (R01HS024057 to DM) and the Auxiliary of NorthShore University HealthSystem provided funding for the initial building of electronic medical record toolkit for the DodoNA Project, which provided the basis for the REDCap database. This study was supported by grants from the Luxembourg National Research Fund (FNR) within the National Centre of Excellence in Research on Parkinson’s disease [NCER-PD; (FNR/NCER13/BM/11264123)] and the PEARL programme (FNR; FNR/P13/6682797 to RK) as well as by the European Union’s Horizon 2020 research and innovation program under Grant Agreement No. 692320 (WIDESPREAD; CENTRE-PD; Grant Agreement No. 692320; CENTRE-PD to RK).

## In memoriam

The authors would like to acknowledge the significant contributions of Jan Aasly, MD, to this study and wish to dedicate this paper to his memory.

## Conflict of interest

The authors declare that the research was conducted in the absence of any commercial or financial relationships that could be construed as a potential conflict of interest.

## Publisher’s note

All claims expressed in this article are solely those of the authors and do not necessarily represent those of their affiliated organizations, or those of the publisher, the editors and the reviewers. Any product that may be evaluated in this article, or claim that may be made by its manufacturer, is not guaranteed or endorsed by the publisher.

## Figures and Tables

**Table:** 

AAO	Age at initial motor symptom onset
ADL	Activities of daily living
AIC	Aikake Information Criterion
ASAN	Asan Medical Center site
BIC	Bayesian information criterion
CI	Confidence interval
CSF	Cerebrospinal fluid
DBS	Deep-brain stimulation
DeNoPa	*De Novo* Parkinson cohort
DodoNA	NorthShore University HealthSystem site
Duration	Years from initial motor symptom onset
GBTM	Group-based trajectory model
GEoPD	Genetic epidemiology of Parkinson’s disease consortium
GWAS	Genome-wide association study
H&Y	Hoehn and Yahr stage
HDL	High-density lipoprotein
ICD	Impulse Control Disorder
LuxPark	Luxembourg Institute of Health and Luxembourg Centre for Systems site
M	Mean
MD	Median
MDS-UPDRS	Movement Disorder Society – Unified Parkinson’s Disease Rating Scale
MMSE	Mini-Mental Status Exam
MoCA	Montreal Cognitive Assessment
MRI	Magnetic resonance imaging
NUST	The Norwegian University of Science and Technology site
OR	Odds ratio
PD	Parkinson’s disease
PIGD	Postural instability gait disorder subtype
PPMI	Primary progression markers initiative
R	Range
RBD	REM (rapid eye movement) sleep behavior disorder
STMS	Short Test of Mental Status
UPDRS-III	Unified Parkinson’s disease rating scale part III (motor)
UT	University Hospital of Larissa site
