# Supplementary material for: Multifactorial assessment of Parkinson’s disease course and outcomes using trajectory modeling in a multiethnic, multisite cohort – extension of the LONG-PD study
Source: Front Aging Neurosci. 2023 Sep 26;15:1240971. doi: 10.3389/fnagi.2023.1240971 (PMC10569724; doi:10.3389/fnagi.2023.1240971)

Multifactorial assessment of Parkinson's disease course and outcomes using trajectory modeling in a multiethnic, multisite cohort – extension of the LONG-PD study

Bruce A Chase, Rejko Krueger, Lukas Pavelka, Sun-Ju Chung, Jan Aasly, Efthimios Dardiotis, Ashvini P. Premkumar, Bernadette Schoneburg, Ninith Kartha, Navamon Aunaetitrakul, Roberta Frigerio, Demetrius Maraganore, Katerina Markopoulou

Supplemental Tables **Page**

Trajectory groups modeled jointly with predictors: sex, age at motor-symptom onset, years of education (only in models based on MMSE scores), pesticide exposure, head injury, diabetes, REM-behavior sleep disorder, family history (Parkinson's disease, dementia, or tremor), and initial presentation (tremor-predominant, akinetic/rigid predominant)

Supplemental Table S1. Groups identified by UPDRS-III (motor) score trajectories 4

Supplemental Table S2. Groups identified by UPDRS-III tremor subscore trajectories 5

Supplemental Table S3. Groups identified by UPDRS-III bradykinesia-rigidity subscore trajectories 6

Supplemental Table S4. Groups identified by Hoehn and Yahr stage trajectories 7

Supplemental Table S5. Groups identified by Mini-Mental Status Exam (MMSE) score trajectories 8

Trajectory groups modeled jointly with additional predictors

Supplemental Table S6. Groups identified modeling UPDRS-III (motor) score trajectories jointly with the additional predictors *years of education* and *study site* 9

Supplemental Table S7. Groups identified modeling UPDRS-III tremor subscore trajectories jointly with the additional predictors *years of education* and *study site* 10

Supplemental Table S8. Groups identified modeling UPDRS-III bradykinesia-rigidity subscore trajectories jointly with the additional predictors *years of education* and *study site* 11

Supplemental Table S9. Groups identified modeling Hoehn and Yahr stage trajectories jointly with the additional predictors *years of education* and *study site* 12

Supplemental Table S10. Groups identified modeling Mini-Mental Status Exam (MMSE) score trajectories jointly with the additional predictor *study site* 13

Supplemental Table S11. Groups identified in trajectory models jointly considering UPDRS-III, Hoehn & Yahr, and MMSE scores and developed with the additional predictor *study site* 14

Parkinson's disease outcomes

Supplemental Table S12. Parkinson's disease outcomes in the LONG-PD cohort 16

Supplemental Table S13. Patterns of significant differences in the survival free of an outcome across trajectory groups modeled with the additional predictors *years of education* and *study site* 17

Supplemental Figures

Trajectories and survival free of Parkinson's disease outcomes when trajectory groups are modeled using predictors: sex, age at motor-symptom onset, years of education (only in models based on MMSE scores), pesticide exposure, head injury, diabetes, REM-behavior sleep disorder, family history (Parkinson's disease, dementia, or tremor), and initial presentation (tremor-predominant, akinetic/rigid predominant)

Supplemental Figure S1. Motor-score trajectories seen in the LONG-PD cohort 18

Supplemental Figure S2. Disease-stage and cognitive trajectories seen in the LONG-PD cohort 19

Supplemental Figure S3. Survival free of motor fluctuations 20

Supplemental Figure S4. Survival free of dyskinesia 21

Supplemental Figure S5. Survival free of persistent freezing 22

Supplemental Figure S6. Survival free of persistent falls 23

Supplemental Figure S7. Survival free of persistent orthostatism 24

Supplemental Figure S8. Survival free of persistent urinary incontinence 25

Supplemental Figure S9. Survival free of dysphagia 26

Supplemental Figure S10. Survival free of REM sleep behavior disorder 27

Supplemental Figure S11. Survival free of cognitive impairment 28

Supplemental Figure S12. Survival free of psychosis 29

Supplemental Figure S13. Survival free of impulse control disorder 30

Trajectories and survival free of Parkinson's disease outcomes when trajectory groups are modeled with additional predictors

Supplemental Figure S14. Trajectories seen in the LONG-PD cohort when trajectories for three assessments are modeled jointly with the additional predictor *study site* 31

Supplemental Figure S15. Survival free of clinically significant milestones in the groups identified when trajectories for three assessments are modeled jointly with the additional predictor *study site* 32

Supplemental Figure S16. Survival free of autonomic symptoms in the groups identified when trajectories for three assessments are modeled jointly with the additional predictor *study site* 33

Supplemental Figure S17. Motor-score trajectories seen in the LONG-PD cohort when trajectories are modeled jointly with the additional predictors *years of education* and *study site* 34

Supplemental Figure S18. Disease-stage and cognitive trajectories seen in the LONG-PD cohort when trajectories are modeled jointly with the additional predictors *years of education* and *study site* 35

Supplemental Figure S19. Survival free of motor fluctuations when trajectories are modeled jointly with the additional predictors *years of education* and *study site* 36

Supplemental Figure S20. Survival free of dyskinesia when trajectories are modeled jointly with the additional predictors *years of education* and *study site* 37

Supplemental Figure S21. Survival free of persistent freezing when trajectories are modeled jointly with the additional predictors *years of education* and *study site* 38

Supplemental Figure S22. Survival free of persistent falls when trajectories are modeled jointly with the additional predictors *years of education* and *study site* 39

Supplemental Figure S23. Survival free of persistent orthostatism when trajectories are modeled jointly with the additional predictors *years of education* and *study site* 40

Supplemental Figure S24. Survival free of persistent urinary incontinence when trajectories are modeled jointly with the additional predictors *years of education* and *study site* 41

Supplemental Figure S25. Survival free of dysphagia when trajectories are modeled jointly with the additional predictors *years of education* and *study site* 42

Supplemental Figure S26. Survival free of REM sleep behavior disorder when trajectories are modeled jointly with the additional predictors *years of education* and *study site* 43

Supplemental Figure S27. Survival free of cognitive impairment when trajectories are modeled jointly with the additional predictor *study site* 44

Supplemental Figure S28. Survival free of psychosis when trajectories are modeled jointly with the additional predictors *years of education* and *study site* 45

Supplemental Figure S29. Survival free of impulse control disorder when trajectories are modeled jointly with the additional predictors *years of education* and *study site* 46

Supplemental Table S1. Groups identified by UPDRS-III (motor score) trajectories

**Group**	**1 (reference)**	**2**		**3**	
N (% of 871)	340 (39.0)	417 (47.9)		114 (13.1)	
Fit [a]	linear	quadratic		linear	
Average posterior probability	0.950	0.930		0.948	
Odds of correct classification [b]	28.96	14.56		117.95	
Observed probability [c]	0.394	0.472		0.134	
**Baseline characteristics associated with trajectory membership** [d]		**OR [95% CI]** [d]	***p*** [d]	**OR [95% CI]** [d]	***p*** [d]
Female	–	nr	nr	nr	nr
Age at motor-symptom onset	–	nr	nr	nr	nr
Medical history					
Pesticide exposure	–	**1.60 [1.04 - 2.46]**	**0.032**	**4.22 [2.47 - 7.18]**	**<0.001**
Head injury	–	**0.60 [0.39 - 0.90]**	**0.016**	**0.51 [0.27 - 0.95]**	**0.036**
Diabetes	–	nr	nr	**1.90 [1.00 - 3.59]**	**0.049**
REM sleep behavior disorder	–	nr	nr	**2.63 [1.12 - 6.19]**	**0.026**
Family history					
Parkinson's disease	–	nr	nr	nr	nr
Dementia	–	**0.49 [0.31 - 0.75]**	**0.001**	0.59 [0.32 - 1.10]	0.098
Tremor	–	nr	nr	nr	nr
Initial presentation					
Tremor-predominant	–	**0.59 [0.37 - 0.94]**	**0.027**	nr	nr
Akinetic/rigid predominant	–	**2.04 [1.32 - 3.16]**	**0.001**	**2.83 [1.48 - 5.42]**	**0.002**

[a] modeled using a censored normal probability distribution for the dependent variable

[b] based on the weighted posterior probability

[c] group probability based on the posterior probabilities

[d] compared to membership in reference trajectory, all models are censored normal. Odds ratios with $p<0.10$ are reported. OR, 95% CI, and *p* values are in bold if $p < 0.05$

Abbreviations – OR, odds ratio; 95% CI, 95% confidence interval; nr, values not reported ($p \geq 0.10$)

Supplemental Table S2. Groups identified by UPDRS-III tremor subscore trajectories

**Group**	**1 (reference)**	**2**		**3**	
N (% of 678)	170 (25.1)	373 (55.0)		135 (19.9)	
Fit [a]	linear	linear		linear	
Average posterior probability	0.934	0.898		0.901	
Odds of correct classification [b]	56.08	12.78		45.08	
Observed probability [c]	0.203	0.408		0.168	
**Baseline characteristics associated with trajectory membership** [d]		**OR [95% CI]** [d]	***p*** [d]	**OR [95% CI]** [d]	***p*** [d]
Female	–	nr	nr	nr	nr
Age at motor-symptom onset	–	**0.97 [0.94 - 0.99]**	**0.034**	0.97 [0.94 - 1.00]	0.081
Medical history					
Pesticide exposure	–	nr	nr	nr	nr
Head injury	–	nr	nr	nr	nr
Diabetes	–	nr	nr	**2.63 [1.06 - 6.48]**	**0.036**
REM sleep behavior disorder	–	nr	nr	nr	nr
Family history					
Parkinson's disease	–	**0.26 [0.11 - 0.61]**	**0.002**	0.43 [0.17 - 1.10]	0.080
Dementia	–	**0.46 [0.22 - 0.95]**	**0.038**	**0.38 [0.16 - 0.91]**	**0.031**
Tremor	–	nr	nr	**4.00 [1.22 - 13.05]**	**0.022**
Initial presentation					
Tremor-predominant	–	nr	nr	14.95 [0.77 - 288.43]	0.073
Akinetic/rigid predominant	–	**0.04 [0.01 - 0.17]**	**<0.001**	**0.06 [0.01 - 0.23]**	**<0.001**

[a] modeled using a censored normal probability distribution for the dependent variable

[b] based on the weighted posterior probability

[c] group probability based on the posterior probabilities

[d] compared to membership in reference trajectory, all models are censored normal. Odds ratios with $p<0.10$ are reported. OR, 95% CI, and p values are in bold if $p < 0.05$

Abbreviations – OR, odds ratio; 95% CI, 95% confidence interval; nr, values not reported ($p \geq 0.10$)

Supplemental Table S3. Groups identified by UPDRS-III bradykinesia-rigidity subscore trajectories

**Group**	**1 (reference)**	**2**		**3**	
N (% of 678)	177 (26.1)	303 (44.7)		198 (29.2)	
Fit [a]	linear	linear		quadratic	
Average posterior probability	0.910	0.886		0.929	
Odds of correct classification [b]	38.41	15.10		43.70	
Observed probability [c]	0.209	0.341		0.229	
**Baseline characteristics associated with trajectory membership** [d]		**OR [95% CI]** [d]	***p*** [d]	**OR [95% CI]** [d]	***p*** [d]
Female	–	nr	nr	nr	nr
Age at motor-symptom onset	–	1.02 [0.99 - 1.04]	0.074	**1.03 [1.00 - 1.06]**	**0.006**
Medical history					
Pesticide exposure	–	nr	nr	nr	nr
Head injury	–	nr	nr	0.59 [0.32 - 1.08]	0.093
Diabetes	–	nr	nr	nr	nr
REM sleep behavior disorder	–	nr	nr	nr	nr
Family history					
Parkinson’s disease	–	**0.36 [0.18 - 0.71]**	**0.003**	nr	nr
Dementia	–	nr	nr	**0.38 [0.20 - 0.72]**	**0.003**
Tremor	–	nr	nr	nr	nr
Initial presentation					
Tremor-predominant	–	**0.20 [0.10 - 0.40]**	**<0.000**	**0.15 [0.07 - 0.33]**	**<0.001**
Akinetic/rigid predominant	–	nr	nr	**2.11 [1.10 - 4.02]**	**0.023**

[a] modeled using a censored normal probability distribution for the dependent variable

[b] based on the weighted posterior probability

[c] group probability based on the posterior probabilities

[d] compared to membership in reference trajectory, all models are censored normal. Odds ratios with $p<0.10$ are reported. OR, 95% CI, and p values are in bold if $p < 0.05$

Abbreviations – OR, odds ratio; 95% CI, 95% confidence interval; nr, values not reported ($p \geq 0.10$)

Supplemental Table S4. Groups identified by Hoehn & Yahr stage trajectories

**Group**	**1 (reference)**	**2**		**3**	
N (% of 871)	147 (16.9)	588 (67.5)		136 (15.6)	
Fit [a]	quadratic	quadratic		linear	
Average posterior probability	0.919	0.957		0.957	
Odds of correct classification [b]	52.12	11.47		116.19	
Observed probability [c]	0.180	0.660		0.160	
**Baseline characteristics associated with trajectory membership** [d]		**OR [95% CI]** [d]	***p*** [d]	**OR [95% CI]** [d]	***p*** [d]
Female	–	nr	nr	nr	nr
Age at motor-symptom onset	–	**1.07 [1.04 - 1.10]**	**<0.001**	**1.14 [1.10 - 1.18]**	**<0.001**
Medical history					
Pesticide exposure	–	nr	nr	nr	nr
Head injury	–	nr	nr	nr	nr
Diabetes	–	2.72 [0.97 - 7.64]	0.057	**3.77 [1.24 - 11.50]**	**0.019**
REM sleep behavior disorder	–	nr	nr	nr	nr
Family history					
Parkinson’s disease	–	nr	nr	nr	nr
Dementia	–	nr	nr	nr	nr
Tremor	–	nr	nr	nr	nr
Initial presentation					
Tremor-predominant	–	**0.39 [0.20 - 0.75]**	**0.005**	**0.08 [0.03 - 0.21]**	**<0.001**
Akinetic/rigid predominant	–	nr	nr	nr	nr

[a] modeled using a censored normal probability distribution for the dependent variable

[b] based on the weighted posterior probability

[c] group probability based on the posterior probabilities

[d] compared to membership in reference trajectory, all models are censored normal. Odds ratios with $p<0.10$ are reported. OR, 95% CI, and p values are in bold if $p < 0.05$

Abbreviations – OR, odds ratio; 95% CI, 95% confidence interval; nr, values not reported ($p \geq 0.10$)

Supplemental Table S5. Groups identified by Mini-Mental Status Exam (MMSE) score trajectories

**Group**	**1 (reference)**	**2**		**3**	
N (% of 870)	378 (43.4)	432 (49.6)		60 (6.9)	
Fit [a]	quadratic	quadratic		linear	
Average posterior probability	0.931	0.930		0.970	
Odds of correct classification [b]	17.65	13.80		414.11	
Observed probability [c]	0.434	0.496		0.069	
**Baseline characteristics associated with trajectory membership** [d]		**OR [95% CI]** [d]	***p*** [d]	**OR [95% CI]** [d]	***p*** [d]
Female	–	**0.62 [0.39 - 0.98]**	**0.044**	**0.38 [0.17 - 0.81]**	**0.013**
Age at motor-symptom onset	–	**1.14 [1.11 - 1.17]**	**<0.001**	**1.23 [1.18 - 1.28]**	**<0.001**
Years of education	–	**0.91 [0.86 - 0.96]**	**0.003**	**0.74 [0.67 - 0.82]**	**<0.001**
Medical history					
Pesticide exposure	–	nr	nr	nr	nr
Head injury	–	**2.00 [1.19 - 3.37]**	**0.009**	**2.83 [1.25 - 6.41]**	**0.012**
Diabetes	–	**2.47 [1.27 - 4.79]**	**0.007**	**2.74 [1.05 - 7.18]**	**0.039**
REM sleep behavior disorder	–	nr	nr	nr	nr
Family history					
Parkinson's disease	–	nr	nr	nr	nr
Dementia	–	nr	nr	2.17 [0.94 - 5.00]	0.069
Tremor	–	nr	nr	nr	nr
Initial presentation					
Tremor-predominant	–	nr	nr	0.41 [0.15 - 1.12]	0.083
Akinetic/rigid predominant	–	nr	nr	nr	nr

[a] modeled using a censored normal probability distribution for the dependent variable

[b] based on the weighted posterior probability

[c] group probability based on the posterior probabilities

[d] compared to membership in reference trajectory, all models are censored normal

[d] compared to membership in reference trajectory, all models are censored normal. Odds ratios with $p<0.10$ are reported. OR, 95% CI, and *p* values are in bold if $p < 0.05$

Abbreviations – OR, odds ratio; 95% CI, 95% confidence interval; nr, values not reported ($p \geq 0.10$)

Supplemental Table S6. Groups identified modeling UPDRS-III (motor) score trajectories jointly with the additional predictors *years of education* and *study site*

**Group**	**1 (reference)**	**2**		**3**	
N (% of 871)	329 (37.8)	430 (49.4)		111 (12.7)	
Fit [a]	linear	quadratic		linear	
Average posterior probability	0.961	0.947		0.965	
Odds of correct classification [b]	39.45	19.24		178.76	
Observed probability [c]	0.383	0.482		0.134	
**Baseline characteristics associated with trajectory membership** [d]		**OR [95% CI]** [d]	***p*** [d]	**OR [95% CI]** [d]	***p*** [d]
Female	−	nr	nr	**0.40 [0.19 - 0.85]**	**0.018**
Age at motor-symptom onset	−	**1.07 [1.04 - 1.10]**	**<0.001**	**1.12 [1.07 - 1.16]**	**<0.001**
Years of education	−	0.93 [0.87 - 1.00]	0.055	**0.88 [0.76 - 0.93]**	**0.001**
Medical history					
Pesticide exposure	−	nr	nr	nr	nr
Head injury	−	nr	nr	nr	nr
Diabetes	−	nr	nr	**3.32 [1.24 - 8.87]**	**0.017**
REM sleep behavior disorder	−	nr	nr	nr	nr
Family history					
Parkinson's disease	−	nr	nr	nr	nr
Dementia	−	nr	nr	nr	nr
Tremor	−	nr	nr	nr	nr
Initial presentation					
Tremor-predominant	−	**0.20 [0.10 - 0.38]**	**<0.001**	**0.12 [0.04 - 0.41]**	**<0.001**
Akinetic/rigid predominant	−	0.56 [0.31 - 1.02]	0.056	nr	nr
Study site (weighted by size)					
DodoNA	−	nr	nr	nr	nr
NUST	−	nr	nr	nr	nr
ASAN	−	nr	nr	nr	nr
UT	−	nr	nr	nr	nr
LuxPark	−	nr	nr	nr	nr

[a] modeled using a censored normal probability distribution for the dependent variable

[b] based on the weighted posterior probability

[c] group probability based on the posterior probabilities

[d] compared to membership in reference trajectory, all models are censored normal. Odds ratios with $p<0.10$ are reported. OR, 95% CI, and p values are in bold if $p < 0.05$

Abbreviations – OR, odds ratio; 95% CI, 95% confidence interval; nr, values not reported ($p \geq 0.10$); NUST, St. Olav's Hospital, The Norwegian University of Science and Technology; ASAN, Asan Medical Center, University of Ulsan College of Medicine; UT, University Hospital of Larissa, University of Thessaly; LuxPark, The Luxembourg Parkinson's Study; DodoNA, The DodoNA project, NorthShore University HealthSystem.

Supplemental Table S7. Groups identified modeling UPDRS-III tremor subscore trajectories jointly with the additional predictors *years of education* and *study site*

**Group**	**1 (reference)**	**2**		**3**	
N (% of 678)	170 (19.5)	376 (41.3)		132 (16.2)	
Fit [a]	linear	quadratic		linear	
Average posterior probability	0.953	0.910		0.902	
Odds of correct classification [b]	79.90	14.50		47.70	
Observed probability [c]	0.203	0.413		0.162	
**Baseline characteristics associated with trajectory membership** [d]		**OR [95% CI]** [d]	***p*** [d]	**OR [95% CI]** [d]	***p*** [d]
Female	−	nr	nr	nr	nr
Age at motor-symptom onset	−	nr	nr	nr	nr
Years of education	−	nr	nr	0.90 [0.80-1.01]	0.086
Medical history					
Pesticide exposure	−	nr	nr	nr	nr
Head injury	−	**2.73 [1.04 - 7.2]**	**0.042**	**3.89 [1.38 – 11.1]**	**0.010**
Diabetes	−	nr	nr	**3.19 [1.04 - 9.84]**	**0.043**
REM sleep behavior disorder	−	nr	nr	nr	nr
Family history					
Parkinson's disease	−	nr	nr	nr	nr
Dementia	−	nr	nr	nr	nr
Tremor	−	nr	nr	2.81 [0.82 - 9.48]	0.096
Initial presentation					
Tremor-predominant	−	nr	nr	nr	nr
Akinetic/rigid predominant	−	**0.01 [0.003 - 0.04]**	**<0.001**	**0.02 [0.004 - 0.06]**	**<0.001**
Study site (weighted by size)					
DodoNA	−	nr	nr	nr	nr
ASAN	−	nr	nr	nr	nr

[a] modeled using a censored normal probability distribution for the dependent variable

[b] based on the weighted posterior probability

[c] group probability based on the posterior probabilities

[d] compared to membership in reference trajectory, all models are censored normal. Odds ratios with $p<0.10$ are reported. OR, 95% CI, and p values are in bold if $p < 0.05$

Abbreviations – OR, odds ratio; 95% CI, 95% confidence interval; nr, values not reported ($p \geq 0.10$); ASAN, Asan Medical Center, University of Ulsan College of Medicine; DodoNA, The DodoNA project, NorthShore University HealthSystem.

Supplemental Table S8. Groups identified modeling UPDRS-III bradykinesia-rigidity subscore trajectories jointly with the additional predictors *years of education* and *study site*

**Group**	**1 (reference)**	**2**		**3**	
N (% of 678)	181 (20.8)	282 (32.4)		215 (24.7)	
Fit [a]	linear	quadratic		linear	
Average posterior probability	0.916	0.901		0.928	
Odds of correct classification [b]	41.67	18.28		41.27	
Observed probability [c]	0.207	0.333		0.238	
**Baseline characteristics associated with trajectory membership** [d]		**OR [95% CI]** [d]	***p*** [d]	**OR [95% CI]** [d]	***p*** [d]
Female	−	nr	nr	**0.36 [0.18 - 0.73]**	**0.004**
Age at motor-symptom onset	−	**1.07 [1.04 - 1.10]**	**<0.001**	**1.10 [1.06 - 1.14]**	**<0.001**
Years of education	−	nr	nr	nr	nr
Medical history					
Pesticide exposure	−	nr	nr	nr	nr
Head injury	−	nr	nr	nr	nr
Diabetes	−	nr	nr	nr	nr
REM sleep behavior disorder	−	nr	nr	nr	nr
Family history					
Parkinson’s disease	−	nr	nr	nr	nr
Dementia	−	nr	nr	nr	nr
Tremor	−	nr	nr	nr	nr
Initial presentation					
Tremor-predominant	−	**0.09 [0.04 - 0.20]**	**<0.001**	**0.06 [0.02 - 0.15]**	**<0.001**
Akinetic/rigid predominant	−	0.52 [0.25 - 1.10]	0.088	nr	nr
Study site (weighted by size)					
DodoNA	−	nr	nr	nr	nr
ASAN	−	nr	nr	nr	nr

[a] modeled using a censored normal probability distribution for the dependent variable

[b] based on the weighted posterior probability

[c] group probability based on the posterior probabilities

[d] compared to membership in reference trajectory, all models are censored normal. Odds ratios with $p<0.10$ are reported. OR, 95% CI, and p values are in bold if $p < 0.05$

Abbreviations – OR, odds ratio; 95% CI, 95% confidence interval; nr, values not reported ($p \geq 0.10$); ASAN, Asan Medical Center, University of Ulsan College of Medicine; DodoNA, The DodoNA project, NorthShore University HealthSystem.

Supplemental Table S9. Groups identified modeling Hoehn & Yahr stage trajectories jointly with the additional predictors *years of education* and *study site*

**Group**	**1 (reference)**	**2**		**3**	
N (% of 871)	160 (18.4)	575 (66.0)		135 (15.5)	
Fit [a]	linear	quadratic		linear	
Average posterior probability	0.917	0.957		0.955	
Odds of correct classification [b]	47.21	12.16		113.85	
Observed probability [c]	0.190	0.650		0.158	
**Baseline characteristics associated with trajectory membership** [d]		**OR [95% CI]** [d]	***p*** [d]	**OR [95% CI]** [d]	***p*** [d]
Female	–	nr	nr	nr	nr
Age at motor-symptom onset	–	**1.08 [1.04 - 1.11]**	**<0.001**	**1.14 [1.10 - 1.18]**	**<0.001**
Years of education	–	nr	nr	nr	nr
Medical history					
Pesticide exposure	–	nr	nr	nr	nr
Head injury	–	nr	nr	nr	nr
Diabetes	–	**2.96 [1.02 - 8.56]**	**0.046**	**3.94 [1.25 - 12.42]**	**0.019**
REM sleep behavior disorder	–	nr	nr	nr	nr
Family history					
Parkinson’s disease	–	nr	nr	nr	nr
Dementia	–	nr	nr	nr	nr
Tremor	–	nr	nr	nr	nr
Initial presentation					
Tremor-predominant	–	**0.40 [0.21 - 0.78]**	**0.007**	**0.09 [0.04 - 0.23]**	**<0.001**
Akinetic/rigid predominant	–	nr	nr	nr	nr
Study site (weighted by size)					
DodoNA	–	nr	nr	nr	nr
NUST	–	nr	nr	nr	nr
ASAN	–	nr	nr	nr	nr
UT	–	nr	nr	nr	nr
LuxPark	–	nr	nr	nr	nr

[a] modeled using a censored normal probability distribution for the dependent variable

[b] based on the weighted posterior probability

[c] group probability based on the posterior probabilities

[d] compared to membership in reference trajectory, all models are censored normal. Odds ratios with $p<0.10$ are reported. OR, 95% CI, and p values are in bold if $p < 0.05$

Abbreviations – OR, odds ratio; 95% CI, 95% confidence interval; nr, values not reported ($p \geq 0.10$); NUST, St. Olav’s Hospital, The Norwegian University of Science and Technology; ASAN, Asan Medical Center, University of Ulsan College of Medicine; UT, University Hospital of Larissa, University of Thessaly; LuxPark, The Luxembourg Parkinson’s Study; DodoNA, The DodoNA project, NorthShore University HealthSystem.

Supplemental Table S10. Groups identified modeling Mini-Mental Status Exam (MMSE) score trajectories jointly with the additional predictor *study site*

**Group**	**1 (reference)**	**2**		**3**	
N (% of 871)	373 (42.9)	436 (50.0)		61 (7.0)	
Fit [a]	linear	quadratic		linear	
Average posterior probability	0.946	0.945		0.971	
Odds of correct classification [b]	23.52	17.59		412.86	
Observed probability [c]	0.430	0.494		0.075	
**Baseline characteristics associated with trajectory membership** [d]		**OR [95% CI]** [d]	***p*** [d]	**OR [95% CI]** [d]	***p*** [d]
Female	–	0.61 [0.36 - 1.01]	0.056	**0.37 [0.17 - 0.82]**	**0.014**
Age at motor-symptom onset	–	**1.12 [1.09 - 1.15]**	**<0.001**	**1.21 [1.16 - 1.27]**	**<0.001**
Years of education	–	**0.80 [0.74 - 0.86]**	**<0.001**	**0.65 [0.57 - 0.74]**	**<0.001**
Medical history					
Pesticide exposure	–	nr	nr	nr	nr
Head injury	–	nr	nr	nr	nr
Diabetes	–	**2.23 [1.10 - 4.53]**	**0.026**	2.57 [0.94 - 7.06]	0.066
REM sleep behavior disorder	–	nr	nr	nr	nr
Family history					
Parkinson's disease	–	nr	nr	nr	nr
Dementia	–	nr	nr	nr	nr
Tremor	–	nr	nr	nr	nr
Initial presentation					
Tremor-predominant	–	1.87 [0.94 - 3.71]	0.074	nr	nr
Akinetic/rigid predominant	–	0.56 [0.31 - 1.02]	0.056	nr	nr
Study site (weighted by size)					
DodoNA	–	nr	nr	nr	nr
LuxPark	–	nr	nr	nr	nr
ASAN	–	nr	nr	nr	nr
UT	–	nr	nr	nr	nr
LuxPark	–	nr	nr	nr	nr

[a] modeled using a censored normal probability distribution for the dependent variable

[b] based on the weighted posterior probability

[c] group probability based on the posterior probabilities

[d] compared to membership in reference trajectory, all models are censored normal. Odds ratios with $p<0.10$ are reported. OR, 95% CI, and p values are in bold if $p < 0.05$

Abbreviations – OR, odds ratio; 95% CI, 95% confidence interval; nr, values not reported ($p \geq 0.10$); NUST, St. Olav's Hospital, The Norwegian University of Science and Technology; ASAN, Asan Medical Center, University of Ulsan College of Medicine; UT, University Hospital of Larissa, University of Thessaly; LuxPark, The Luxembourg Parkinson's Study; DodoNA, The DodoNA project, NorthShore University HealthSystem.

Supplemental Table S11. Groups identified in trajectory models jointly considering UPDRS-III, Hoehn & Yahr, and MMSE scores and developed with the additional predictor *study site*

**Group**	**1 (reference)**	**2**		**3**	
N (% of 871)	400 (45.6)	286 (32.8)		184 (21.1)	
Fit [a]					
UPDRS-III	linear	quadratic		linear	
Hoehn & Yahr	linear	linear		linear	
MMSE	linear	quadratic		linear	
Average posterior probability	0.979	0.946		0.960	
Odds of correct classification [b]	53.64	37.50		89.98	
Observed probability [c]	0.468	0.317		0.214	
**Baseline characteristics associated with trajectory membership** [d]		**OR [95% CI]** [d]	***p*** [d]	**OR [95% CI]** [d]	***p*** [d]
Female	−	nr	nr	nr	nr
Age at motor-symptom onset	−	**1.06 [1.03 - 1.09]**	**<0.001**	**1.10 [1.08 - 1.14]**	**<0.0001**
Years of education	−	nr	nr	**0.88 [0.83 - 0.95]**	**<0.001**
Medical history					
Pesticide exposure	−	nr	nr	1.57 [0.92-2.67]	0.099
Head injury	−	nr	nr	nr	nr
Diabetes	−	nr	nr	nr	nr
REM sleep behavior disorder	−	nr	nr	nr	nr
Family history					
Parkinson’s disease	−	nr	nr	nr	nr
Dementia	−	nr	nr	nr	nr
Tremor	−	nr	nr	nr	nr
Initial presentation					
Tremor-predominant	−	nr	nr	**0.25 [0.14 - 0.48]**	**<0.001**
Akinetic/rigid predominant	−	nr	nr	nr	nr
Study site (weighted by size)					
DodoNA	−	nr	nr	nr	nr
NUST	−	nr	nr	nr	nr
ASAN	−	nr	nr	nr	nr
UT	−	nr	nr	nr	nr
LuxPark	−	nr	nr	nr	nr

[a] modeled using a censored normal probability distribution for the dependent variable

[b] based on the weighted posterior probability

[c] group probability based on the posterior probabilities

[d] compared to membership in reference trajectory, all models are censored normal. Odds ratios with $p<0.10$ are reported. OR, 95% CI, and p values are in bold if $p < 0.05$

Abbreviations – OR, odds ratio; 95% CI, 95% confidence interval; nr, values not reported ($p \geq 0.10$); NUST, St. Olav's Hospital, The Norwegian University of Science and Technology; ASAN, Asan Medical Center, University of Ulsan College of Medicine; UT, University Hospital of Larissa, University of Thessaly; LuxPark, The Luxembourg Parkinson's Study; DodoNA, The DodoNA project, NorthShore University HealthSystem.

Supplemental Table S12. Parkinson's disease outcomes in the LONG−PD cohort

Outcome	Study Site [a]					All N = 871
	Norwegian University of Science and Technology N = 77	ASAN LONG−PD N = 270	University of Thessaly N = 13	LuxPark N = 103	DodoNA N = 408	
Motor fluctuations	2 (2.6)	12 (4.4)	0 (0)	34 (33.0)	75 (18.4)	123 (14.1)
Dyskinesia	4 (5.2)	9 (3.3)	0 (0)	30 (29.1)	64 (15.7)	107 (12.3)
Persistent freezing	0 (0)	7 (2.6)	2 (15.4)	26 (25.2)	51 (12.5)	86 (9.9)
Persistent falls	0 (0)	2 (0.7)	0 (0)	25 (24.3)	63 (15.4)	90 (10.3)
Persistent orthostatism	2 (2.6)	5 (1.8)	1 (7.7)	53 (51.5)	29 (7.1)	90 (10.3)
Persistent urinary incontinence	8 (10.4)	9 (3.3)	3 (23.1)	57 (55.3)	47 (11.5)	124 (14.2)
Dysphagia	15 (9.5)	16 (5.9)	0 (0)	53 (51.5)	69 (16.9)	153 (17.6)
REM sleep behavior disorder [b]	13 (17.3)	151 (60.4)	0 (0)	31 (35.6)	83 (21.1)	278 (33.9)
Cognitive impairment	23 (29.9)	45 (16.7)	0 (0)	72 (69.9)	146 (35.8)	286 (32.8)
Psychosis	7 (9.1)	4 (1.5)	0 (0)	29 (28.2)	20 (4.9)	60 (6.9)
Impulse control disorder	0 (0)	0 (0)	0 (0)	27 (26.2)	0 (0)	27 (3.10)

[a] Table cells show the number (percent) with the outcome. In χ^2 tests, all outcomes differed by site at $p < 0.001$

[b] N = 819. Patients with REM sleep behavior disorder at the initial visit were excluded from this tabulation and Kaplan-Meier survival analyses

Supplemental Table S13. Patterns of significant differences in the survival free of an outcome across trajectory groups modeled with the additional predictors *years of education* and *study site* [a]

Outcome	Assessment Used in Group-Based-Trajectory Model						Figures
	UPDRS-III Score	UPDRS-III Tremor Subscore	UPDRS-III Bradykinesia-Rigidity Subscore	Hoehn & Yahr Stage	MMSE Score	UPDRS-III, Hoehn & Yahr, MMSE Multivariable	
Motor fluctuations	ns	ns	ns	ns	ns	ns	S20
Dyskinesia	ns	[1]<[3]	ns	ns	ns	[3]<[2]	S21
Persistent freezing	[3]<[2]<[1]	ns	[2, 3]<[1]	[3]<[1,2]	[3]<[1]	[3]<[1,2]	S16, S22
Persistent falls	[3]<[2]<[1]	ns	[2,3]<[1]	[3]<[2]<[1]	[2,3]<[1]	[3]<[1,2]	S16, S23
Persistent orthostatism	[3]<[2]<[1]	ns	ns	ns	ns	[2,3]<[1]	S17, S24
Persistent urinary incontinence	[3]<[2]<[1}	ns	ns	[3]<[2]	[3]<[1]	[2,3]<[1]	S17, S25
Dysphagia	[3]<[1,2]	ns	ns	[3]<[1,2]	ns	[2,3]<[1]	S16, S27
REM sleep behavior disorder[b]	[2,3]<[1]	ns	[3]<[1]	ns	ns	[2]<[1,3]	S28
Cognitive impairment	[3]<[2]<[1]	ns	[3]<[1,2]	[3]<[2]<[1]	[3]<[2]<[1]	[3]<[2]<[1]	S16, S29
Psychosis	[3]<[1,2]	ns	ns	[3]<[2]	[3]<[1]	[2,3]<[1]	S16, S30
Impulse control disorder	[3]<[2]>[1]	nd	nd	[1]<[2]	ns	[2]<[1,3]	S31

[a] If a log-rank test revealed a significant difference between an outcome across a set of three trajectory groups, pairwise log-rank tests were used to assess differences between pairs of trajectory groups. The table reports trajectory groups that show significant differences for an outcome after Bonferroni correction for multiple tests. A bracketed number refers to a trajectory group for an assessment. A trajectory group to the left of the "<" sign showed poorer survival free of the outcome than a trajectory group to the right. In some cases, no pairs or only one pair of trajectory groups showed differences that remained significant following Bonferroni correction. Compare to the Kaplan-Meier survival probability plotted in the indicated figure, and compare the patterns tabulated here to those in Table 3, which shows the patterns of survival free of an outcome for trajectory groups modeled without *site* and, for models not assessing MMSE score, *years of education*, as additional predictors. Differences from Table 3 are highlighted in yellow. These are associated with changes in group-membership composition in the two types of models, usually 1-5% of the group membership. In most cases, differences from the patterns shown in Table 3 reflect whether a single-intergroup difference reached, or failed to retain, significance following a Bonferroni correction for multiple testing.

[b] Patients with REM sleep behavior disorder at the initial visit were excluded from these analyses.

Abbreviations: UPDRS-III: Unified Parkinson's Disease Rating Scale part III (motor); MMSE: Mini-Mental Status Exam; ns: not significant, either a log-rank test failed to identify significant differences in survival free of the outcome across the three trajectory groups or pairwise differences were not significant following Bonferroni correction for multiple tests; nd: not done, none of the patients with available UPDRS-III tremor subscore and bradykinesia-rigidity subscore data developed impulse control disorder.

Supplemental Figure S1. Motor-score trajectories seen in the LONG-PD cohort Group-based trajectory modeling identified three trajectory-groups for (A) total UPDRS-III scores, (B) UPDRS-III tremor subscores, and (C) UPDRS-III bradykinesia-rigidity subscores. UPDRS-III tremor and bradykinesia-rigidity subscores were available for only 678 participants. Membership in most trajectory-groups converge (panels D-F) with <5% misclassification (dashed teal line) (panels G-I) by four-to-five years after the onset of the initial motor symptom. All converge with <4% misclassification by six years. Trajectories were modeled jointly with the predictors: sex, age at motor-symptom onset, pesticide exposure, head injury, diabetes, REM-behavior sleep disorder, family history (Parkinson's disease, dementia, or tremor), and initial presentation (tremor-predominant, akinetic/rigid predominant).

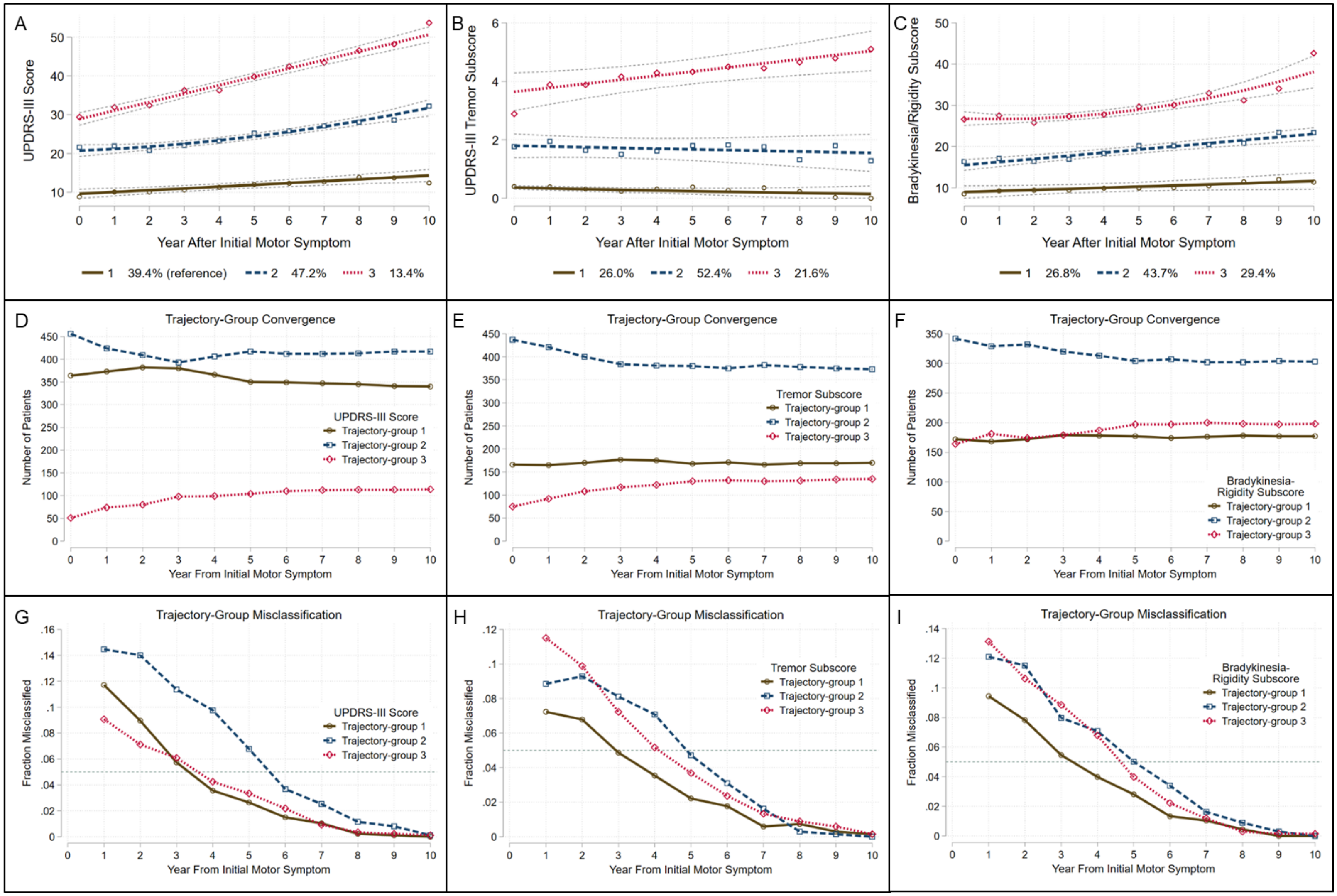

Supplemental Figure S2. Disease-stage and cognitive trajectories seen in the LONG-PD cohort Group-based trajectory modeling identified three trajectory-groups for (A) Hoehn and Yahr stage and (B) Mini-mental status exam (MMSE) score. Trajectory-groups for Hoehn and Yahr stage (C, E) and MMSE-score (D, F) converge with <5% misclassification (dashed teal line) by about five years after the onset of the initial motor symptom. Trajectories were modeled jointly with the predictors: sex, age at motor-symptom onset, pesticide exposure, head injury, diabetes, REM-behavior sleep disorder, family history (Parkinson's disease, dementia, or tremor), initial presentation (tremor-predominant, akinetic/rigid predominant), and for MMSE-score only, years of education.

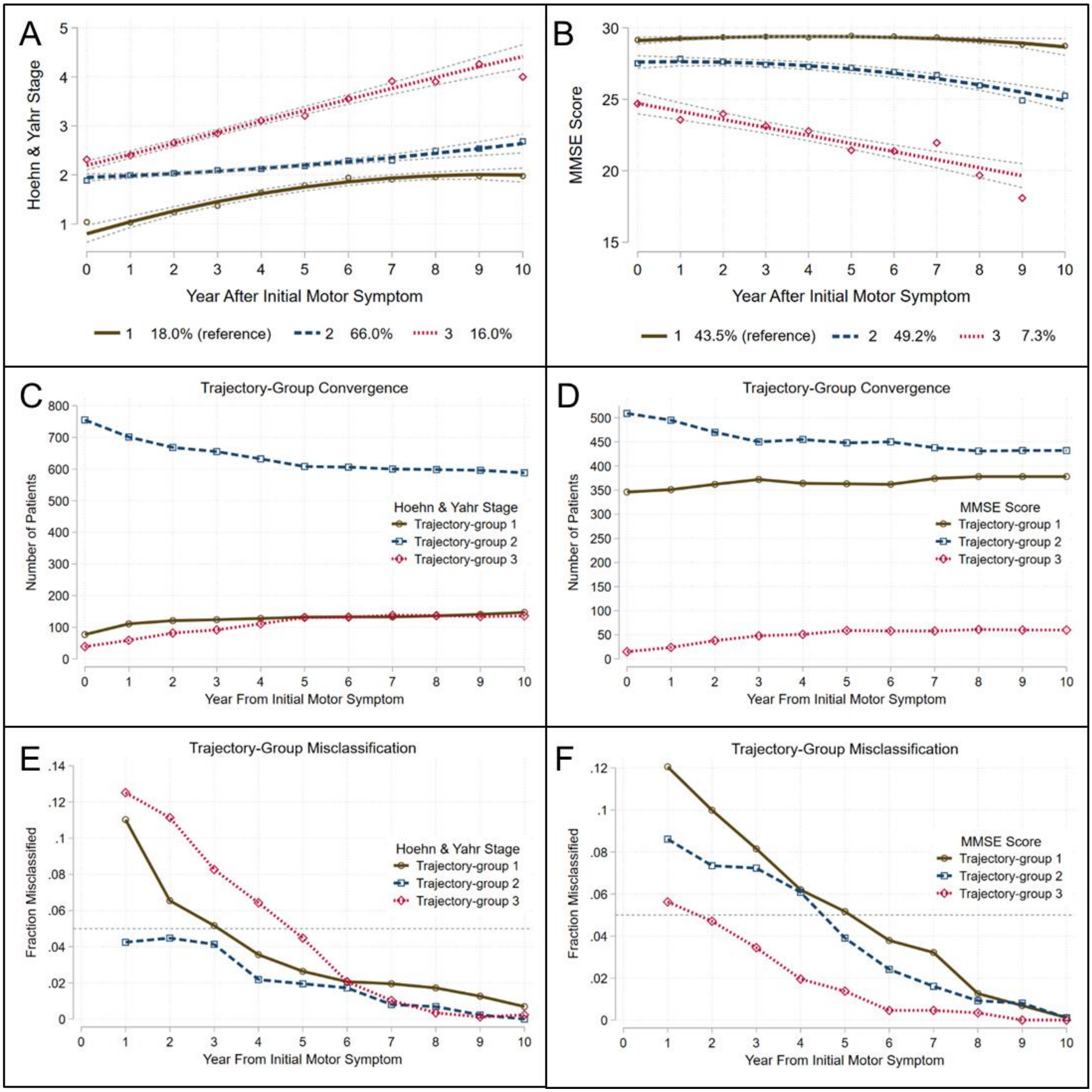

Supplemental Figure S3. Survival free of motor fluctuations Kaplan-Meier analyses for survival free of motor fluctuations across trajectory-groups modeled jointly with the predictors: sex, age at motor-symptom onset, pesticide exposure, head injury, diabetes, REM-behavior sleep disorder, family history (Parkinson's disease, dementia, or tremor), initial presentation (tremor-predominant, akinetic/rigid predominant), and, for assessments including Mini-Mental Status Exam (MMSE) score, years of education. (A) UPDRS-III score, (B) Hoehn & Yahr (H&Y) stage, (C) UPDRS−III-tremor subscore, (D) MMSE, and (E) UPDRS−III-bradykinesia-rigidity subscore. An at-risk table beneath each plot shows the number at-risk at each time point, with the number of failed (outcome reached) events listed in parentheses. Log-rank test results are shown. An asterisk identifies pairs of trajectory-groups where outcomes differ in pairwise log-rank tests with a Bonferroni-corrected $p<0.05$. Though the overall log-rank test was significant for differences across UPDRS-III trajectory groups, no pairwise differences between these groups were significant after a multiple-testing correction. For H&Y-stage trajectory-groups, motor fluctuation outcomes are poorer in Group 3 having a severe trajectory than in Group 1 having a more benign trajectory.

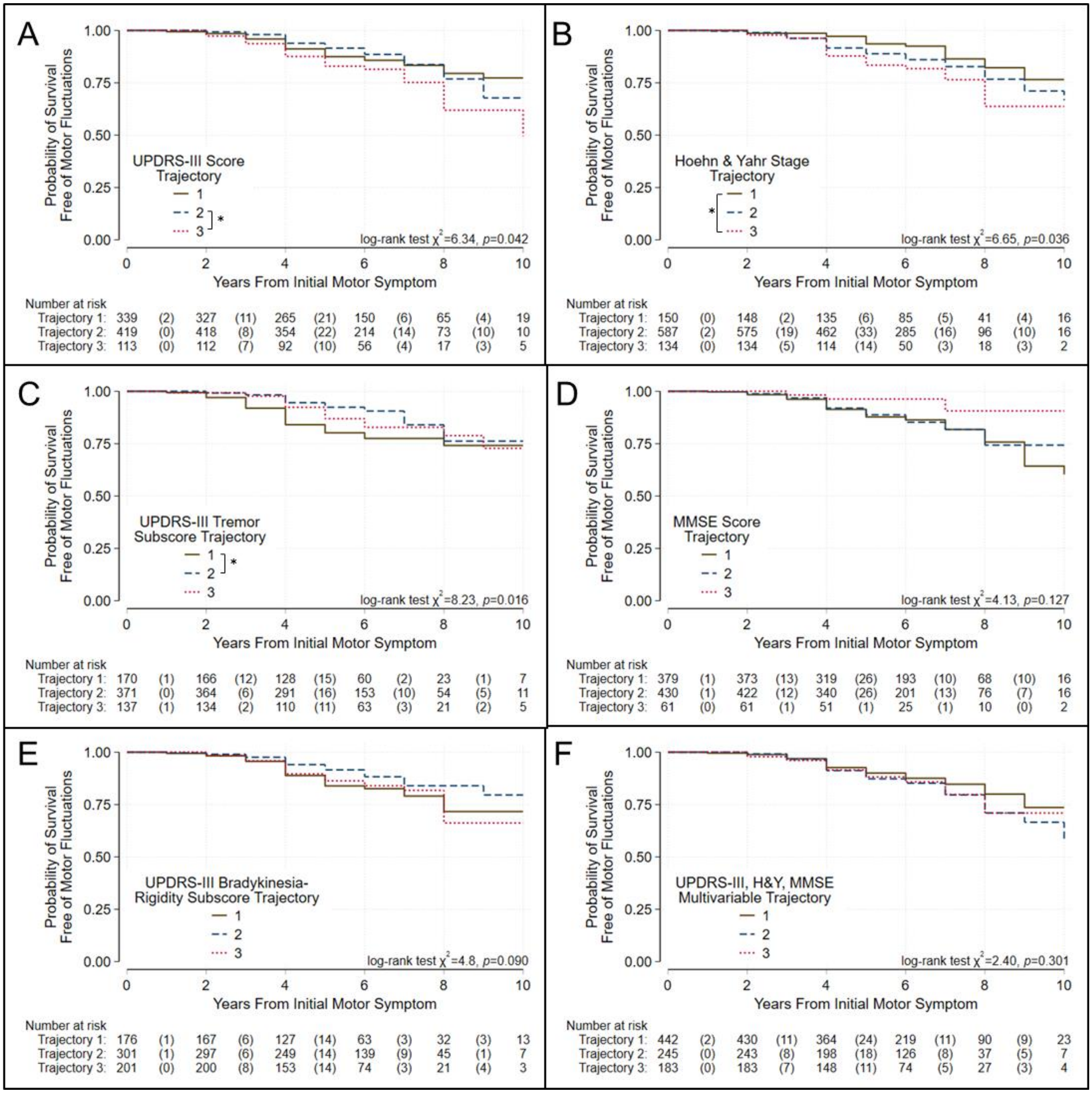

Supplemental Figure S4. Survival free of dyskinesias Kaplan-Meier analyses for survival free of dyskinesias in trajectory-groups modeled jointly with the predictors: sex, age at motor-symptom onset, pesticide exposure, head injury, diabetes, REM-behavior sleep disorder, family history (Parkinson's disease, dementia, or tremor), initial presentation (tremor-predominant, akinetic/rigid predominant), and, for assessments including Mini-Mental Status Exam (MMSE) score, years of education. (A) UPDRS-III score, (B) Hoehn & Yahr (H&Y) stage, (C) UPDRS−III-tremor subscore, (D) MMSE, and (E) UPDRS−III-bradykinesia-rigidity subscore. The at-risk table beneath each plot shows the number at-risk at each time point, with the number of failed (outcome reached) events listed in parentheses. Log-rank test results are shown. An asterisk identify pairs of trajectory-groups where outcomes differ in pairwise log-rank tests with a Bonferroni-corrected $p<0.05$. For the UPDRS−III-tremor-subscore trajectory-groups, the outcome of dyskinesias was poorer in Group 1 having a more benign trajectory than in Group 3 having a severe trajectory. Though the overall log-rank test was significant for differences across the H&Y-trajectory groups, no pairwise differences between these groups were significant after a multiple-testing correction.

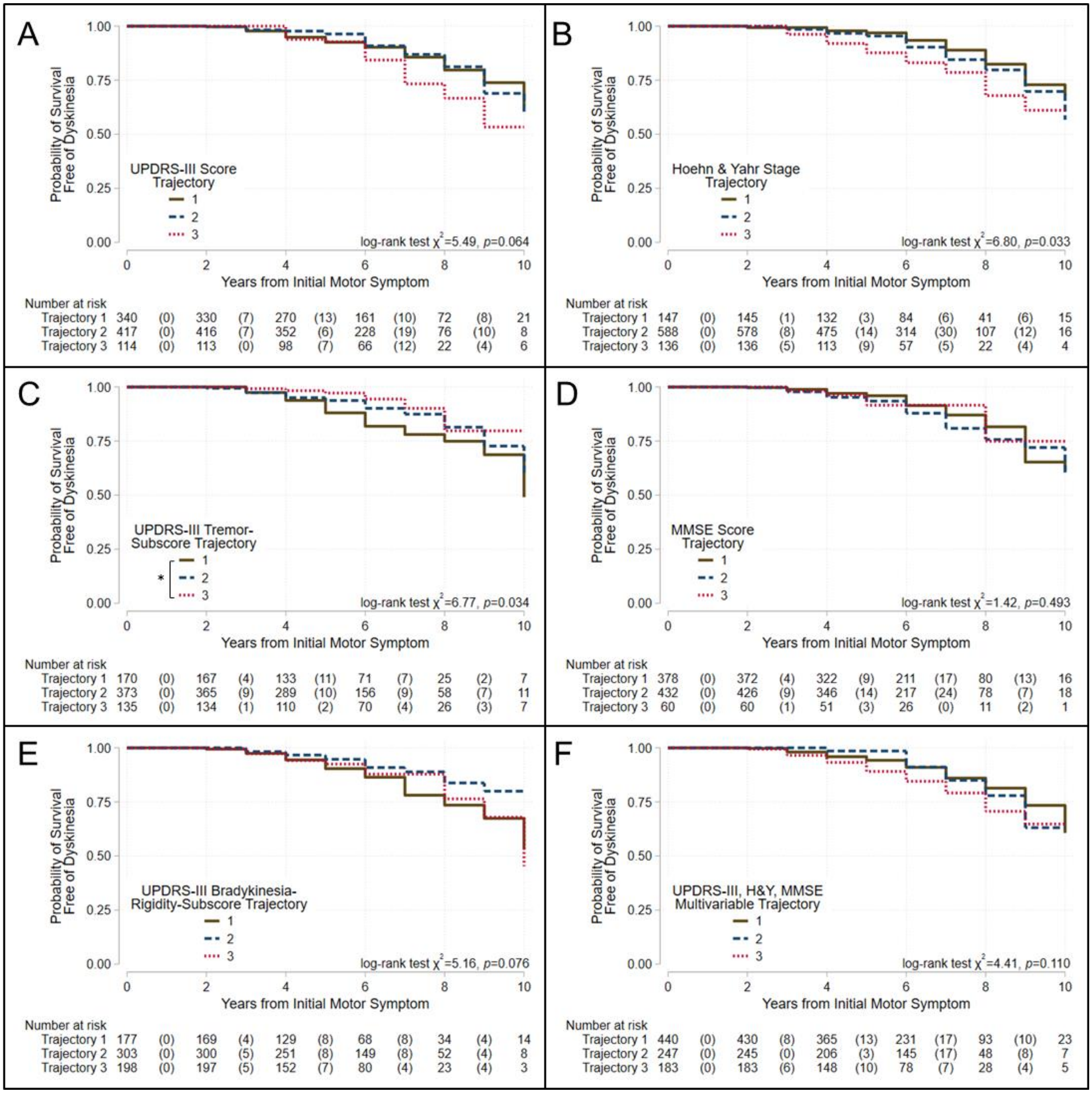

Supplemental Figure S5. Survival free of persistent freezing Kaplan-Meier analyses for survival free of persistent freezing in trajectory-groups modeled jointly with the predictors: sex, age at motor-symptom onset, pesticide exposure, head injury, diabetes, REM-behavior sleep disorder, family history (Parkinson's disease, dementia, or tremor), initial presentation (tremor-predominant, akinetic/rigid predominant, and, for assessments including Mini-Mental Status Exam (MMSE) score, years of education. (A) UPDRS-III score, (B) Hoehn & Yahr (H&Y) stage, (C) UPDRS−III-tremor subscore, (D) MMSE, and (E) UPDRS−III-bradykinesia-rigidity subscore. The at-risk table beneath each plot shows the number at-risk at each time point, with the number of failed (outcome reached) events listed in parentheses. Log-rank test results are shown. Asterisks identify pairs of trajectory-groups where outcomes differ in pairwise log-rank tests with a Bonferroni-corrected p<0.05 (*), p<0.01 (**), or p<0.001 (***). For the UPDRS−III-score trajectory-groups, the outcome of persistent freezing was poorest in Group 3 having a severe trajectory, less poor in Group 2 having an intermediate trajectory, and least poor in Group 1 having a more benign trajectory. For the UPDRS-III-bradykinesia-rigidity–subscore trajectory-groups, it was similar in Groups 2 and 3, which have intermediate and severe trajectories, respectively, and poorer than in Group 1 having a more benign trajectory. For the H&Y-stage trajectory-groups, it is poorer in Group 3 having a severe trajectory and similar and less poor in Groups 1 and 2 having more benign and intermediate trajectories, respectively. For the MMSE-score trajectory-groups, the outcome was poorer in Group 3 having a severe trajectory than Group 1 having a more benign trajectory.

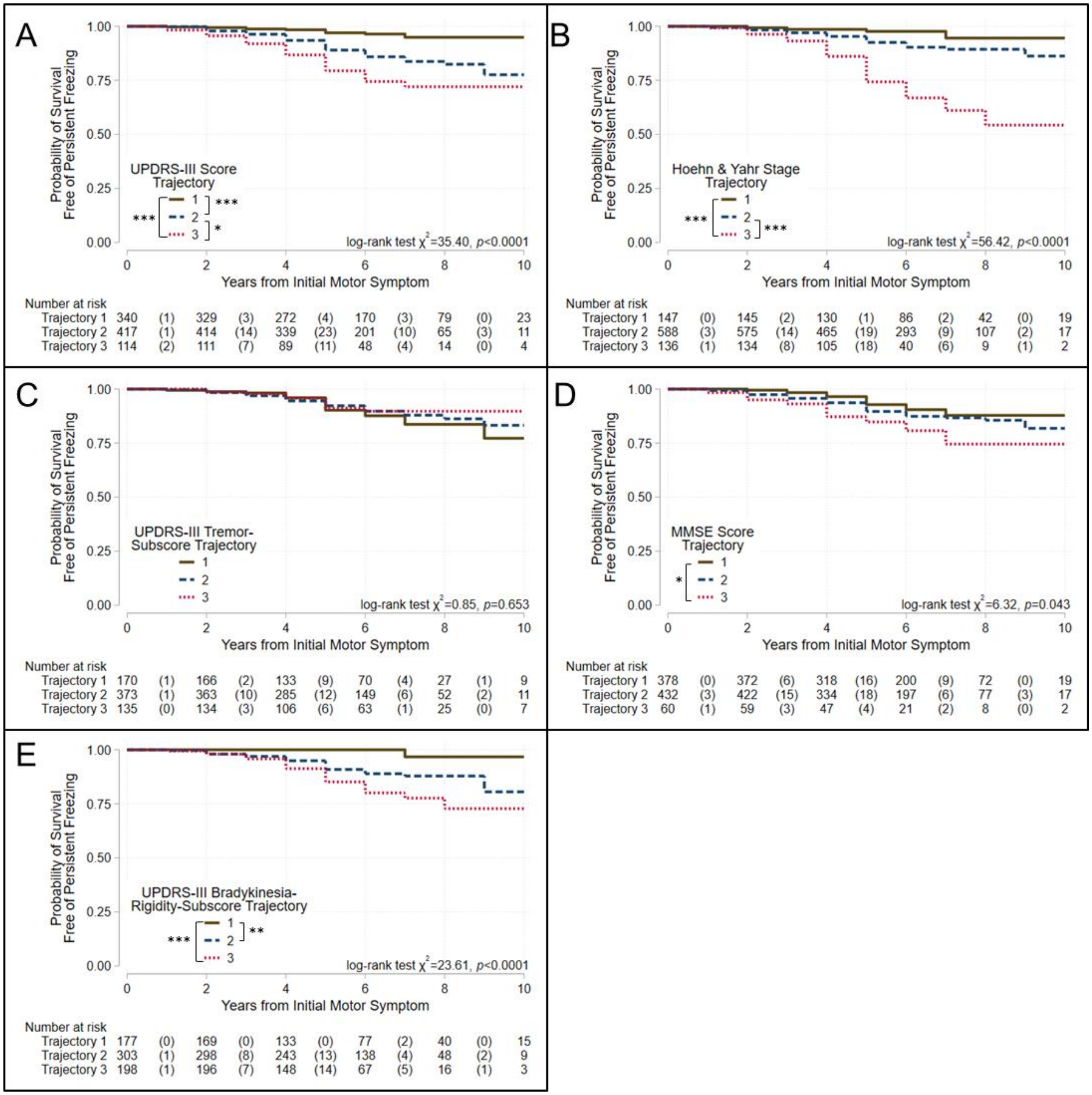

Supplemental Figure S6. Survival free of persistent falls Kaplan-Meier analyses for survival free of persistent falls in trajectory-groups modeled jointly with the predictors: sex, age at motor-symptom onset, pesticide exposure, head injury, diabetes, REM-behavior sleep disorder, family history (Parkinson's disease, dementia, or tremor), initial presentation (tremor-predominant, akinetic/rigid predominant), and, for assessments including Mini-Mental Status Exam (MMSE) score, years of education. (A) UPDRS-III score, (B) Hoehn & Yahr (H&Y) stage, (C) UPDRS−III-tremor subscore, (D) MMSE, and (E) UPDRS−III-bradykinesia-rigidity subscore. The at-risk table beneath each plot shows the number at-risk at each time point, with the number of failed (outcome reached) events listed in parentheses. Log-rank test results are shown. Asterisks identify pairs of trajectory-groups where outcomes differ in pairwise log-rank tests with a Bonferroni-corrected $p<0.05$ (*), $p<0.01$ (**), or $p<0.001$ (***). For the H&Y-stage trajectory-groups, the outcome of persistent falls was poorest in Group 3 having the most severe trajectory, less poor in Group 2 having an intermediate trajectory, and least poor in Group 2 having a more benign trajectory. For the UPDRS-III score trajectory-groups, it was poorest in Group 3 having the most severe trajectory. For the MMSE-score and UPDRS-III-bradykinesia-rigidity-subscore trajectory-groups, it was similar in Groups 2 and 3, but poorer than in Group 1 having the more benign trajectory.

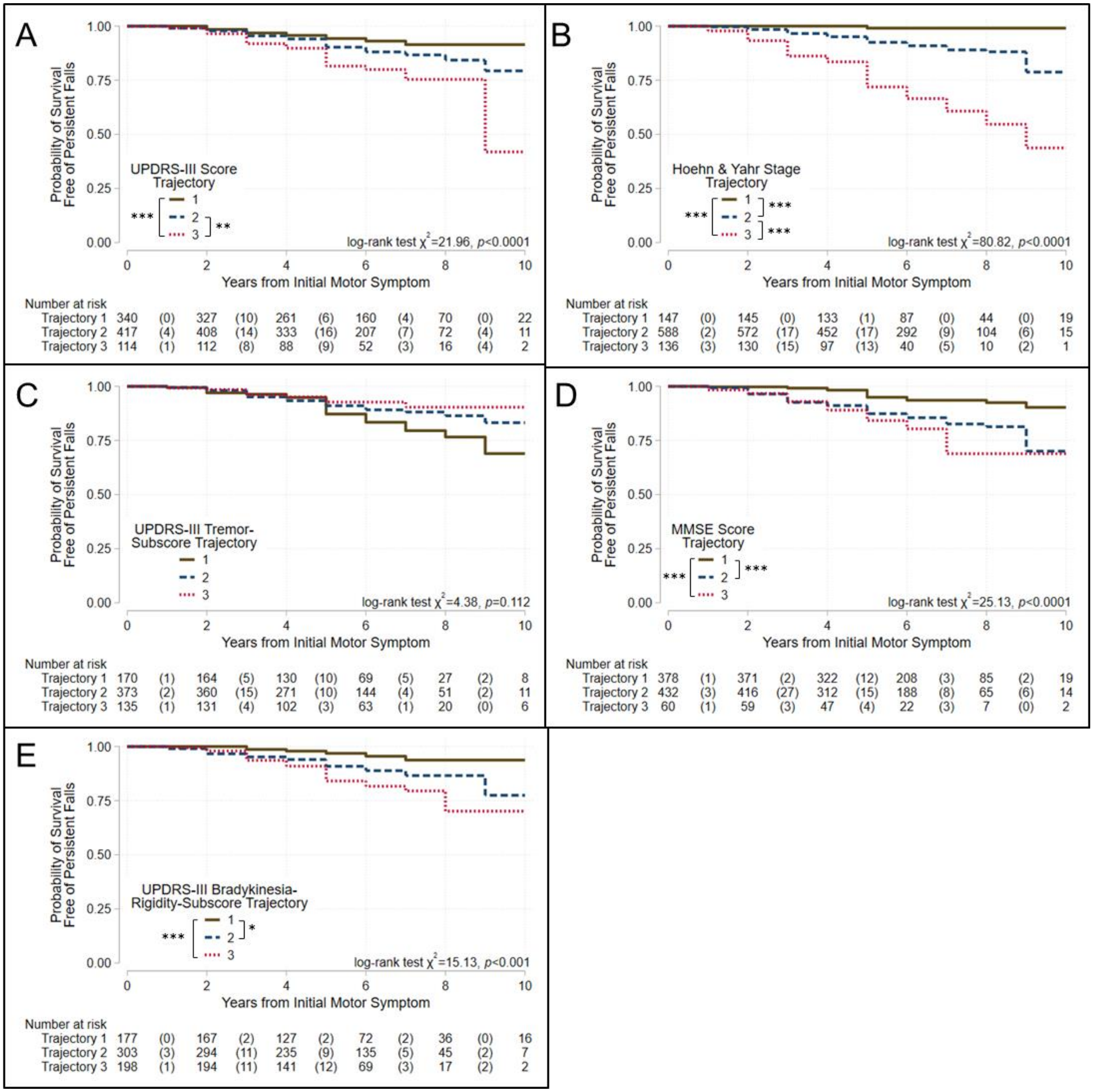

Supplemental Figure S7. Survival free of persistent orthostatism Kaplan-Meier analyses for survival free of persistent orthostatism in trajectory-groups modeled jointly with the predictors: sex, age at motor-symptom onset, pesticide exposure, head injury, diabetes, REM-behavior sleep disorder, family history (Parkinson's disease, dementia, or tremor), initial presentation (tremor-predominant, akinetic/rigid predominant), and for MMSE-score, years of education. (A) UPDRS-III score, (B) Hoehn & Yahr (H&Y) stage, (C) UPDRS−III-tremor subscore, (D) Mini-Mental Status Exam (MMSE), and (E) UPDRS−III-bradykinesia-rigidity subscore. The at-risk table beneath each plot shows the number at-risk at each time point, with the number of failed (outcome reached) events listed in parentheses. Log-rank test results are shown. Asterisks identify pairs of trajectory-groups where outcomes differ in pairwise log-rank tests with a Bonferroni-corrected *p*<0.01 (**) or *p*<0.001 (***). For the UPDRS-III score trajectory-groups, the outcome of persistent orthostatism was poorest was Group 3 having the most severe trajectory, less poor in Group 2 having the intermediate trajectory, and least poor in Group 1 having a more benign trajectory.

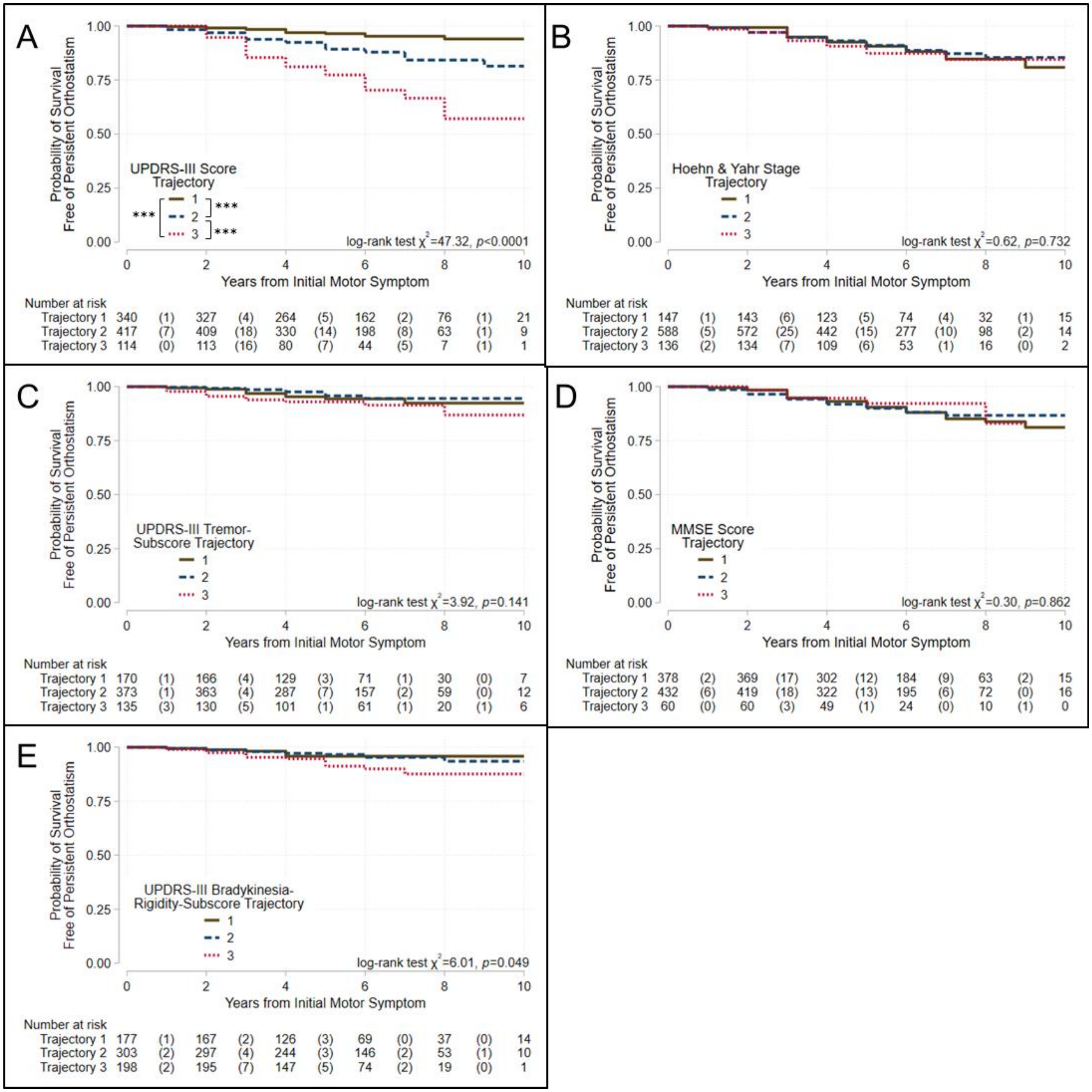

Supplemental Figure S8. Survival free of persistent urinary incontinence Kaplan-Meier analyses for survival free of persistent urinary incontinence in trajectory-groups modeled jointly with the predictors: sex, age at motor-symptom onset, pesticide exposure, head injury, diabetes, REM-behavior sleep disorder, family history (Parkinson's disease, dementia, or tremor), initial presentation (tremor-predominant, akinetic/rigid predominant), and for MMSE-score, years of education. (A) UPDRS-III score, (B) Hoehn & Yahr (H&Y) stage, (C) UPDRS−III-tremor subscore, (D) Mini-Mental Status Exam (MMSE), and (E) UPDRS−III-bradykinesia-rigidity subscore. The at-risk table beneath each plot shows the number at-risk at each time point, with the number of failed (outcome reached) events listed in parentheses. Log-rank test results are shown. Asterisks identify pairs of trajectory-groups where outcomes differ in pairwise log-rank tests with a Bonferroni-corrected $p<0.05$ (*) or $p<0.001$ (***). For the UPDRS−III-score trajectory-groups, the outcome of persistent urinary incontinence was poorest in Group 3 having a severe trajectory, while it was similar and less poor in Groups 2 and 1 having intermediate and more benign trajectories, respectively. For the MMSE-score trajectory-groups, it was poorer in Group 3 having the most severe trajectory than in Group 1 having the more benign trajectory.

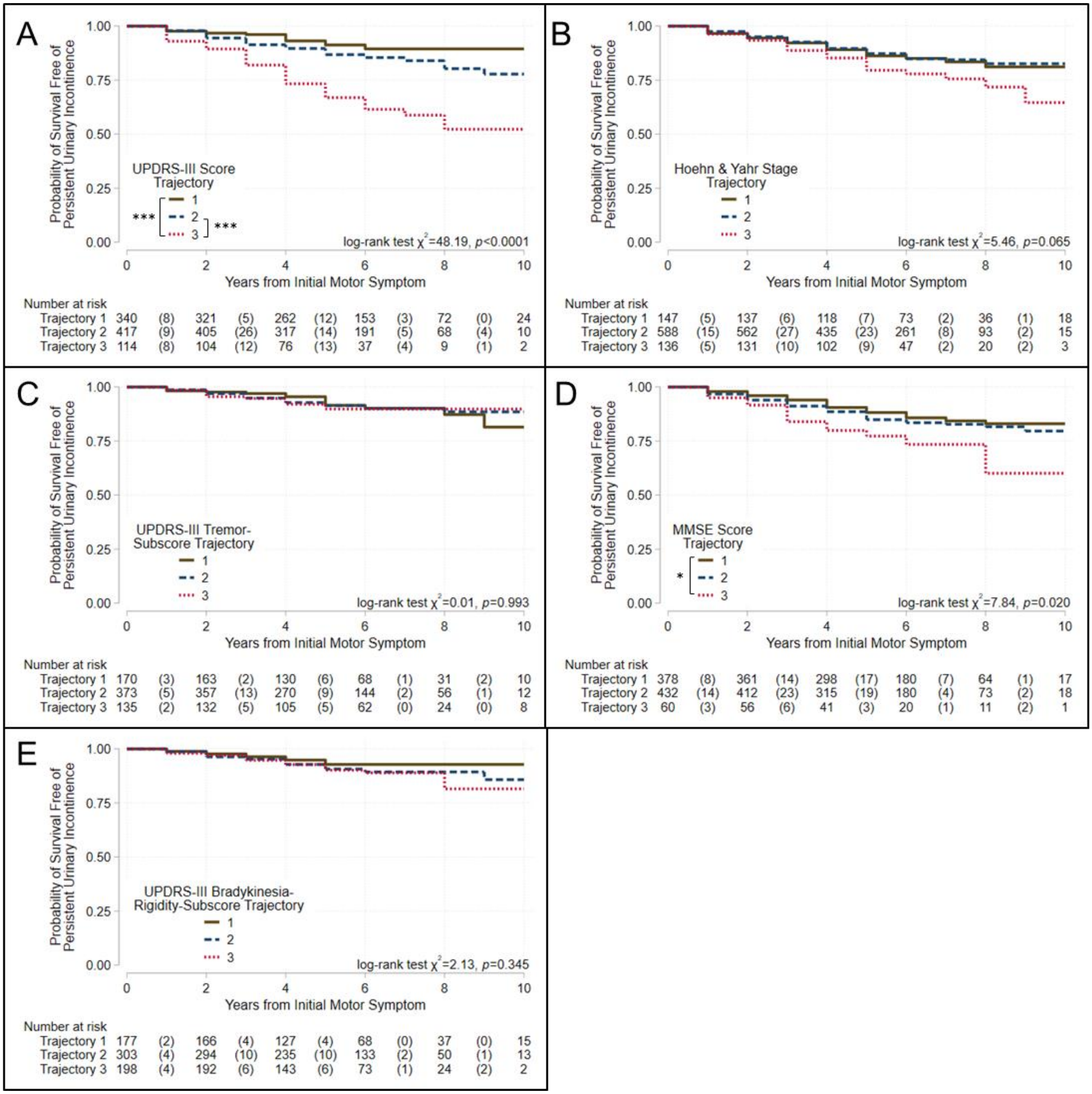

Supplemental Figure S9. Survival free of dysphagia Kaplan-Meier analyses for survival free of dysphagia in trajectory-groups modeled jointly with the predictors: sex, age at motor-symptom onset, pesticide exposure, head injury, diabetes, REM-behavior sleep disorder, family history (Parkinson's disease, dementia, or tremor), initial presentation (tremor-predominant, akinetic/rigid predominant), and for MMSE-score, years of education. (A) UPDRS-III score, (B) Hoehn & Yahr (H&Y) stage, (C) UPDRS−III-tremor subscore, (D) Mini-Mental Status Exam (MMSE), and (E) UPDRS−III-bradykinesia-rigidity subscore. The at-risk table beneath each plot shows the number at-risk at each time point, with the number of failed (outcome reached) events listed in parentheses. Log-rank test results are shown. Asterisks identify pairs of trajectory-groups where outcomes differ in pairwise log-rank tests with a Bonferroni-corrected *p*<0.05 (*), *p*<0.01 (**), or *p*<0.001 (***). For both the UPDRS−III-score and the H&Y-stage trajectory-groups, the outcome of dysphagia was poorest in Group 3 having the most severe trajectory, and similar but less poor in Groups 1 and 2 having more benign or intermediate trajectories, respectively.

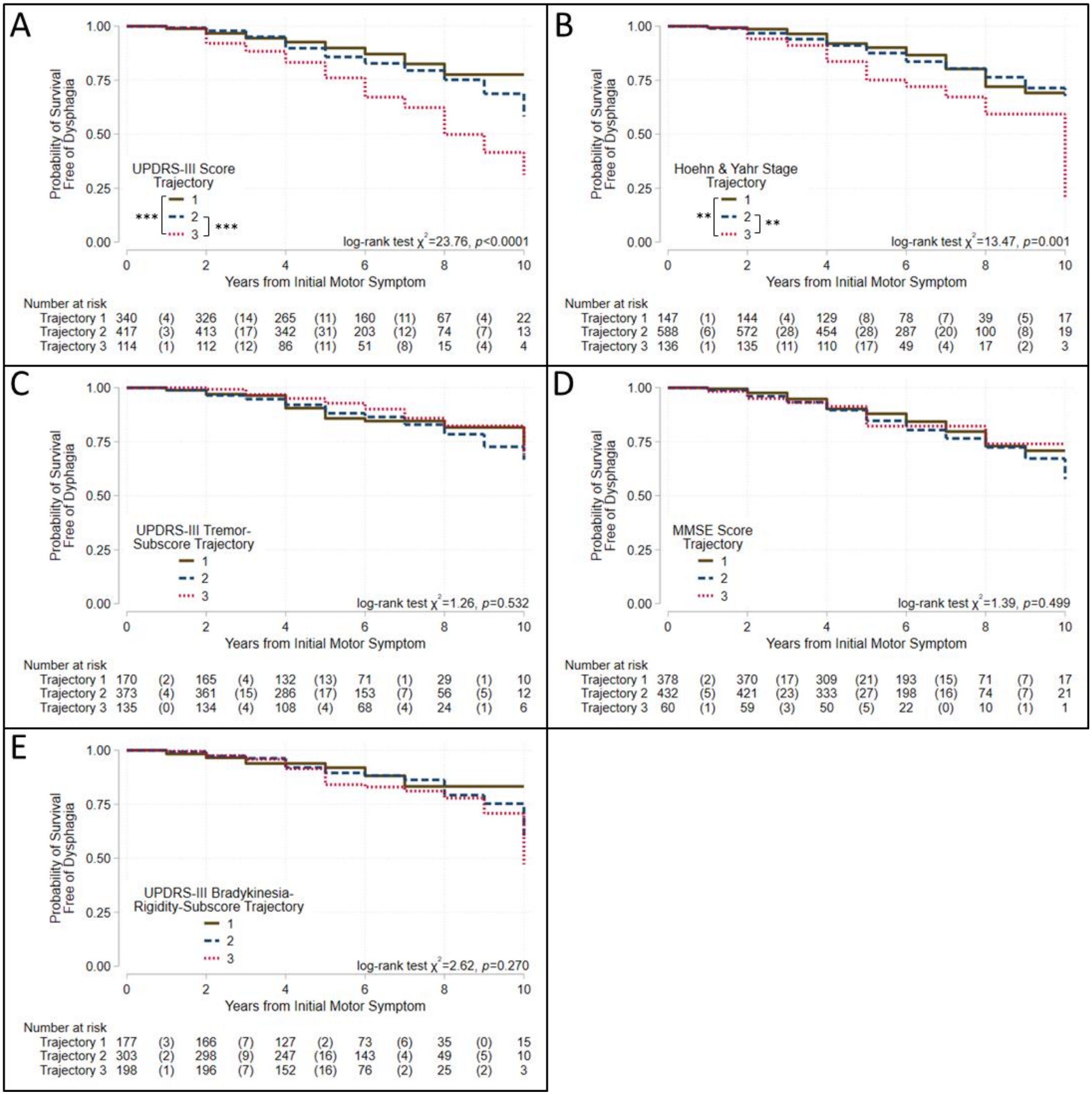

Supplemental Figure S10. Survival free of REM sleep behavior disorder The figures present Kaplan-Meier analyses for survival free of REM sleep behavior disorder, excluding individuals presenting with REM sleep disorder at the initial visit. Trajectory-groups were modeled jointly with the predictors: sex, age at motor-symptom onset, pesticide exposure, head injury, diabetes, family history (Parkinson's disease, dementia, or tremor), initial presentation (tremor-predominant, akinetic/rigid predominant), and, for assessments including Mini-Mental Status Exam (MMSE) score, years of education. (A) UPDRS-III score, (B) Hoehn & Yahr (H&Y) stage, (C) UPDRS−III-tremor subscore, (D) Mini-Mental Status Exam (MMSE), and (E) UPDRS−III-bradykinesia-rigidity subscore. The at-risk table beneath each plot shows the number at-risk at each time point, with the number of failed (outcome reached) events listed in parentheses. Log-rank test results are shown. Asterisks identify pairs of trajectory-groups where outcomes differ in pairwise log-rank tests with a Bonferroni-corrected *p*<0.01 (**) or *p*<0.001 (***). In the UPDRS-III score trajectory-groups, the outcome of REM sleep behavior disorder was similar in Groups 2 and 3 having intermediate and severe trajectories, respectively, and poorer than in Group 1 having a more benign trajectory. Across the UPDRS-III-bradykinesia-rigidity subscore trajectory-groups, it was poorer in Group 3 having a more severe trajectory than in Group 1 having a more benign trajectory.

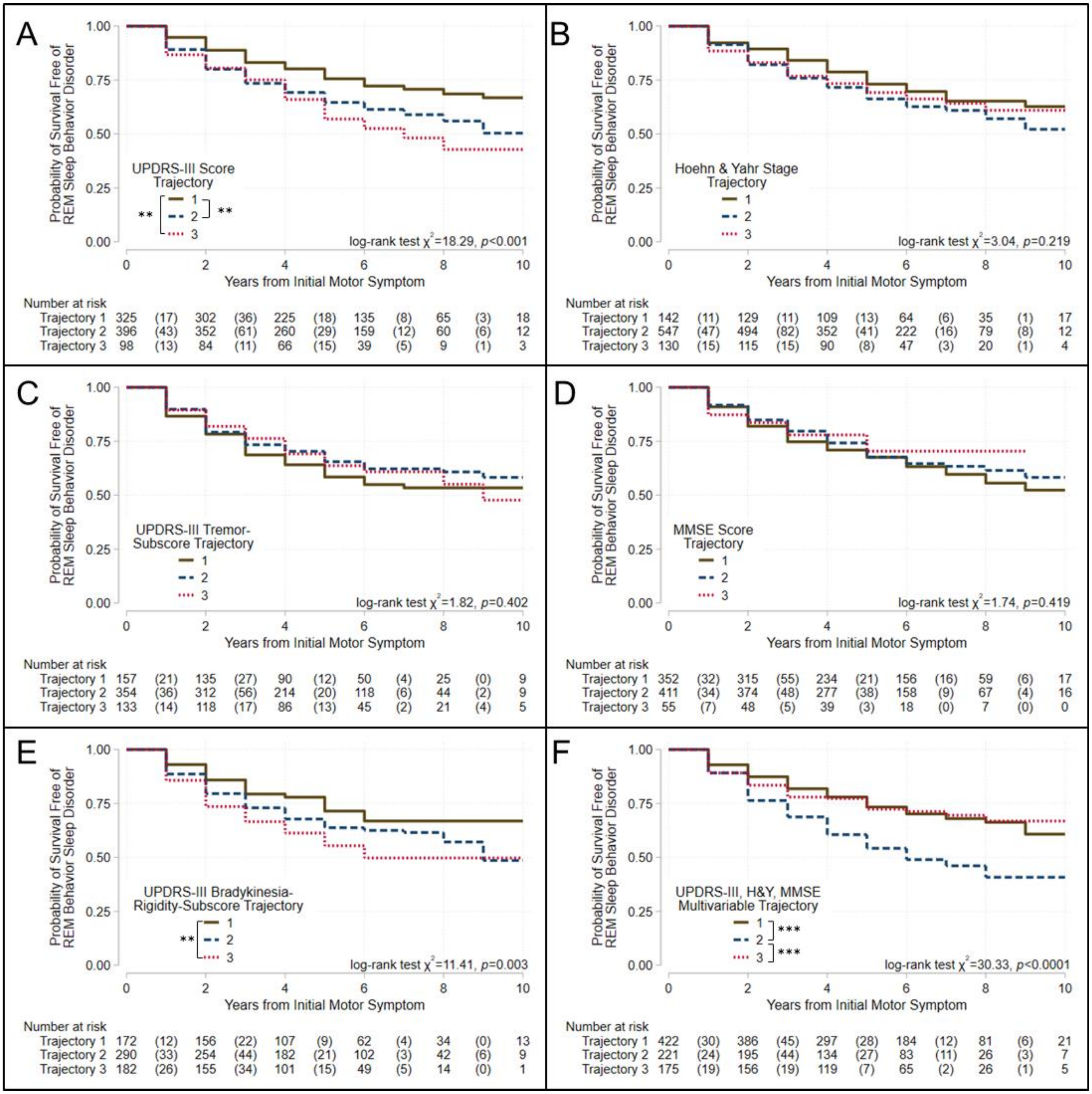

Supplemental Figure S11. Survival free of cognitive impairment Kaplan-Meier analyses for survival free of cognitive impairment in trajectory-groups modeled jointly with the predictors: sex, age at motor-symptom onset, pesticide exposure, head injury, diabetes, REM-behavior sleep disorder, family history (Parkinson's disease, dementia, or tremor), initial presentation (tremor-predominant, akinetic/rigid predominant), and for MMSE-score, years of education. (A) UPDRS-III score, (B) Hoehn & Yahr (H&Y) stage, (C) UPDRS−III-tremor subscore, (D) Mini-Mental Status Exam (MMSE), and (E) UPDRS−III-bradykinesia-rigidity subscore. The at-risk table beneath each plot shows the number at-risk at each time point, with the number of failed (outcome reached) events listed in parentheses. Log-rank test results are shown. Asterisks identify pairs of trajectory-groups where outcomes differ in pairwise log-rank tests with a Bonferroni-corrected $p<0.01$ (**) or $p<0.001$ (***). In all trajectory-groups except those for the UPDRS−III-tremor subscore, the outcome of cognitive impairment was poorest in Group 3 having the most severe trajectory. In most trajectory-groups, it was less poor in Group 2 having an intermediate trajectory and least poor in Group 1 having a more benign trajectory.

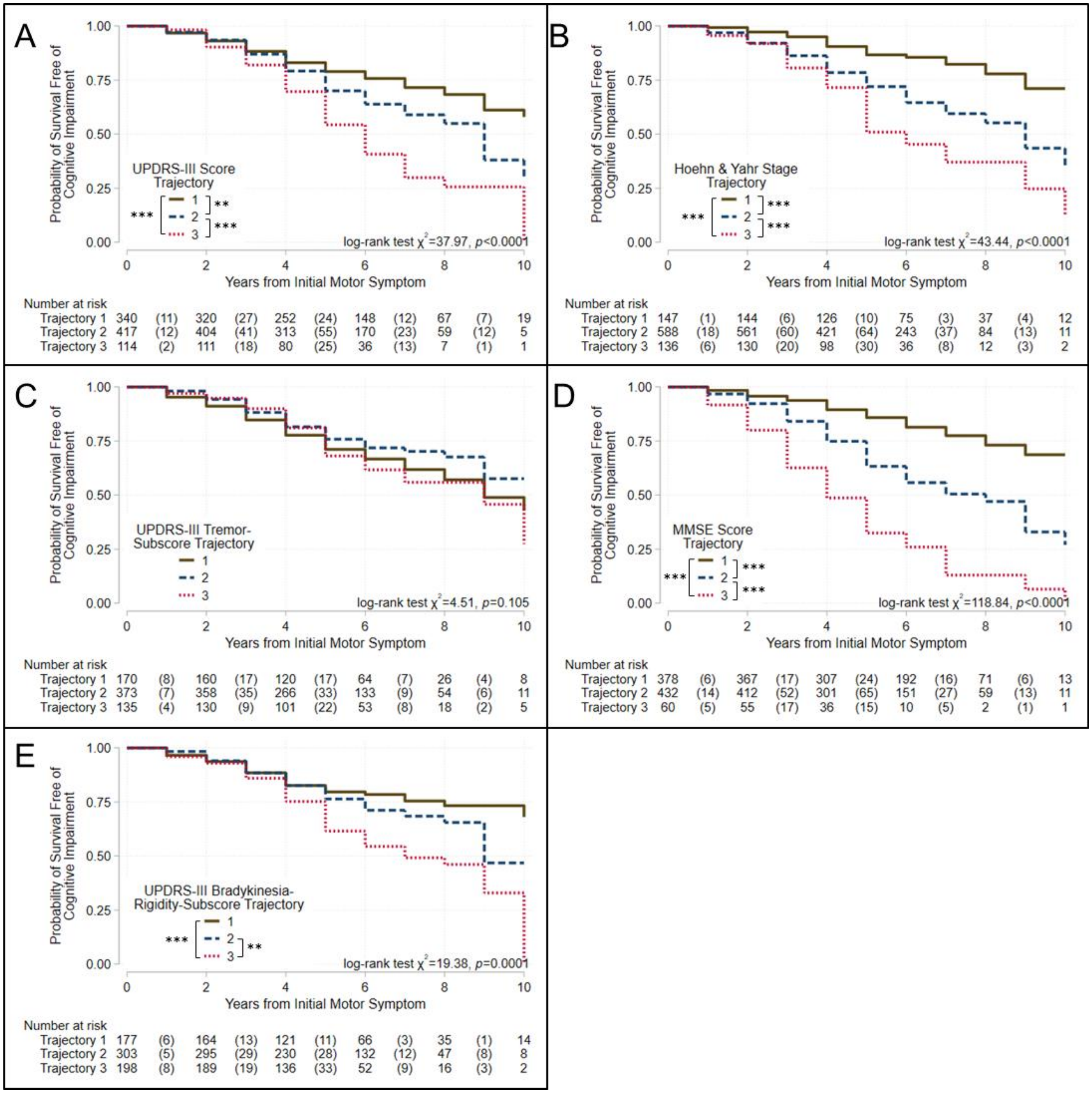

Supplemental Figure S12. Survival free of psychosis Kaplan-Meier analyses for survival free of psychosis in trajectory-groups modeled jointly with the predictors: sex, age at motor-symptom onset, pesticide exposure, head injury, diabetes, REM-behavior sleep disorder, family history (Parkinson's disease, dementia, or tremor), initial presentation (tremor-predominant, akinetic/rigid predominant), and for MMSE-score, years of education. (A) UPDRS-III score, (B) Hoehn & Yahr (H&Y) stage, (C) UPDRS−III-tremor subscore, (D) Mini-Mental Status Exam (MMSE), and (E) UPDRS−III-bradykinesia-rigidity subscore. The at-risk table beneath each plot shows the number at-risk at each time point, with the number of failed (outcome reached) events listed in parentheses. Log-rank test results are shown. Asterisks identify pairs of trajectory-groups where outcomes differ in pairwise log-rank tests with a Bonferroni-corrected p<0.05 (*), p<0.01(**), or p<0.001(***). In the UPDRS−III-score trajectory-groups, the outcome of psychosis was poorer in Group 3 having the most severe trajectory, while it was similar, but less poor, in Groups 2 and 1 having intermediate or more benign disease courses, respectively. In the H&Y-stage and MMSE-score trajectory-groups, the outcome is poorer in Group 3 having a severe trajectory than in Group 1 having a more benign trajectory.

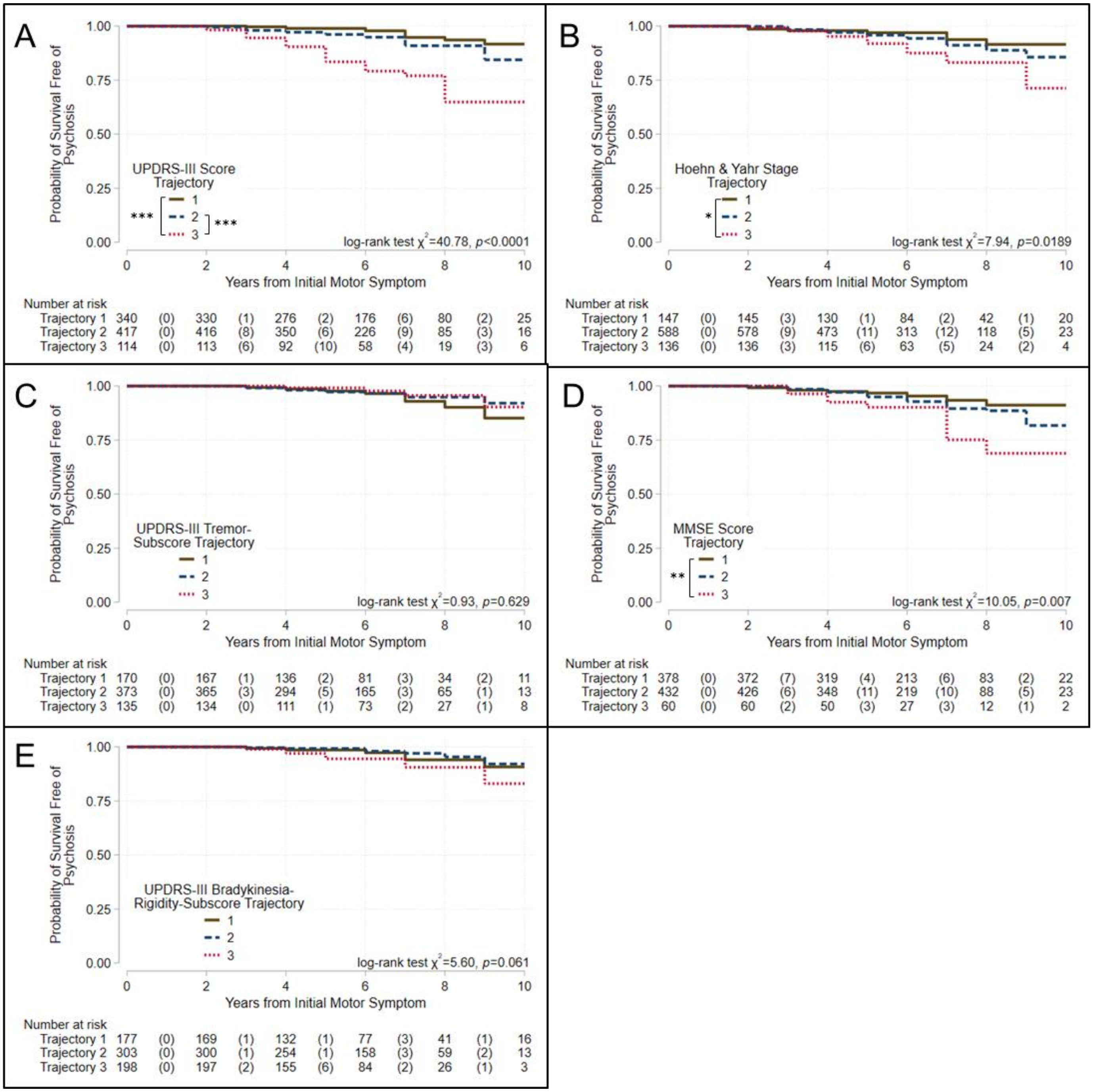

Supplemental Figure S13. Survival free of impulse control disorder Kaplan-Meier analyses for survival free of impulse control disorder (ICD) in trajectory-groups modeled jointly with the predictors: sex, age at motor-symptom onset, pesticide exposure, head injury, diabetes, REM-behavior sleep disorder, family history (Parkinson's disease, dementia, or tremor), initial presentation (tremor-predominant, akinetic/rigid predominant), and for MMSE-score, years of education. (A) UPDRS-III score, (B) Hoehn & Yahr (H&Y) stage, (C) UPDRS−III-tremor subscore, (D) Mini-Mental Status Exam (MMSE), and (E) UPDRS−III-bradykinesia-rigidity subscore. The at-risk table beneath each plot shows the number at-risk at each time point, with the number of failed (outcome reached) events listed in parentheses. Log-rank test results are shown. Asterisks identify pairs of trajectory-groups where outcomes differ in pairwise log-rank tests with a Bonferroni-corrected $p<0.05$ (*) or $p<0.001$ (***). In the UPDRS−III-score trajectory-groups, the outcome of ICD was poorest in Group 3 having the most severe trajectory, and similar but less poor in Groups 1 and 2 having a more benign or an intermediate trajectory, respectively. In the H&Y trajectory-groups, a nearly opposite pattern is seen: it was poorer in Group 1 having a more benign trajectory than in Group 2 having an intermediate trajectory.

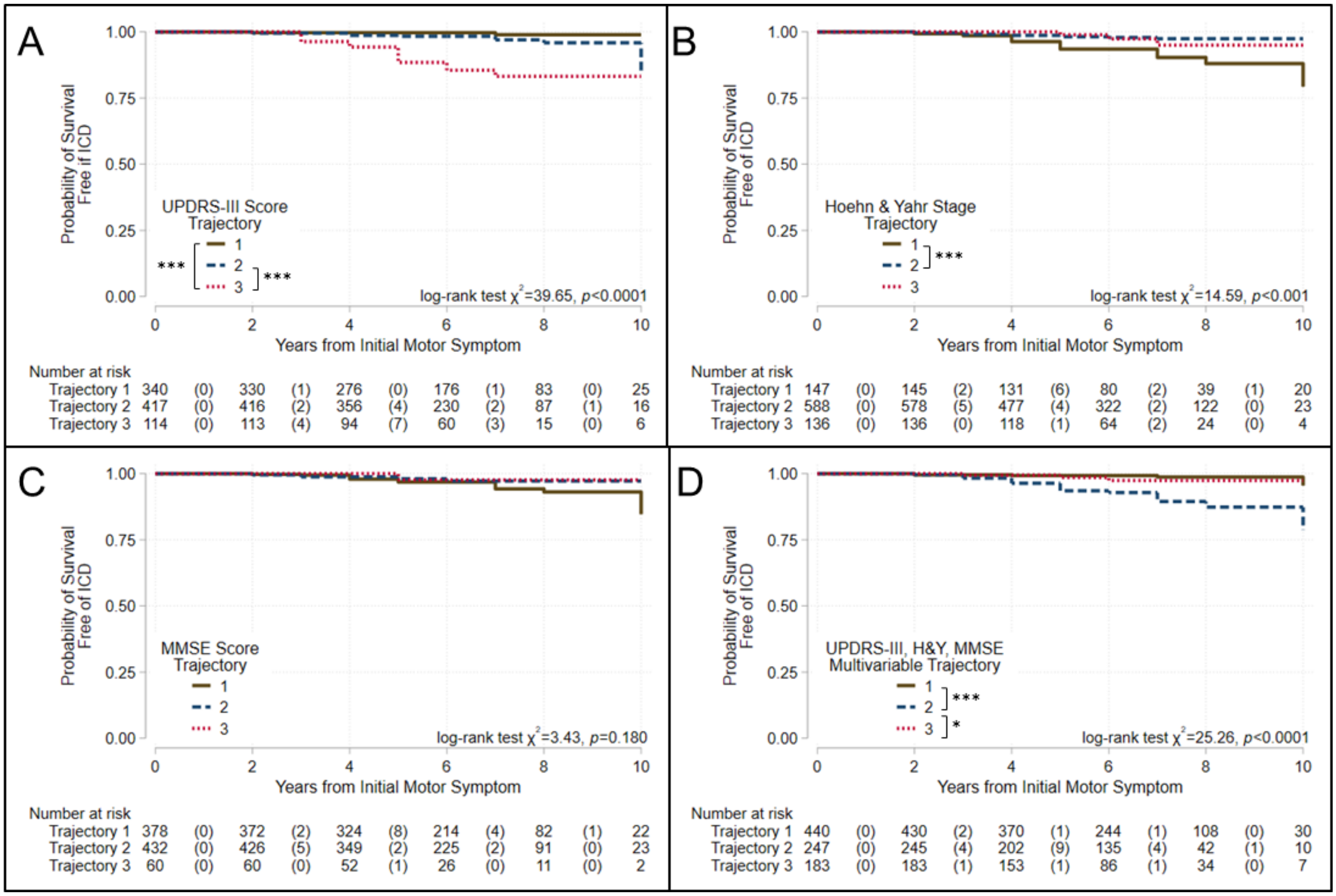

Supplemental Figure S14. Trajectories seen in the LONG-PD cohort when trajectories for three assessments are modeled jointly with the additional predictor *study site* Group-based trajectory modeling using UPDRS-III score, Hoehn and Yahr (H&Y) Stage and Mini-Mental Status Exam (MMSE) score identified three groups (A). Assignment to group-membership trajectories converge (B) with <5% misclassification (C) (dashed teal line) by about five years after the onset of the initial motor symptom. Trajectories were modeled jointly with the predictors: sex, age at motor-symptom onset, years of education, pesticide exposure, head injury, diabetes, REM-behavior sleep disorder, family history (Parkinson's disease, dementia, or tremor), initial presentation (tremor-predominant, akinetic/rigid predominant), and study site.

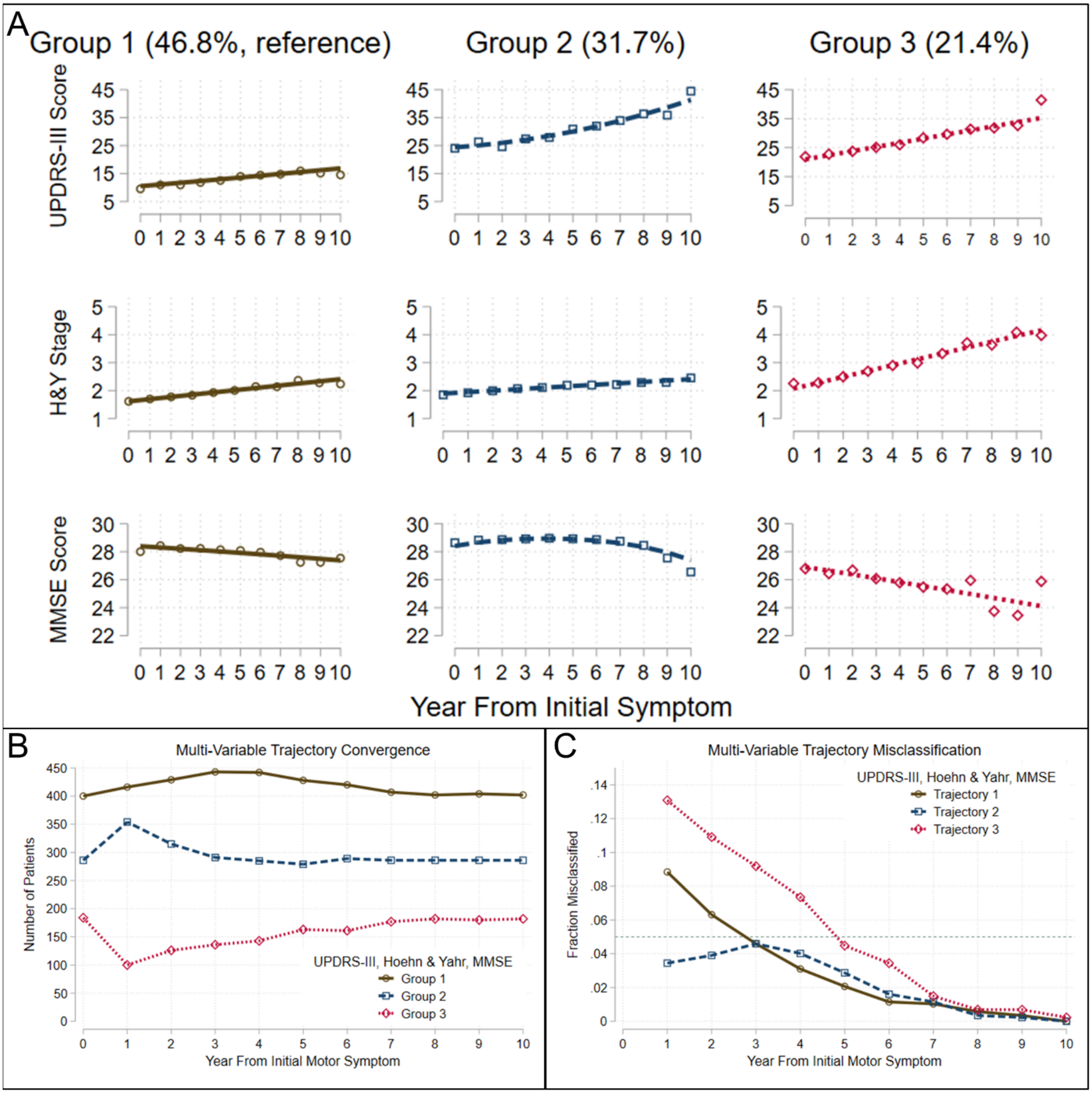

Supplemental Figure S15. Survival free of clinically significant milestones in the groups identified when trajectories for three assessments are modeled jointly with the additional predictor *study site* Kaplan-Meier analyses for survival free of (A) persistent freezing, (B) persistent falls, (C) dysphagia, (D) cognitive impairment, and (E) psychosis in trajectory-groups identified using group-based trajectory models simultaneously considering UPDRS-III total score, Hoehn & Yahr (H&Y) stage, and Mini-Mental Status Exam (MMSE) score. Trajectories were modeled jointly with the predictors: sex, age at motor-symptom onset, years of education, pesticide exposure, head injury, diabetes, REM-behavior sleep disorder, family history (Parkinson's disease, dementia, or tremor), initial presentation (tremor-predominant, akinetic/rigid predominant), and study site. The at-risk table beneath each plot shows the number at-risk at each time point, with the number of failed (outcome reached) events listed in parentheses. Log-rank test results are shown. Asterisks identify pairs of trajectory-groups where outcomes differ in pairwise log-rank tests with a Bonferroni-corrected $p<0.05$ (*), $p<0.01$ (**), or $p<0.001$ (***). The outcomes of persistent freezing, persistent falls, and cognitive impairment are poorest in Group 3, which show the most severe trajectories, and less poor in Group 2, which show intermediate trajectories, compared to Group 1, which shows trajectories that are more benign. The outcomes of dysphagia and psychosis are similar in Groups 2 and 3, but poorer than in Group 1.

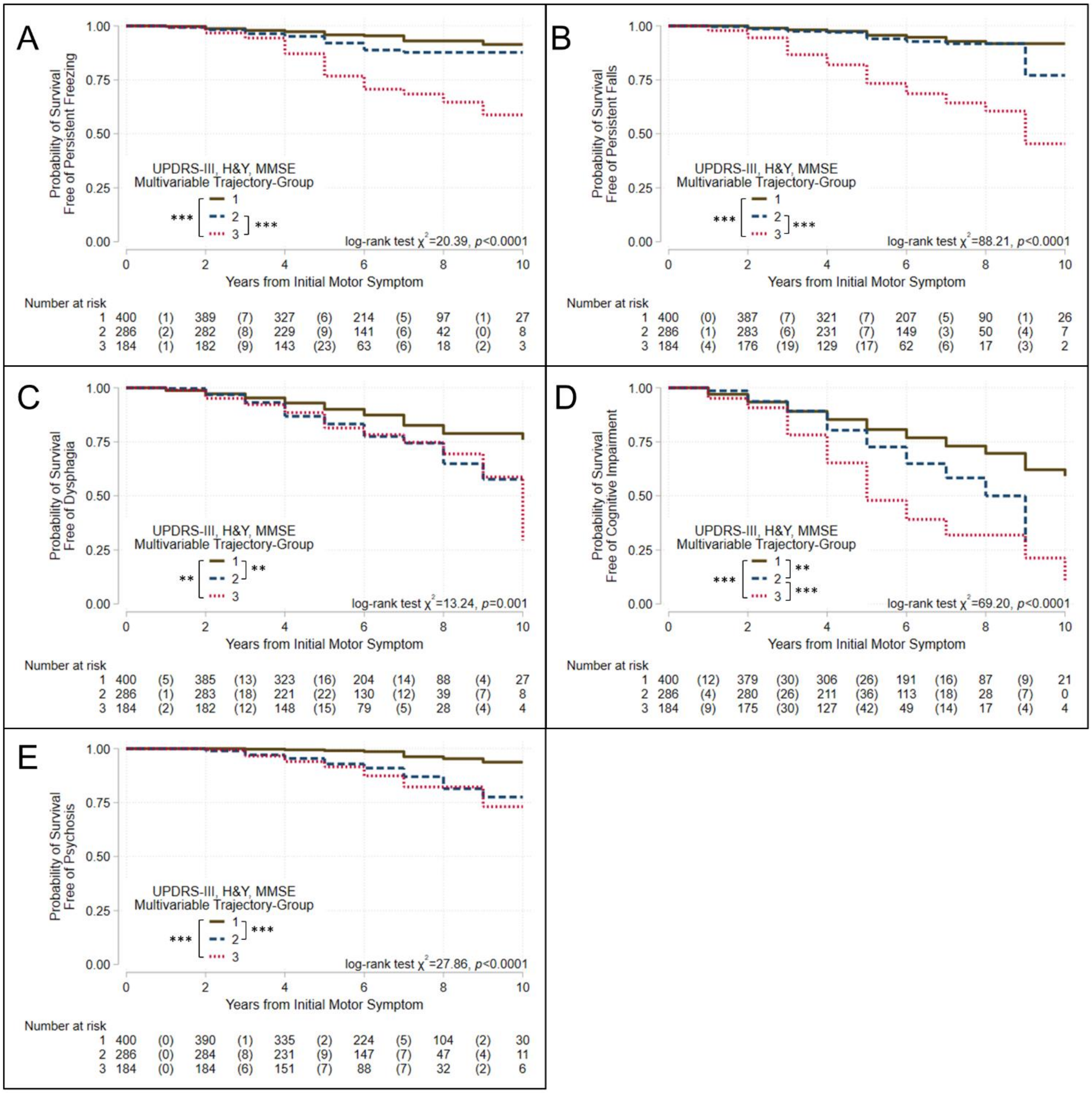

Supplemental Figure S16. Survival free of autonomic symptoms in the groups identified when trajectories for three assessments are modeled jointly with the additional predictor *study site* Kaplan-Meier analyses for survival free of (A) persistent orthostatism and (B) persistent urinary incontinence identified using group-based trajectory models simultaneously considering UPDRS-III total score, Hoehn & Yahr (H&Y) stage, and Mini-Mental Status Exam (MMSE) score. Trajectories were modeled jointly with the predictors: sex, age at motor-symptom onset, years of education, pesticide exposure, head injury, diabetes, REM-behavior sleep disorder, family history (Parkinson's disease, dementia, or tremor), initial presentation (tremor-predominant, akinetic/rigid predominant), and study site. The at-risk table beneath each plot shows the number at-risk at each time point, with the number of failed (outcome reached) events listed in parentheses. Log-rank test results are shown. Asterisks identify pairs of trajectory-groups where outcomes differ in pairwise log-rank tests with a Bonferroni-corrected $p<0.05$ (*), $p<0.01$ (**), or $p<0.001$ (***). All outcomes are similar in Groups 2 and 3, which show intermediate and severe trajectories, respectively, but poorer than Group 1, which shows trajectories that are more benign.

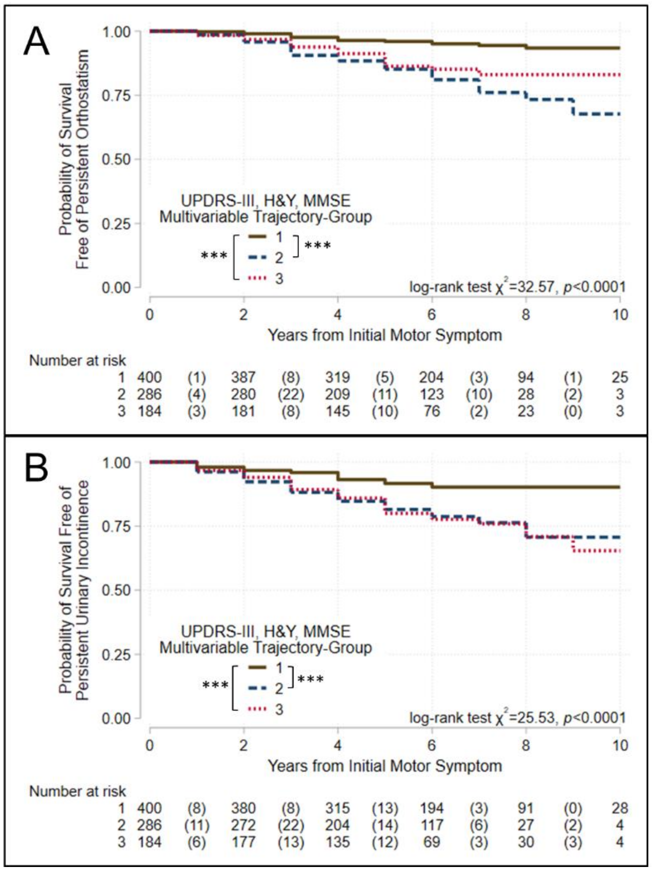

Supplemental Figure S17. Motor-score trajectories seen in the LONG-PD cohort when trajectories are modeled jointly with the additional predictors *years of education* and *study site* Group-based trajectory modeling identified three trajectory-groups for (A) total UPDRS-III scores, (B) UPDRS-III tremor subscores, and (C) UPDRS-III bradykinesia-rigidity subscores. UPDRS-III tremor and bradykinesia-rigidity subscores were available for only 678 participants. Membership in most trajectory-groups converge (panels D-F) with <5% misclassification (dashed teal line) (panels G-I) by four-to-five years after the onset of the initial motor symptom. All converge with <4% misclassification by six years. Trajectories were modeled jointly with the predictors: sex, age at motor-symptom onset, education years, pesticide exposure, head injury, diabetes, REM-behavior sleep disorder, family history (Parkinson's disease, dementia, or tremor), initial presentation (tremor-predominant, akinetic/rigid predominant), and study site.

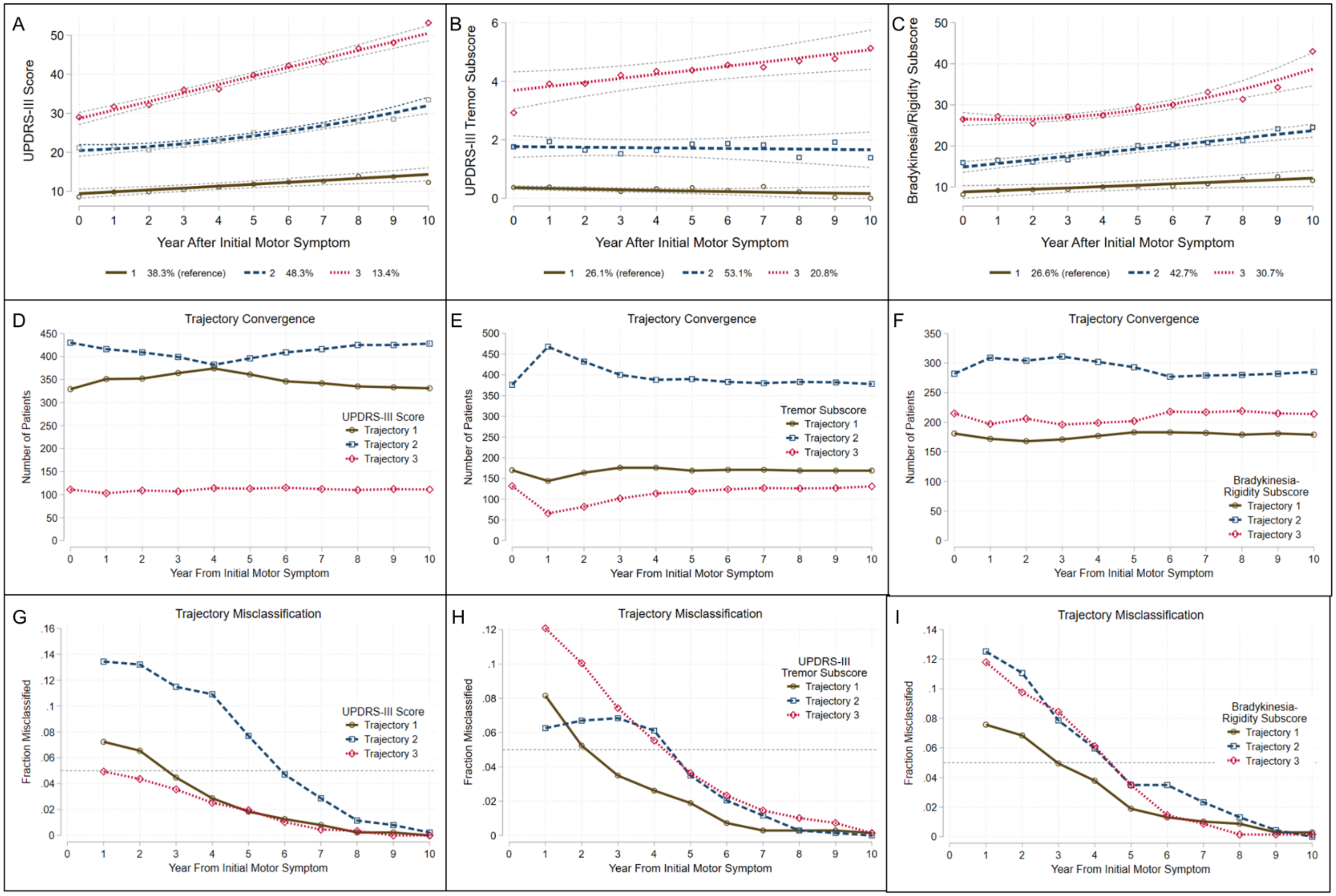

Supplemental Figure S18. Disease-stage and cognitive trajectories seen in the LONG-PD cohort when trajectories are modeled jointly with the additional predictors *years of education* and *study site* Group-based trajectory modeling identified three trajectory-groups for (A) Hoehn and Yahr stage and (B) Mini-mental status exam (MMSE) score. Trajectory-groups for Hoehn and Yahr stage (C, E) and MMSE-score (D, F) converge with <5% misclassification (dashed teal line) by four-to-five years after the onset of the initial motor symptom. Trajectories were modeled jointly with the predictors: sex, age at motor-symptom onset, education years, pesticide exposure, head injury, diabetes, REM-behavior sleep disorder, family history (Parkinson's disease, dementia, or tremor), initial presentation (tremor-predominant, akinetic/rigid predominant), and study site.

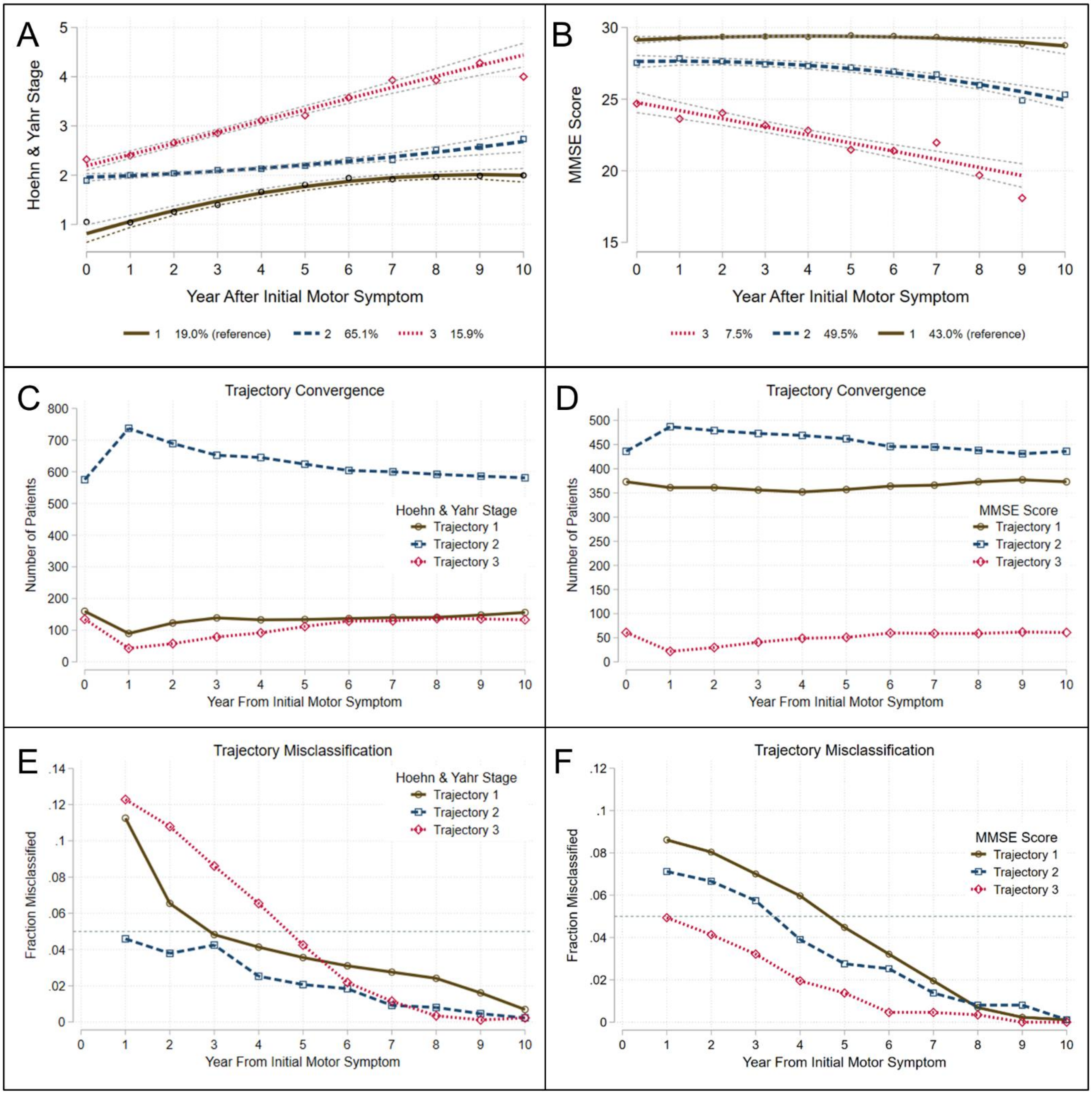

Supplemental Figure S19. Survival free of motor fluctuations when trajectories are modeled jointly with the additional predictors *years of education* and *study site* Kaplan-Meier analyses for survival free of motor fluctuations across trajectory-groups modeled jointly with the predictors: sex, age at motor-symptom onset, education years, pesticide exposure, head injury, diabetes, REM-behavior sleep disorder, family history (Parkinson's disease, dementia, or tremor), initial presentation (tremor-predominant, akinetic/rigid predominant), and study site. (A) UPDRS-III score, (B) Hoehn & Yahr (H&Y) stage, (C) UPDRS−III-tremor subscore, (D) MMSE, and (E) UPDRS−III-bradykinesia-rigidity subscore. An at-risk table beneath each plot shows the number at-risk at each time point, with the number of failed (outcome reached) events listed in parentheses. Log-rank test results shown failed to identify differences between trajectory groups modeled for any assessment.

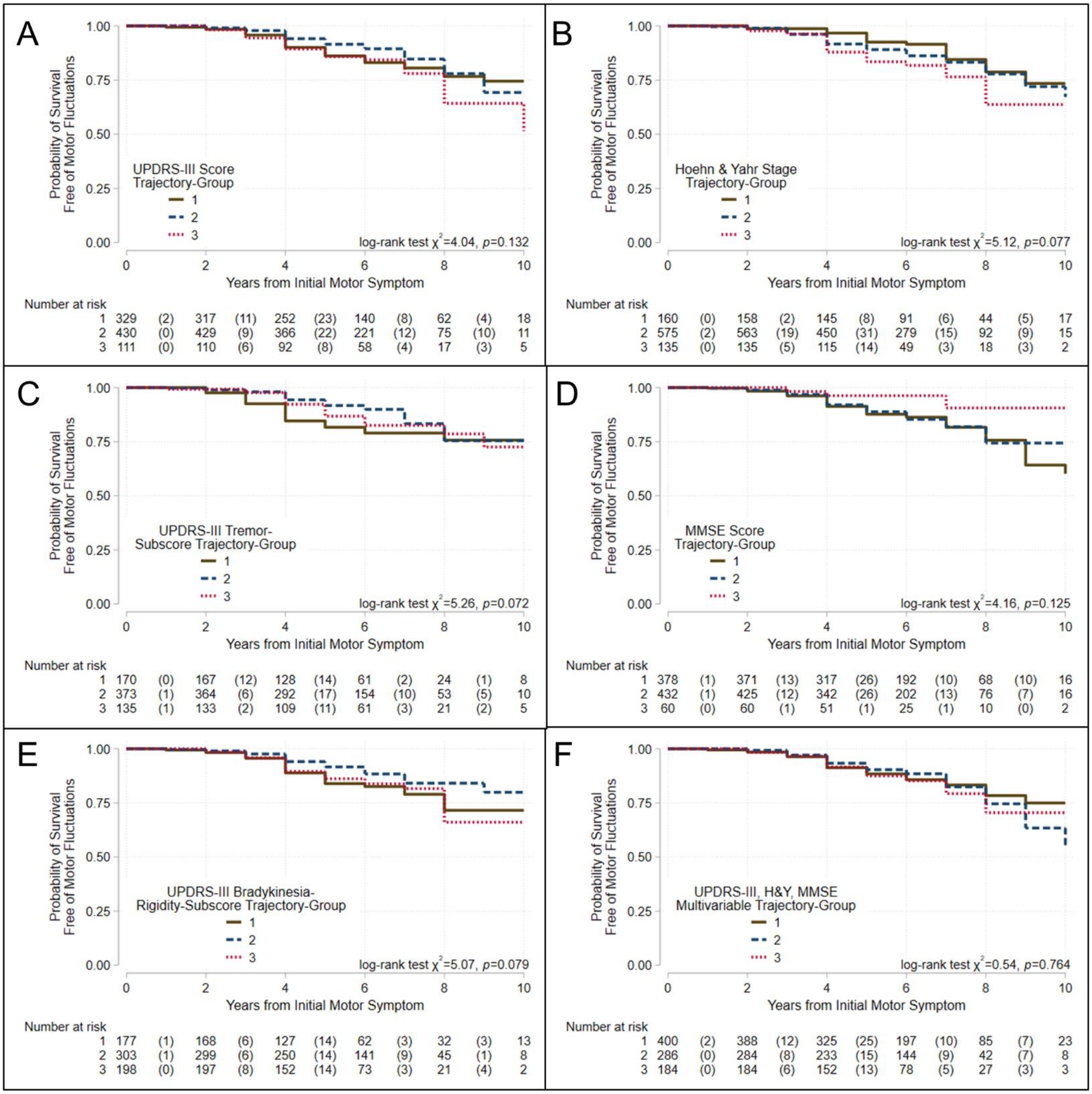

Supplemental Figure S20. Survival free of dyskinesias when trajectories are modeled jointly with the additional predictors *years of education* and *study site* Kaplan-Meier analyses for survival free of dyskinesias in trajectory-groups modeled jointly with the predictors: sex, age at motor-symptom onset, education years, pesticide exposure, head injury, diabetes, REM-behavior sleep disorder, family history (Parkinson's disease, dementia, or tremor), initial presentation (tremor-predominant, akinetic/rigid predominant), and study site. (A) UPDRS-III score, (B) Hoehn & Yahr (H&Y) stage, (C) UPDRS−III-tremor subscore, (D) MMSE, and (E) UPDRS−III-bradykinesia-rigidity subscore. The at-risk table beneath each plot shows the number at-risk at each time point, with the number of failed (outcome reached) events listed in parentheses. Log-rank test results are shown. An asterisk identify pairs of trajectory-groups where outcomes differ in pairwise log-rank tests with a Bonferroni-corrected $p<0.05$. For the UPDRS−III-tremor-subscore trajectory-groups, the outcome of dyskinesias was poorer in Group 1 having a more benign trajectory than in Group 3 having a severe trajectory. For the multivariable trajectory-groups, the outcome of dyskinesias was poorer in Group 3 having a more severe trajectory than in Group 2 having a less severe trajectory. Though the overall log-rank test was significant for differences across the H&Y-trajectory groups, no pairwise differences between these groups were significant after a multiple-testing correction.

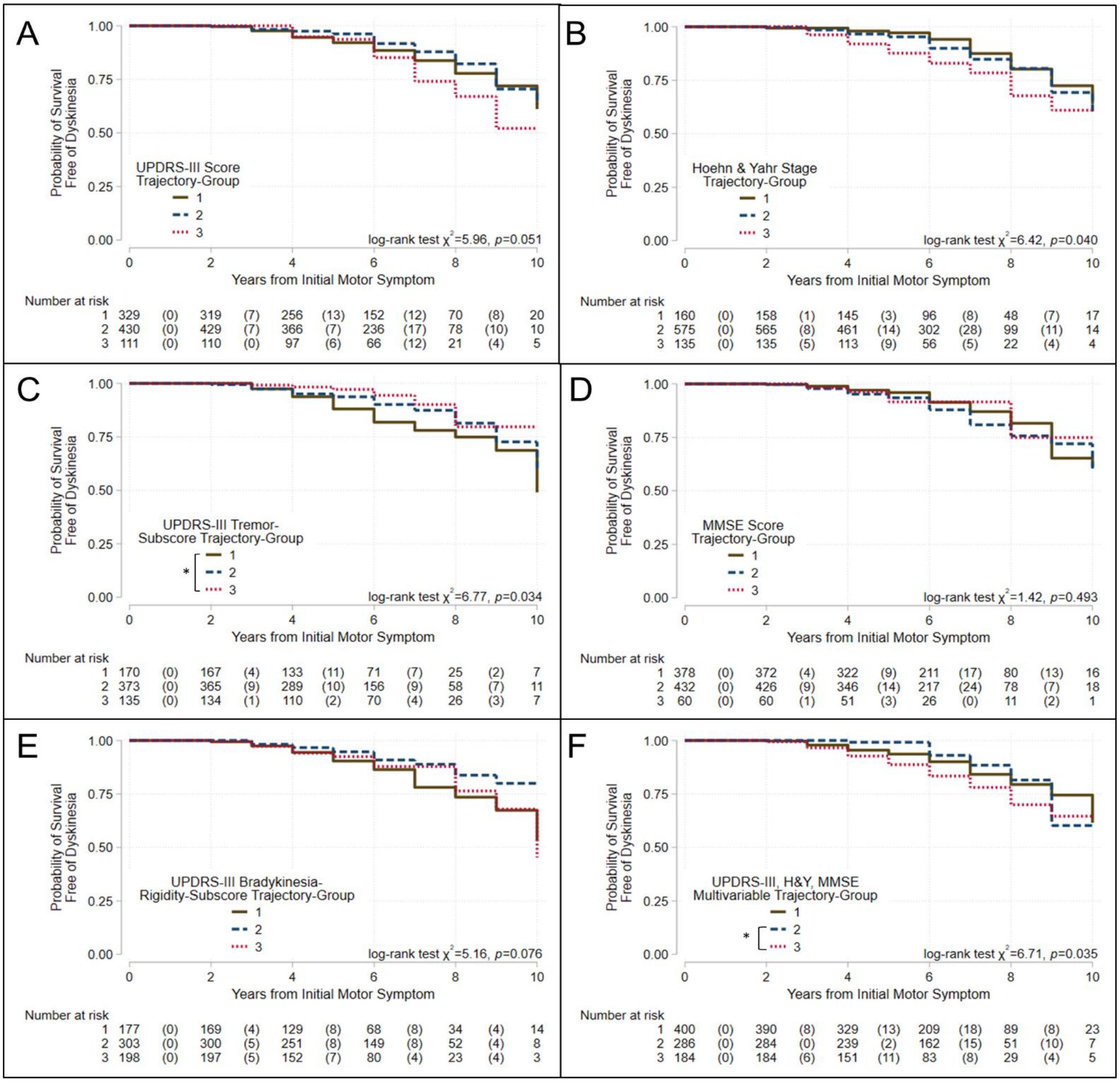

Supplemental Figure S21. Survival free of persistent freezing when trajectories are modeled jointly with the additional predictors *years of education* and *study site* Kaplan-Meier analyses for survival free of persistent freezing in trajectory-groups modeled jointly with the predictors: sex, age at motor-symptom onset, education years, pesticide exposure, head injury, diabetes, REM-behavior sleep disorder, family history (Parkinson's disease, dementia, or tremor), initial presentation (tremor-predominant, akinetic/rigid predominant), and study site. (A) UPDRS-III score, (B) Hoehn & Yahr (H&Y) stage, (C) UPDRS−III-tremor subscore, (D) MMSE, and (E) UPDRS−III-bradykinesia-rigidity subscore. The at-risk table beneath each plot shows the number at-risk at each time point, with the number of failed (outcome reached) events listed in parentheses. Log-rank test results are shown. Asterisks identify pairs of trajectory-groups where outcomes differ in pairwise log-rank tests with a Bonferroni-corrected $p<0.05$ (*), $p<0.01$ (**), or $p<0.001$ (***). For the UPDRS−III-score trajectory-groups, the outcome of persistent freezing was poorest in Group 3 having a severe trajectory, less poor in Group 2 having an intermediate trajectory, and least poor in Group 1 having a more benign trajectory. For the UPDRS-III-bradykinesia-rigidity–subscore trajectory-groups, it was similar in Groups 2 and 3, which have intermediate and severe trajectories, respectively, and poorer than in Group 1 having a more benign trajectory. For the H&Y-stage trajectory-groups, it is poorer in Group 3 having a severe trajectory and similar and less poor in Groups 1 and 2 having more benign and intermediate trajectories, respectively. For the MMSE-score trajectory-groups, the outcome was poorer in Group 3 having a severe trajectory than Group 1 having a more benign trajectory.

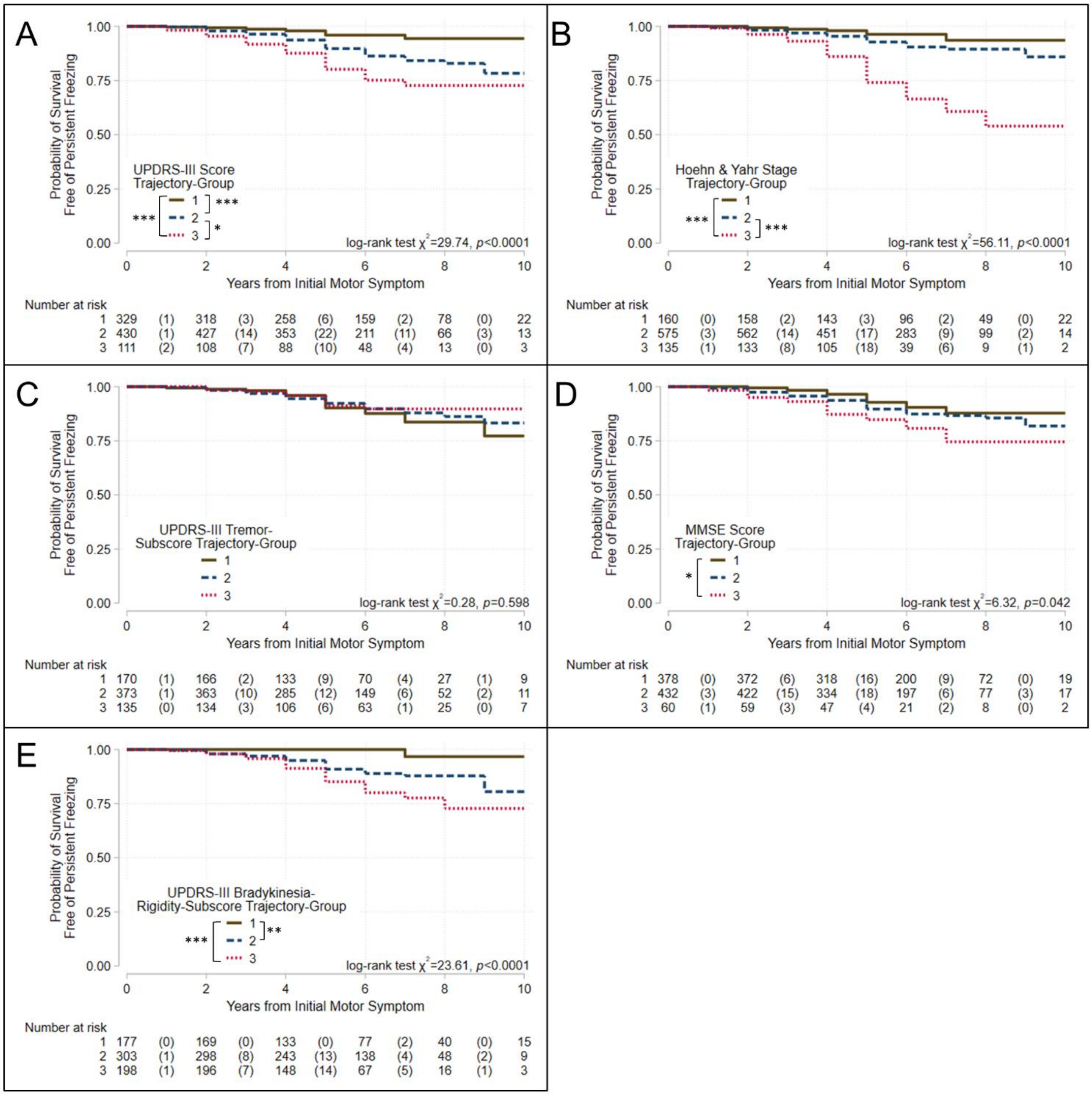

Supplemental Figure S22. Survival free of persistent falls when trajectories are modeled jointly with the additional predictors *years of education* and *study site* Kaplan-Meier analyses for survival free of persistent falls in trajectory-groups modeled jointly with the predictors: sex, age at motor-symptom onset, education years, pesticide exposure, head injury, diabetes, REM-behavior sleep disorder, family history (Parkinson's disease, dementia, or tremor), initial presentation (tremor-predominant, akinetic/rigid predominant), and study site. (A) UPDRS-III score, (B) Hoehn & Yahr (H&Y) stage, (C) UPDRS−III-tremor subscore, (D) MMSE, and (E) UPDRS−III-bradykinesia-rigidity subscore. The at-risk table beneath each plot shows the number at-risk at each time point, with the number of failed (outcome reached) events listed in parentheses. Log-rank test results are shown. Asterisks identify pairs of trajectory-groups where outcomes differ in pairwise log-rank tests with a Bonferroni-corrected $p<0.05$ (*), $p<0.01$ (**), or $p<0.001$ (***). For the H&Y-stage and UPDRS-III score trajectory-groups, the outcome of persistent falls was poorest in Group 3 having the most severe trajectory, less poor in Group 2 having an intermediate trajectory, and least poor in Group 1 having a more benign trajectory. For the MMSE-score and UPDRS-III-bradykinesia-rigidity-subscore trajectory-groups, it was similar in Groups 2 and 3, but poorer than in Group 1 having the more benign trajectory.

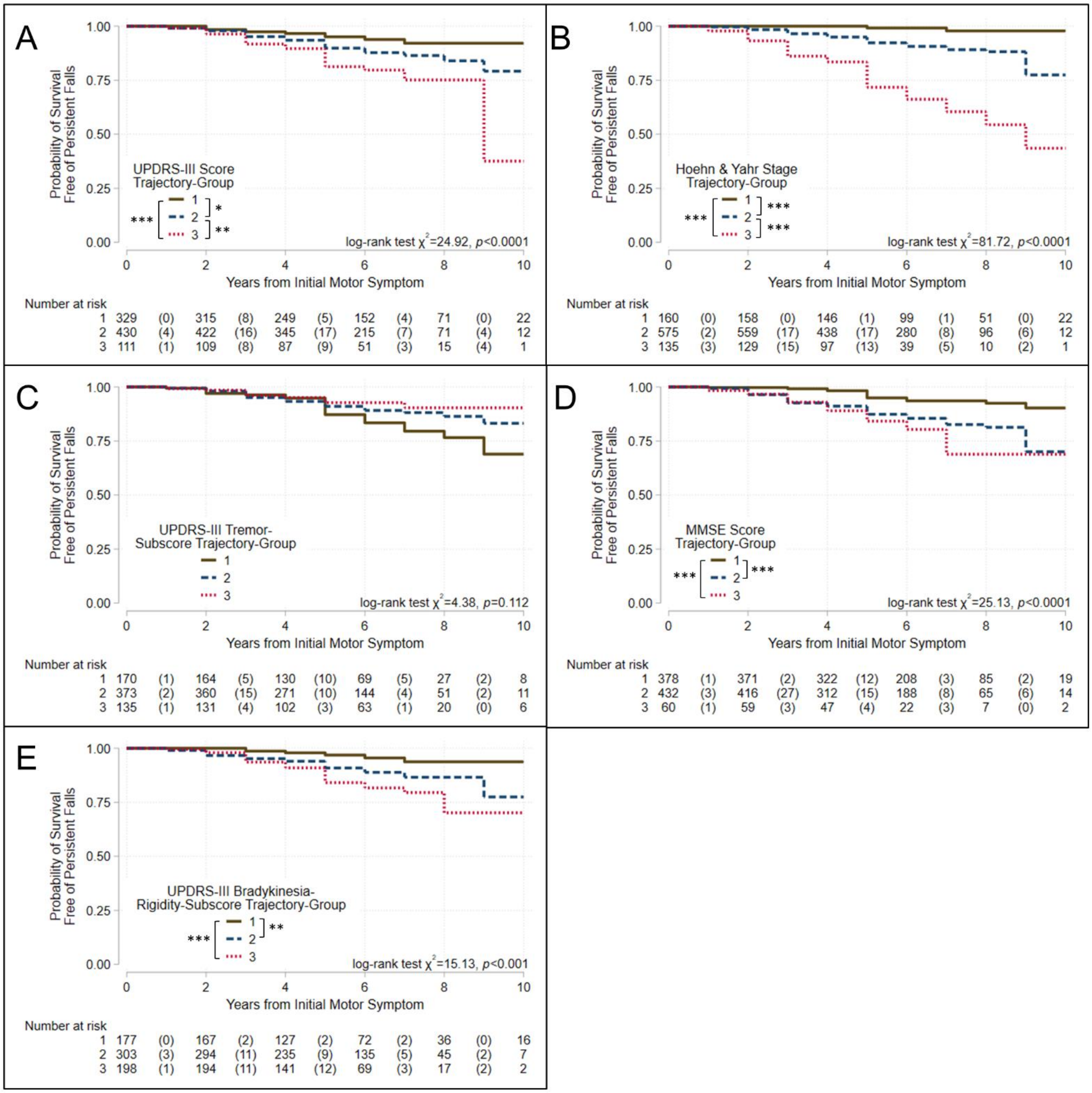

Supplemental Figure S23. Survival free of persistent orthostatism when trajectories are modeled jointly with the additional predictors *years of education* and *study site* Kaplan-Meier analyses for survival free of persistent orthostatism in trajectory-groups modeled jointly with the predictors: sex, age at motor-symptom onset, education years, pesticide exposure, head injury, diabetes, REM-behavior sleep disorder, family history (Parkinson's disease, dementia, or tremor), initial presentation (tremor-predominant, akinetic/rigid predominant), and study site. (A) UPDRS-III score, (B) Hoehn & Yahr (H&Y) stage, (C) UPDRS−III-tremor subscore, (D) Mini-Mental Status Exam (MMSE), and (E) UPDRS−III-bradykinesia-rigidity subscore. The at-risk table beneath each plot shows the number at-risk at each time point, with the number of failed (outcome reached) events listed in parentheses. Log-rank test results are shown. Asterisks identify pairs of trajectory-groups where outcomes differ in pairwise log-rank tests with a Bonferroni-corrected *p*<0.01 (**) or *p*<0.001 (***). For the UPDRS-III score trajectory-groups, the outcome of persistent orthostatism was poorest was Group 3 having the most severe trajectory, less poor in Group 2 having the intermediate trajectory, and least poor in Group 1 having a more benign trajectory.

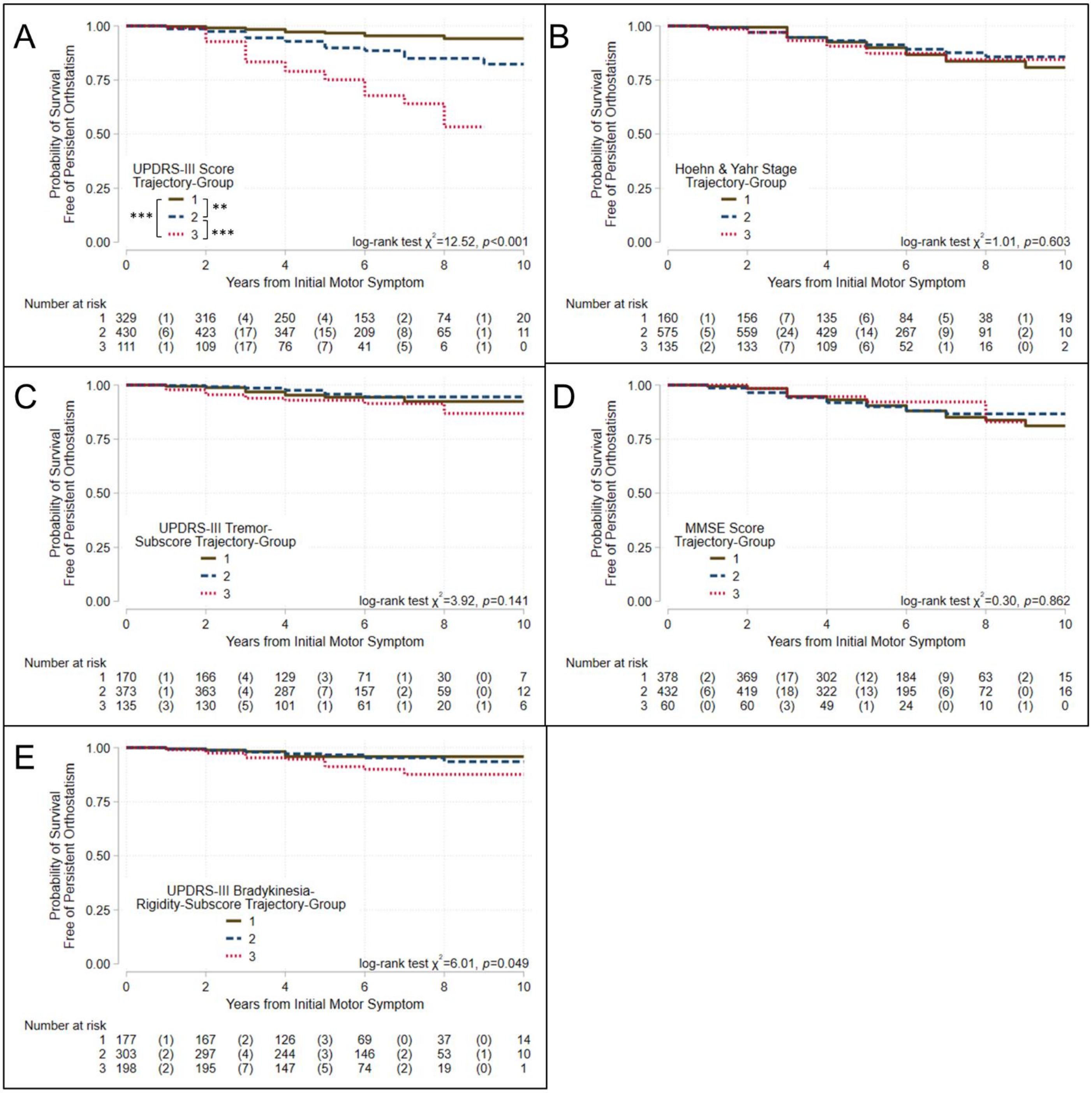

Supplemental Figure S24. Survival free of persistent urinary incontinence when trajectories are modeled jointly with the additional predictors *years of education* and *study site* Kaplan-Meier analyses for survival free of persistent urinary incontinence in trajectory-groups modeled jointly with the predictors: sex, age at motor-symptom onset, education years, pesticide exposure, head injury, diabetes, REM-behavior sleep disorder, family history (Parkinson's disease, dementia, or tremor), initial presentation (tremor-predominant, akinetic/rigid predominant), and study site. (A) UPDRS-III score, (B) Hoehn & Yahr (H&Y) stage, (C) UPDRS−III-tremor subscore, (D) Mini-Mental Status Exam (MMSE), and (E) UPDRS−III-bradykinesia-rigidity subscore. The at-risk table beneath each plot shows the number at-risk at each time point, with the number of failed (outcome reached) events listed in parentheses. Log-rank test results are shown. Asterisks identify pairs of trajectory-groups where outcomes differ in pairwise log-rank tests with a Bonferroni-corrected $p<0.05$ (*) or $p<0.001$ (***). For the UPDRS−III-score trajectory-groups, the outcome of persistent urinary incontinence was poorest was Group 3 having the most severe trajectory, less poor in Group 2 having the intermediate trajectory, and least poor in Group 1 having a more benign trajectory. For the H&Y stage trajectory-groups, it was poorer in Group 3 having the most severe trajectory than in Group 2 have the intermediate trajectory. For the MMSE-score trajectory-groups, it was poorer in Group 3 having the most severe trajectory than in Group 1 having the more benign trajectory.

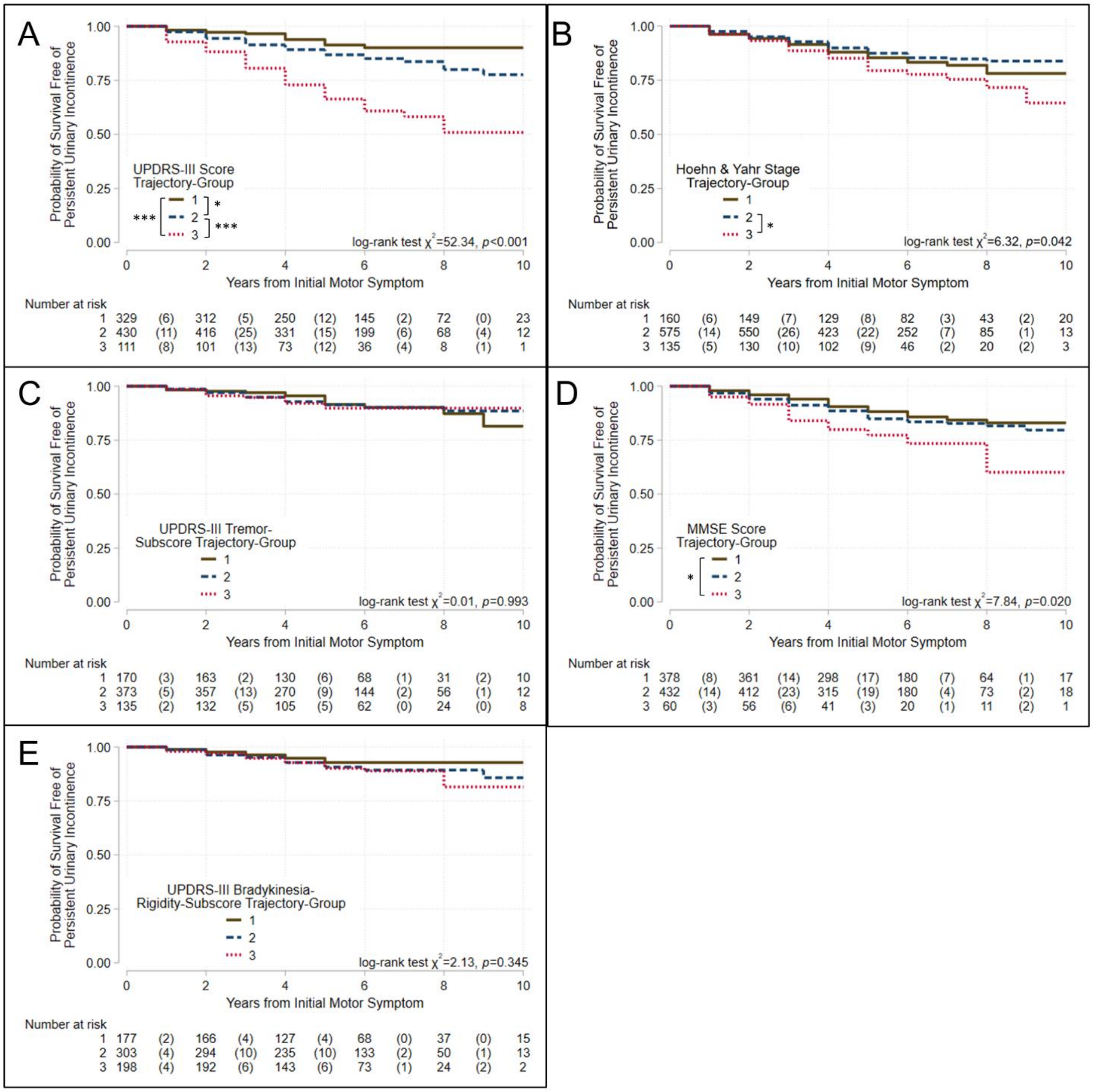

Supplemental Figure S25. Survival free of dysphagia when trajectories are modeled jointly with the additional predictors *years of education* and *study site* Kaplan-Meier analyses for survival free of dysphagia in trajectory-groups modeled jointly with the predictors: sex, age at motor-symptom onset, education years, pesticide exposure, head injury, diabetes, REM-behavior sleep disorder, family history (Parkinson's disease, dementia, or tremor), initial presentation (tremor-predominant, akinetic-rigidity predominant), and study site. (A) UPDRS-III score, (B) Hoehn & Yahr (H&Y) stage, (C) UPDRS−III-tremor subscore, (D) Mini-Mental Status Exam (MMSE), and (E) UPDRS−III-bradykinesia-rigidity subscore. The at-risk table beneath each plot shows the number at-risk at each time point, with the number of failed (outcome reached) events listed in parentheses. Log-rank test results are shown. Asterisks identify pairs of trajectory-groups where outcomes differ in pairwise log-rank tests with a Bonferroni-corrected $p<0.05$ (*), $p<0.01$ (**), or $p<0.001$ (***). For both the UPDRS−III-score and the H&Y-stage trajectory-groups, the outcome of dysphagia was poorest in Group 3 having the most severe trajectory, and similar but less poor in Groups 1 and 2 having more benign or intermediate trajectories, respectively.

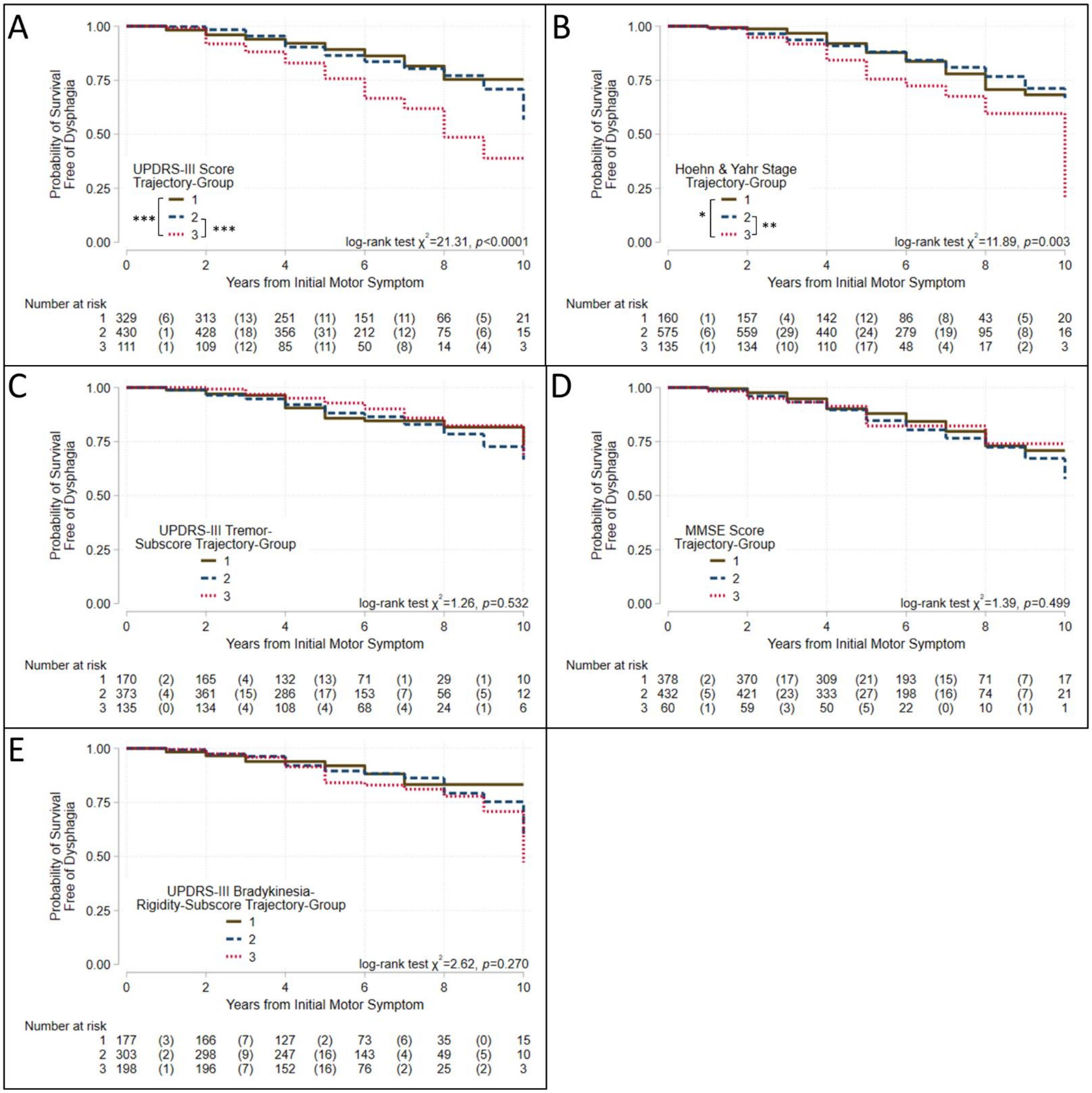

Supplemental Figure S26. Survival free of REM sleep behavior disorder when trajectories are modeled jointly with the additional predictors *years of education* and *study site* The figures present Kaplan-Meier analyses for survival free of REM sleep behavior disorder, excluding individuals presenting with REM sleep disorder at the initial visit. (A) UPDRS-III score, (B) Hoehn & Yahr (H&Y) stage, (C) UPDRS−III-tremor subscore, (D) Mini-Mental Status Exam (MMSE), and (E) UPDRS−III-bradykinesia-rigidity subscore. The at-risk table beneath each plot shows the number at-risk at each time point, with the number of failed (outcome reached) events listed in parentheses. Log-rank test results are shown. Asterisks identify pairs of trajectory-groups where outcomes differ in pairwise log-rank tests with a Bonferroni-corrected p<0.01 (**) or p<0.001 (***). In the UPDRS-III score trajectory-groups, the outcome of REM sleep behavior disorder was similar in Groups 2 and 3 having intermediate and severe trajectories, respectively, and poorer than in Group 1 having a more benign trajectory. Across the UPDRS-III-bradykinesia-rigidity subscore trajectory-groups, it was poorer in Group 3 having a more severe trajectory than in Group 1 having a more benign trajectory. In the multivariable trajectory-groups, the outcome of REM sleep behavior disorder was similar in Groups 1 and 3 having the more benign and severe trajectories, respectively, and poorest than in Group 2 having the intermediate trajectory.

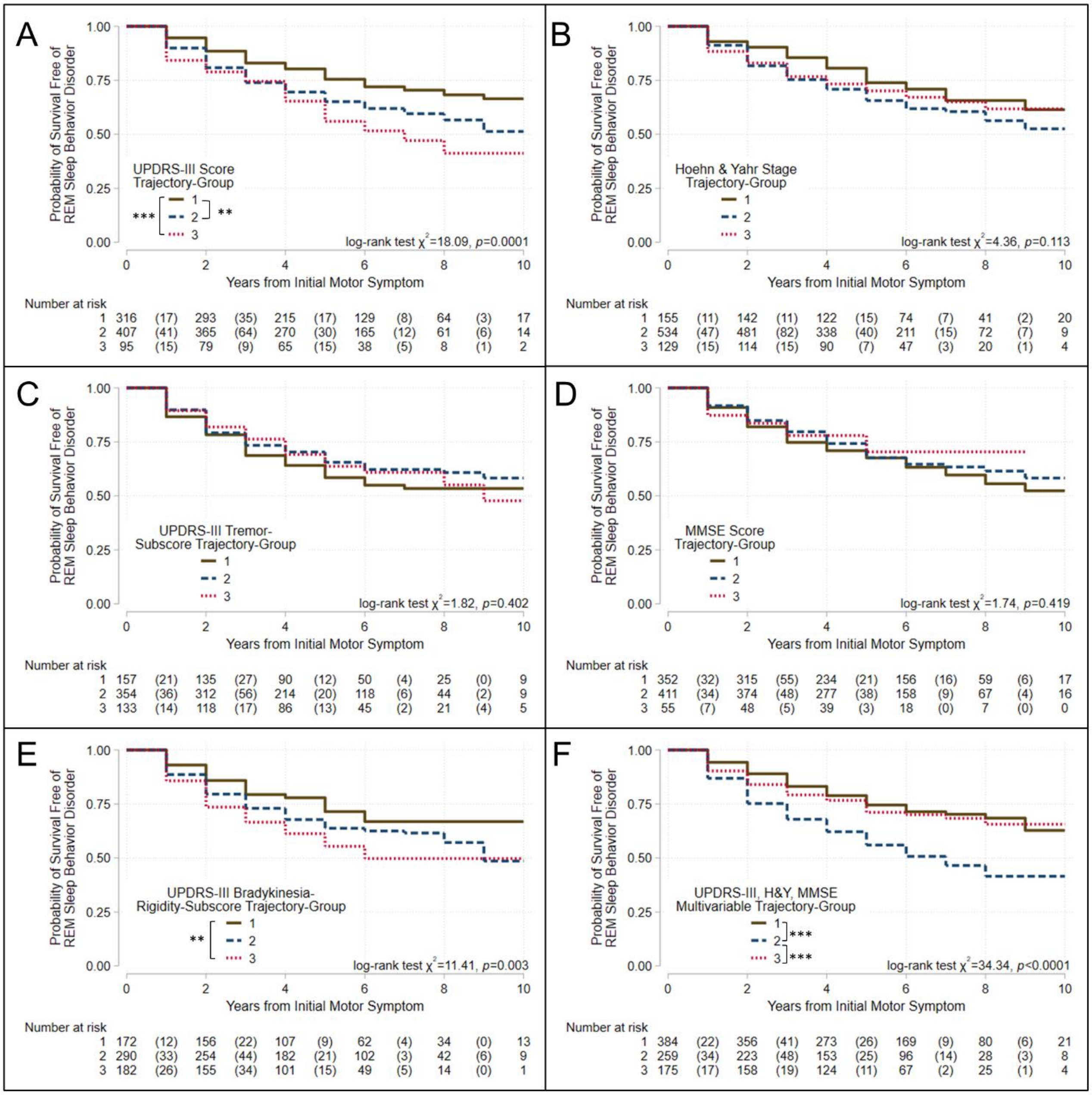

Supplemental Figure S27. Survival free of cognitive impairment when trajectories are modeled jointly with the additional predictor *study site* Kaplan-Meier analyses for survival free of cognitive impairment in trajectory-groups modeled jointly with the predictors: sex, age at motor-symptom onset, education years, pesticide exposure, head injury, diabetes, REM-behavior sleep disorder, family history (Parkinson's disease, dementia, or tremor), initial presentation (tremor-predominant, akinetic/rigid predominant), and study site. (A) UPDRS-III score, (B) Hoehn & Yahr (H&Y) stage, (C) UPDRS−III-tremor subscore, (D) Mini-Mental Status Exam (MMSE), and (E) UPDRS−III-bradykinesia-rigidity subscore. The at-risk table beneath each plot shows the number at-risk at each time point, with the number of failed (outcome reached) events listed in parentheses. Log-rank test results are shown. Asterisks identify pairs of trajectory-groups where outcomes differ in pairwise log-rank tests with a Bonferroni-corrected $p<0.05$ (*), $p<0.01$ (**), or $p<0.001$ (***). In all trajectory-groups except those for the UPDRS−III-tremor subscore, the outcome of cognitive impairment was poorest in Group 3 having the most severe trajectory. In most trajectory-groups, it was less poor in Group 2 having an intermediate trajectory and least poor in Group 1 having a more benign trajectory.

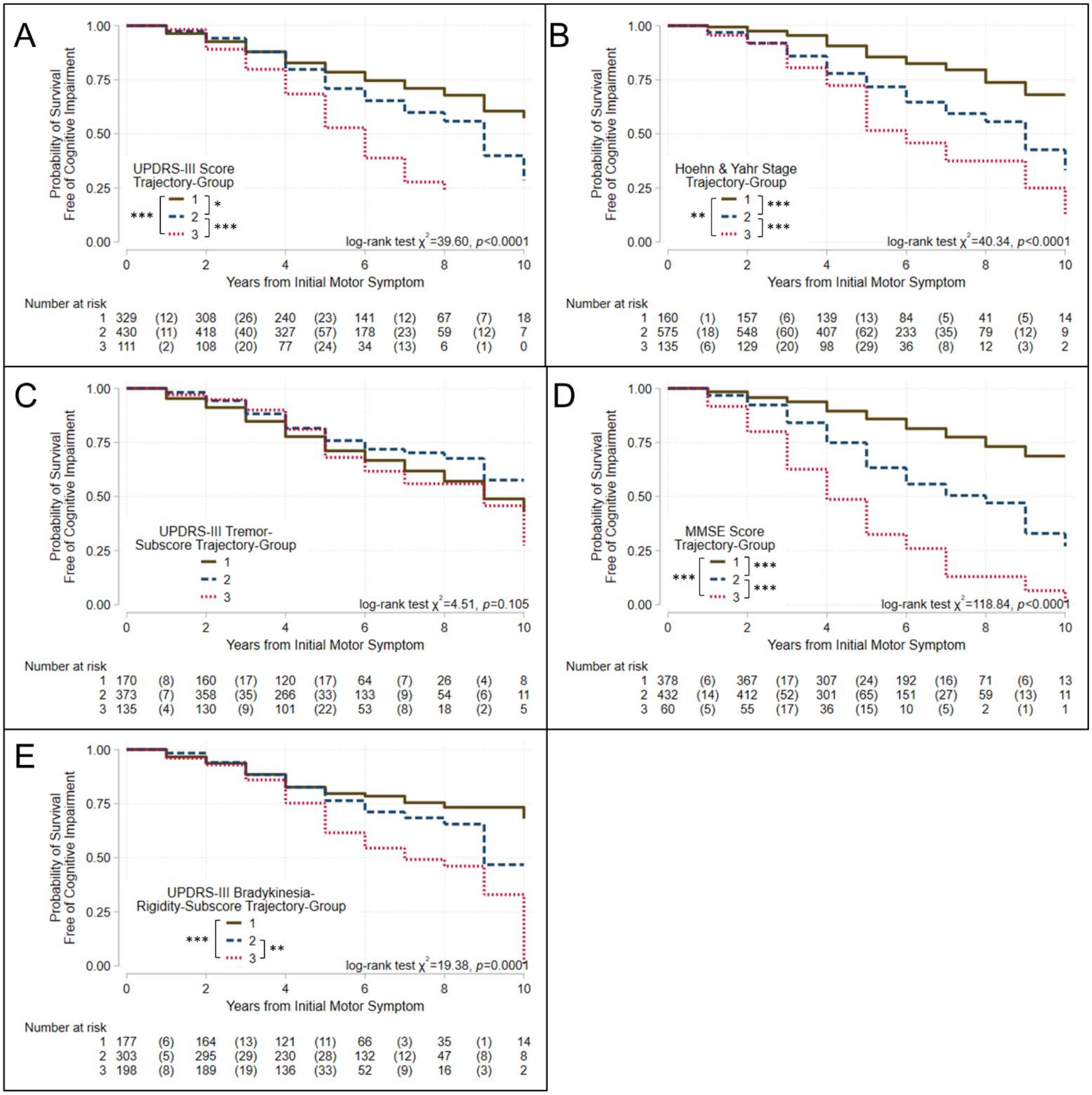

Supplemental Figure S28. Survival free of psychosis when trajectories are modeled jointly with the additional predictors *years of education* and *study site* Kaplan-Meier analyses for survival free of psychosis in trajectory-groups modeled modeled jointly with the predictors: sex, age at motor-symptom onset, education years, pesticide exposure, head injury, diabetes, REM-behavior sleep disorder, family history (Parkinson's disease, dementia, or tremor), initial presentation (tremor-predominant, akinetic/rigid predominant), and study site. (A) UPDRS-III score, (B) Hoehn & Yahr (H&Y) stage, (C) UPDRS−III-tremor subscore, (D) Mini-Mental Status Exam (MMSE), and (E) UPDRS−III-bradykinesia-rigidity subscore. The at-risk table beneath each plot shows the number at-risk at each time point, with the number of failed (outcome reached) events listed in parentheses. Log-rank test results are shown. Asterisks identify pairs of trajectory-groups where outcomes differ in pairwise log-rank tests with a Bonferroni-corrected $p<0.05$ (*), $p<0.01$(**), or $p<0.001$(***). In the UPDRS−III-score trajectory-groups, the outcome of psychosis was poorer in Group 3 having the most severe trajectory, while it was similar, but less poor, in Groups 2 and 1 having intermediate or more benign disease courses, respectively. In the H&Y-stage trajectory-groups, the outcome is poorer in Group 3 having a severe trajectory than in Group 2 having the intermediate trajectory. In the MMSE-score trajectory-groups, the outcome is poorer in Group 3 having a severe trajectory than in Group 1 having a more benign trajectory.

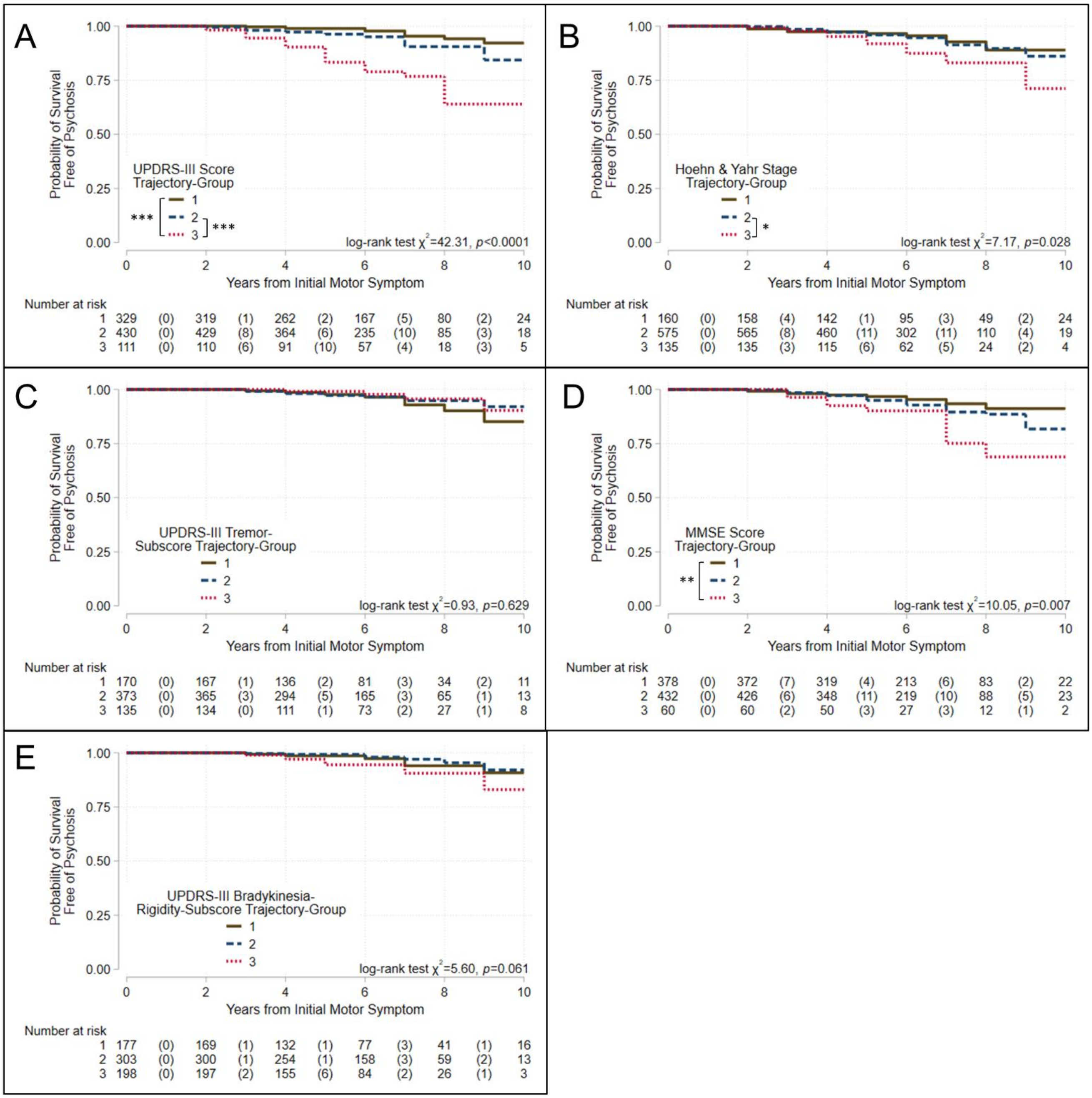

Supplemental Figure S29. Survival free of impulse control disorder when trajectories are modeled jointly with the additional predictors *years of education* and *study site* Kaplan-Meier analyses for survival free of impulse control disorder (ICD) in trajectory- modeled jointly with the predictors: sex, age at motor-symptom onset, education years, pesticide exposure, head injury, diabetes, REM-behavior sleep disorder, family history (Parkinson's disease, dementia, or tremor), initial presentation (tremor-predominant, akinetic/rigid predominant), and study site. (A) UPDRS-III score, (B) Hoehn & Yahr (H&Y) stage, (C) UPDRS−III-tremor subscore, (D) Mini-Mental Status Exam (MMSE), and (E) UPDRS−III-bradykinesia-rigidity subscore. The at-risk table beneath each plot shows the number at-risk at each time point, with the number of failed (outcome reached) events listed in parentheses. Log-rank test results are shown. Asterisks identify pairs of trajectory-groups where outcomes differ in pairwise log-rank tests with a Bonferroni-corrected *p*<0.05 (*), *p*<0.01 (**), or *p*<0.001 (***). For the UPDRS−III-score trajectory-groups, the outcome of ICD was poorest was Group 3 having the most severe trajectory, less poor in Group 2 having the intermediate trajectory, and least poor in Group 1 having a more benign trajectory. In the H&Y trajectory-groups, a nearly opposite pattern is seen: it was poorer in Group 1 having a more benign trajectory than in Group 2 having an intermediate trajectory. In the multivariable trajectory-groups, a different pattern is found. The outcome of ICD was poorer in Group 2 having the intermediate trajectory, while it was similar, but less poor, in Groups 3 and 1 having severe or more benign disease courses, respectively.

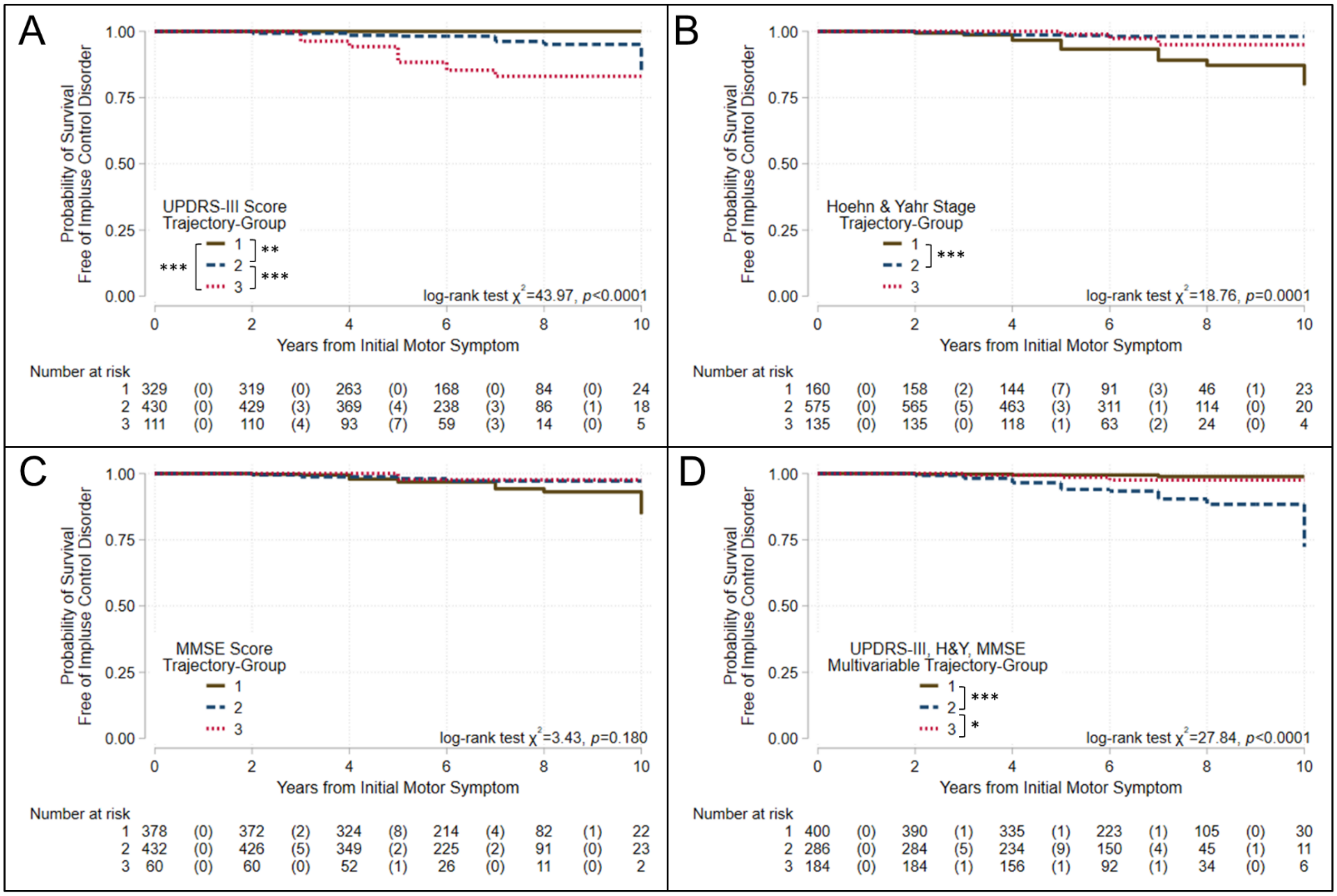

Supplement: Supplementary file 1 [file Data_Sheet_1.PDF]
